# Metabolism and hydrophilicity of the polarised ‘Janus face’ all-*cis* tetrafluorocyclohexyl ring, a candidate motif for drug discovery†
†Electronic supplementary information (ESI) available. CCDC 1817386–1817390. For ESI and crystallographic data in CIF or other electronic format see DOI: 10.1039/c8sc00299a


**DOI:** 10.1039/c8sc00299a

**Published:** 2018-02-19

**Authors:** Andrea Rodil, Stefano Bosisio, Mohammed Salah Ayoup, Laura Quinn, David B. Cordes, Alexandra M. Z. Slawin, Cormac D. Murphy, Julien Michel, David O'Hagan

**Affiliations:** a EaStChem School of Chemistry , University of St Andrews , North Haugh, St Andrews, Fife KY16 9ST , UK . Email: do1@st-andrews.ac.uk; b EaStChem School of Chemistry , University of Edinburgh , Joseph Black Building, David Brewster Road , Edinburgh , EH9 3FJ , UK . Email: julien.michel@ed.ac.uk; c UCD School of Biomolecular and Biomedical Sciences , University College Dublin , Belfield , Dublin , Ireland . Email: cormac.d.murphy@ucd.ie; d Department of Chemistry , Faculty of Science , Alexandria University , P.B. 426 Ibrahimia , Egypt

## Abstract

The metabolism and polarity of the all-*cis* tetra-fluorocyclohexane motif is explored in the context of its potential as a motif for inclusion in drug discovery programmes. Biotransformations of phenyl all-*cis* tetra-, tri- and di- fluoro cyclohexanes with the human metabolism model organism *Cunninghamella elegans* illustrates various hydroxylated products, but limited to benzylic hydroxylation for the phenyl all-*cis* tetrafluorocyclohexyl ring system. Evaluation of the lipophilicities (log *P*) indicates a significant and progressive increase in polarity with increasing fluorination on the cyclohexane ring system. Molecular dynamics simulations indicate that water associates much more closely with the hydrogen face of these Janus face cyclohexyl rings than the fluorine face owing to enhanced hydrogen bonding interactions with the polarised hydrogens and water.

## Abstract 2

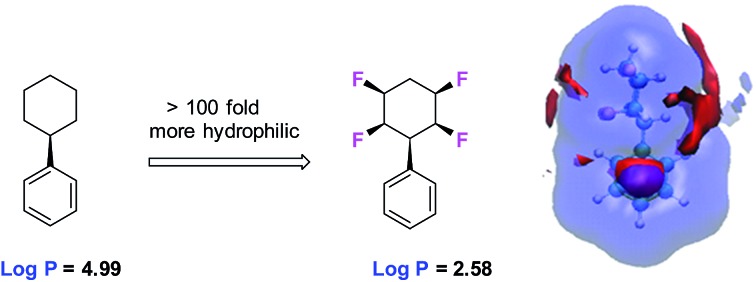
The metabolism and polarity of the all-*cis* tetra-fluorocyclohexane motif is explored in the context of its potential as a motif for inclusion in drug discovery programmes.

## Introduction

Approximately one third of drugs on the market or in development contain at least one fluorine atom^1^ and around a third of herbicides historically,^2^ and half the commercial pesticides introduced in the last period (2010–2016)^3^ contain fluorine atoms. The element is also important in organic materials with applications in next generation displays^4^ and high value materials.^5^ The investigation of new products bearing fluorinated moieties is an ever expanding field, given the particular properties that fluorine bestows on organic compounds.^6^ We have recently synthesised all-*cis* 1,2,4,5-tetrafluorocyclohexane ring systems such as **1** and **2** as a novel motif in organic chemistry.^7^ This tetrafluorocyclohexane isomer displays a particular polar property across the cyclohexane, largely because all of the fluorines are on one face of the ring and there are two 1,3-diaxial C–F bonds, with dipoles orientated parallel to each other.
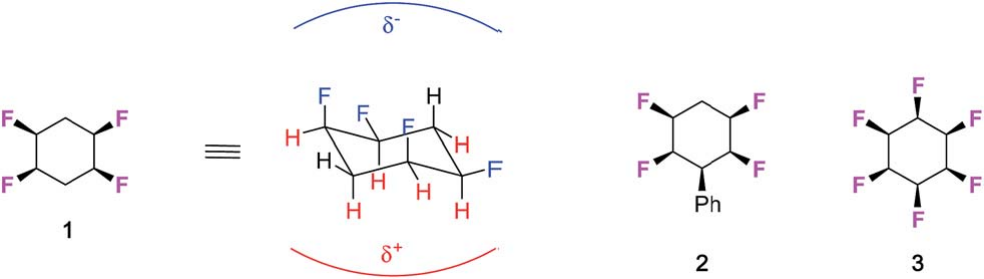


As an extreme example, we extended this concept to the preparation and analysis of all-*cis* hexafluorocyclohexane **3** which is even more polar due to a cyclohexane ring accommodating three axial C–F bonds.8*a* This cyclohexane has been referred to as a ‘Janus’-like molecule,8*b* because of its very well differentiated faces, and recent experimental and theoretical studies have indicated that these rings will coordinate cations to the fluorine face and anions to the hydrogen face, consistent with the electrostatic polarity of the ring system.^9^ The conformational and polar properties of these multi-vicinal fluorinated aliphatics is beginning to attract the attention of the synthesis community and new methods are emerging for their preparation, for example, from the Gilmour,^9^ Jacobsen^10^ and Carreira^11^ laboratories. For the cyclohexanes, a recent report from Glorius's laboratory^12^ has demonstrated the direct catalytic hydrogenation of fluorinated aromatics to generate all-*cis* fluorinated cyclohexanes in a single step, and this methodology promises to make compounds such as cyclohexanes **1** and **3** much more accessible to the organic chemistry community. With these developments in synthesis methods, we believe the cyclohexane motif merits exploration as a candidate substituent for agrochemicals or pharmaceutical drug discovery programmes.

Immediate questions which arise are how will these selectively fluorinated cyclohexane rings be metabolised and how lipophilic are these ring systems. It is commonly understood that increasing the level of fluorination of an organic motif will generally result in increasing its resistance to metabolism at certain sites.^13^ Also, the prevailing dogma is that increased levels of fluorination render a motif more lipophilic and, thus, its introduction would have a tendency to raise log *P* values in a manner detrimental to judicious selection in medicinal chemistry. However, it is more complex than that, and Müller and Carreira have exemplified this extensively in recent contributions *e.g.* mapping log *P*s of RCH_3_ compounds through progressive fluorination to RCF_3_, where intermediate fluorinations (RCH_2_F & RCF_2_H) decrease lipophilicity.^14^ It is a feature of these ring systems,^15^ where the fluorines have a relative stereochemistry such that they are all on one face of the cyclohexane, that the rings become polar, and thus increasing fluorination could reasonably increase hydrophilicity. Thus we set out to explore the nature of these ring systems in the context of their properties and potential as a novel motif for inclusion in bioactive research programmes. To that end we focus on phenylcyclohexane **2**, because it is readily prepared7*c* and has been shown to be amenable to a range of synthetic transformations and diversification.^16^,7b The study compared the metabolism of **2** to close analogues **4–7** by incubation with the human metabolism model organism *Cunninghamella elegans*.^17^ Lipophilicity trends (log *P*) were also explored comparing cyclohexanes with four, three, two and no fluorine atoms. Lastly, a molecular dynamics simulation study was carried out to elucidate the structural basis of the observed lipophilicity trends.
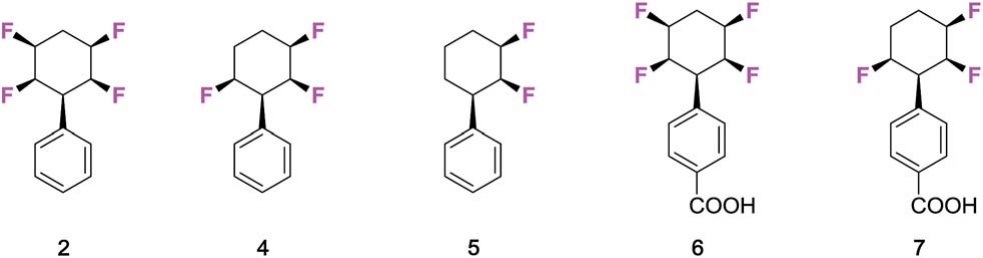


## Results and discussion

### Biotransformations with *Cunninghamella elegans*

The fungus *Cunninghamella elegans* represents a well-established model for drug metabolism in mammals due to its ability to biotransform and degrade a wide range of xenobiotics.^18^ The organism contains a range of cytochrome P_450_ enzymes and this gives an oxidative metabolic profile which mimics phase-I oxidative metabolism. In order to investigate how the tetrafluorocyclohexyl motif may be metabolised, we have explored the incubation of phenyl tetrafluorocyclohexanes and also compounds with three and two fluorine atoms. Five compounds were investigated in total, three of which were the phenyl derivatives **2**, **4** and **5,** and two were the benzoic acid derivatives **6** and **7**.7*d* Incubations with *C. elegans* were carried out in triplicate in submerged liquid cultures. In each case, the incubations were worked up after three days and products were extracted and analysed.

Phenylcyclohexane **2** gave rise to only one obvious metabolite in a conversion of around 30%. This product arose by direct hydroxylation at the benzylic position of **2** to give benzyl alcohol **8**. Only one product as a single isomer could be detected, with the hydroxyl group configured *anti* to the adjacent fluorine atoms of the cyclohexane ring. The identity of **8** and its stereochemistry was confirmed by X-ray structure analysis.

**Fig. 1:**
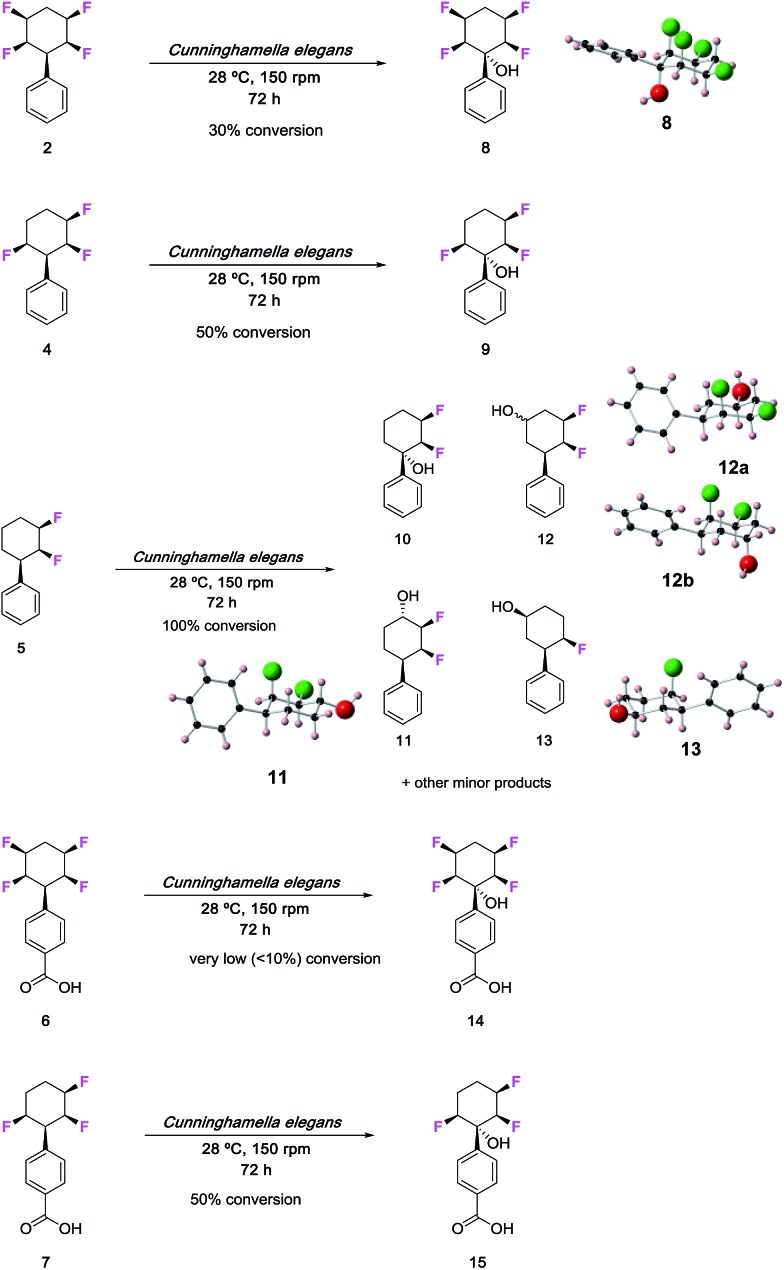
Biotransformations of selectively fluorinated phenyl fluorocyclohexanes **2** and **4–7** by *C. elegans*. Some of the products were crystalline and amenable to X-ray structure analysis.

Phenyl trifluorocyclohexane **4** was similarly incubated with the fungus and it too gave rise to the analogous benzyl hydroxylated product **9**. The extent of microbial conversion was approximately 50% after the three day incubation. The residual **4** was assayed for enantiomeric purity by chiral HPLC, and it was shown to be almost racemic, thus there is no indication that the microbial hydroxylation was significantly enantioselective. Finally in this series, difluorocyclohexane **5** was subject to a similar incubation with *C. elegans*. This compound was completely and extensively metabolised, and it generated a much greater product profile of which compounds **10–13** were isolated. Compounds **11–13** were characterised by X-ray crystallography as illustrated in Fig. 1. Monohydroxylated products **10–12**, can be rationalised by direct methylene P_450_ type hydroxylations, however the monofluorinated cyclohexanol **13** is less easily rationalised and presumably arises from a series of biotransformations involving fluoride elimination. More generally, it is clear that removal of two of the ring fluorines from positions **2** and **3** of the phenyl all-*cis* tetracyclohexyl ring system has rendered the aliphatic ring much more susceptible to metabolism.

The benzoic acids **6** and **7** were also incubated with *C. elegans*. The tetrafluorocyclohexyl benzoic acid **6** was poorly biotransformed and only a very low conversion to alcohol **14** was obvious after the three day incubation. Trifluorocyclohexyl benzoic acid **7** was more readily transformed, but only to benzylalcohol **15** (∼50% conversion). This product was isolated and crystallised and X-ray analysis confirmed its structure. Again, in order to explore any enantioselectivity for this biotransformation, the methyl ester of the residual carboxylic acid **7** was analysed by chiral HPLC and this indicated a very low enantioselectivity, thus in a similar outcome to substrate **4**, there was no obvious selectivity for **7** by the hydroxylation enzyme involved.

### Lipophilicity study of selectively fluorinated phenyl cyclohexanes

**Fig. 2:**
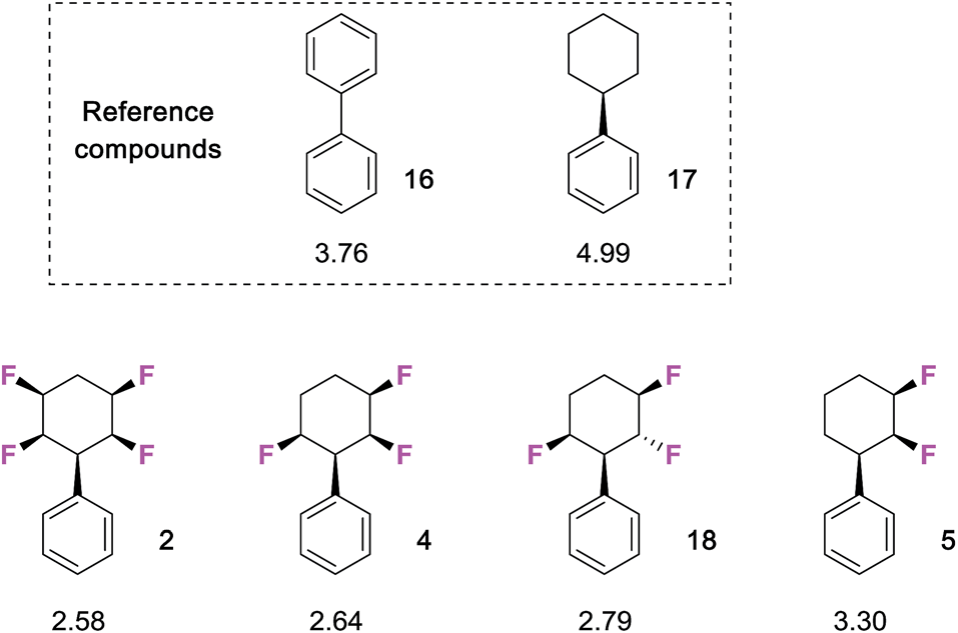
Measured^20^ log *P* values for compounds selectively fluorinated phenylcyclohexanes and reference compounds **16** and **17**. Increasing fluorination lowers log *P* consistent with increasing hydrophilicity.

An important measure of the druggability of a substituent is its lipophilicity,^19^ and given the polarity of the phenyl all-*cis* tetrafluorocyclohexyl moiety it was of interest to explore the relative log *P*s of various analogous compounds. log P's were measured by reverse phase HPLC (AcCN 60%: water 40%, with TFA 0.05%), as previously described.^20^ The measured log P's of a series of phenyl fluorocyclohexane derivatives are summarised in Fig. 2 and against a series of compounds, of known log *P* values, which were re-measured for comparison, including biphenyl **16** and phenylcyclohexane **17.**

It is clear that there is a significant reduction in log *P* with increasing fluorination. Phenyldifluorocyclohexane **5** (log *P* 3.30) is significantly more polar than the phenylcyclohexane (log *P* 4.99), and then both the tri-and tetra- fluoro cyclohexanes progressively increase in polarity (log *P*s of 2.64 and 2.58 respectively) with additional fluorine atoms. An interesting comparison on log *P*s can be made with the two trifluorinated stereoisomers **4** and **18**. Compound **4** is more polar, and this presumably arises as it has a preferred diaxial arrangement of the C2 and C6 C–F bonds.7*c* This parallel alignment can be expected to increase the molecular dipole relative to isomer **18** which has one of these fluorines lying in an equatorial orientation.

**Fig. 3:**
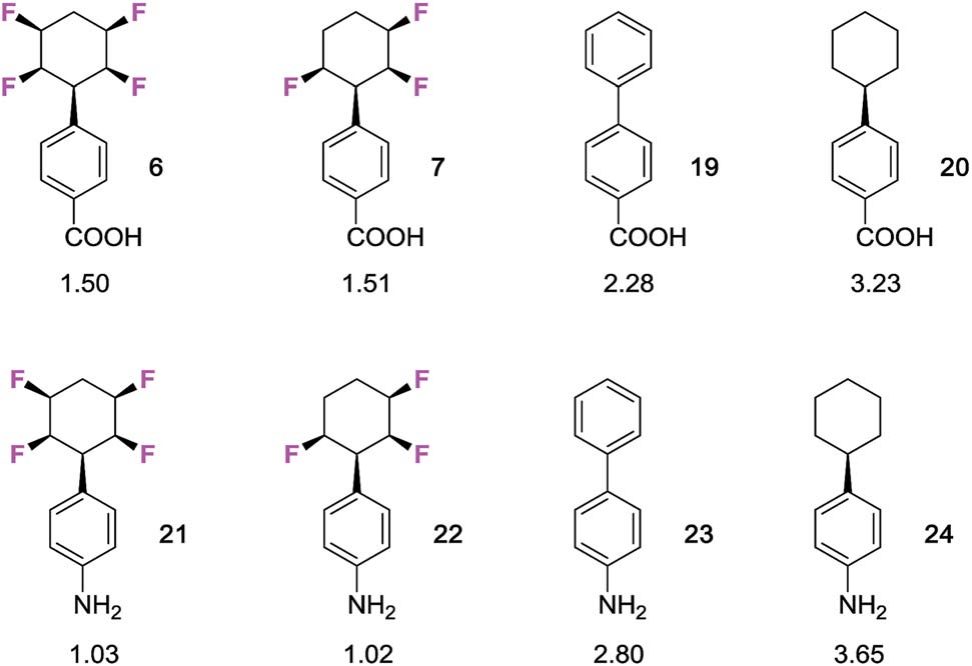
Measured^20^ log *P* values for benzoic acid and aniline derivatives of selectively fluorinated cyclohexanes.

The study extended to substituted aryls of the benzoic acids **6** and **7** and the anilines **21** and **22**(ref. 7b) as illustrated in Fig. 3. In each case both the trifluoro- and tetrafluoro- cyclohexanes are around two log *P* units more lipophilic than the nonfluorinated cyclohexanes **20** and **24**, whereas the phenyl derivatives **19** and **23** lie in between. There is a clear trend that selective fluorinations around the ring increases the polarity of the cyclohexane.

### Computational analysis of lipophilicity trends

**Fig. 4:**
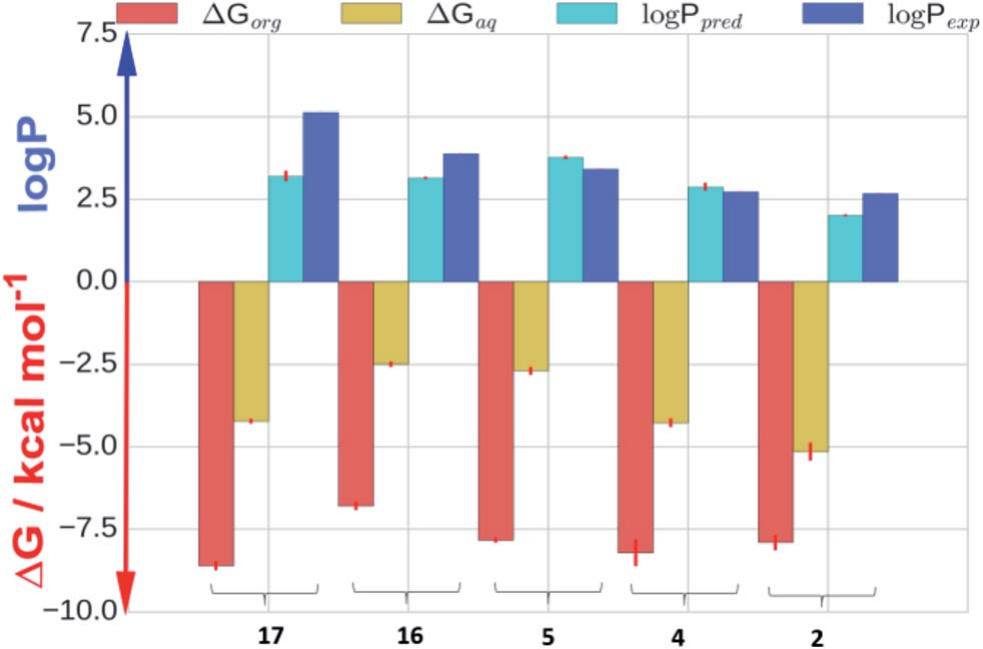
The positive *y*-axis depicts a comparison between calculated (cyan) and measured (blue) log *P* values for compounds **2**, **4**, **5**, **16** and **17**. The negative *y*-axis depicts calculated solvation free energy in cyclohexane, Δ*G*_org_ (red), and aqueous, Δ*G*_aq_ (yellow), phases.

Molecular dynamics (MD) simulations were carried out for phenylcyclohexanes **2**, **4**, **5**, **16** and **17** to clarify the mechanisms by which progressive fluorination decreases lipophilicity. log *P*s were predicted by computing absolute solvation free energies in aqueous and organic phases using explicit solvent molecular dynamics simulations.^21^Fig. 4 shows a comparison of calculated (log *P*_pred_) and measured (log *P*_exp_) log *P* values, as well as calculated solvation free energies in aqueous (Δ*G*_aq_) and cyclohexane (Δ*G*_org_) phases. Overall, the log *P* calculations are in good agreement with the experimental data (Kendall tau 0.5 ± 0.1 and mean unsigned error 0.77 ± 0.07 log *P* units). Inspection of the solvation free energies shows that the trend for decreased log *P* upon increased fluorination is due to a more rapid decrease in solvation free energies in the aqueous phase (from *ca.* –2.5 to –5.2 kcal mol^–1^ for **16** and **2** respectively) *vs.* the cyclohexane phase (*ca.* –7.5 kcal mol^–1^ for all compounds).

Further insights were investigated to help rationalise the calculated differences in hydration free energies by grid-cell theory (GCT) analyses of the MD simulation trajectories.^22^ GCT is a MD trajectory post-processing method that spatially resolves the water contribution to enthalpies, entropies and free energies of the hydration for small molecules, host/guests and protein-ligands complexes.^23^

**Fig. 5:**
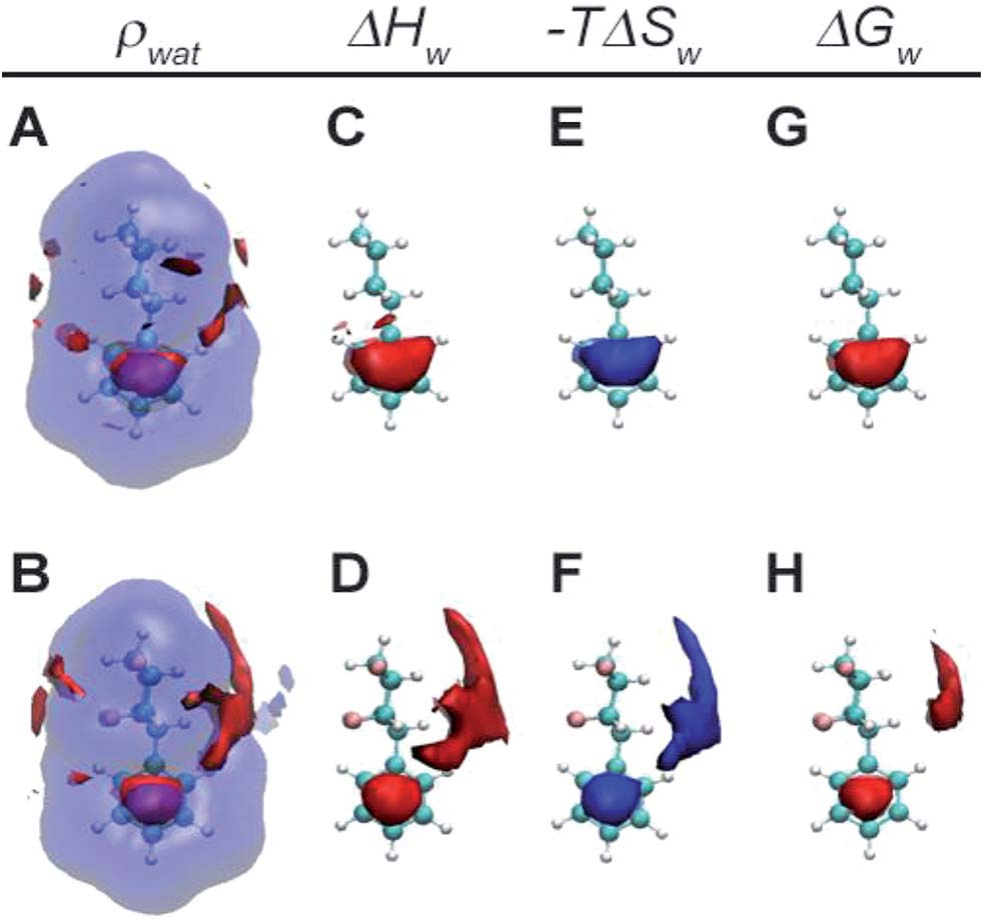
Spatial resolution of hydration thermodynamics around **17** and **2**. Panels A and B show isocontours for density (red: *ρ*_wat_ > 2.33 bulk density, blue: *ρ*_wat_ < 0.5 bulk density). Panels C and D show isocontours for regions where water is enthalpically stabilised with respect to bulk water (red: Δ*H*_w_ < –0.0055 kcal mol^–1^ A^–3^). Panels E and F show isocontours for regions where water is entropically destabilised with respect to bulk water (blue: –*T*Δ*S*_w_ > 0.0033 kcal mol^–1^ A^–3^). Panels G and H show isocontours for regions where water is more stable than bulk water (red: Δ*G*_w_ < –0.0055 kcal mol^–1^ A^–3^).

Fig. 5 depicts spatially resolved hydration thermodynamics around the non-fluorinated cyclohexane **17** and the tetrafluorinated cyclohexane **2**. Comparison of water density contours show water structuring above and below the π-cloud of the phenyl ring due to the expected weak hydrogen bonding interactions in this region. In addition the four fluorine atoms in **2** induce further structuring of water around the cyclohexyl moiety, with a more pronounced effect around the hydrogen face of the cyclohexane (panels A and B). Owing to the different polarities of the cyclohexyl ring in **2**, water near the fluorine-face preferentially orients hydrogen atoms towards the ring, whereas water near the hydrogen-face preferentially orients oxygen atoms towards the ring. Water near the hydrogen-face is more enthalpically stabilised and entropically destabilised with respect to bulk, whereas the energetics are not significantly different from the bulk in the vicinity of the fluorine face (panels C and D and E and F). Overall favourable enthalpic contributions offset unfavourable entropic contributions for water near the hydrogen face and water in this region makes additional favourable contributions to the hydration free energy (panels G and H). Therefore the decreased lipophilicity of **2** with respect to **17** is attributed to enhanced hydrogen bonding interactions between water and the hydrogen face of the all-*cis* tetrafluorocyclohexane ring.

## Conclusions

The all-*cis* tetrafluorocyclohexane motif has been recognised to have particularly polar properties and the ease of synthesis of the phenyl derivative **2** has prompted us to investigate it properties further as it emerges as a building block for the introduction of this new motif into medicinal chemistry and other bioactives discovery programmes. The metabolism of the phenyl cyclohexane derivatives **2**, **4–7** with varying levels of fluorination was explored in incubations with *Cunninghamella elegans*. This fungus has been used as a microbial model for mammalian metabolism. In the present study we observed that increasing the degree of fluorination of cyclohexyl ring leads to a more stable xenobiotic. The phenyl all-*cis* tetrafluorocyclohexane **2** was significantly less metabolised than the trifluoro-**4** and then difluoro-**5**, the latter of which was extensively metabolised. In the case of **2**, **4**, **6** and **7** metabolism is confined to benzylic hydroxylation.

A systematic log *P* evaluation of these ring systems shows an increase in hydrophilicity with increasing fluorination, and for the phenyl all-*cis* tetrafluorocyclohexanes (including anilines and benzoic acids) there is a maximal effect. These ring systems are at least two full log *P* units (100 fold) more hydrophilic than their non-fluorinated cyclohexane counterparts.

Molecular dynamics simulations reproduce the experimental trends and suggest that the decreased lipophilicity of **2** is due to enhanced hydrogen bonding interactions of water molecules with the hydrogen face of the cyclohexane ring with respect to bulk water. The orientation of the water near this face of the ring was consistent with the hydrogen bonding donor ability of the polarised hydrogens of the ring.

This contrasts with the energetics of water near the fluorine face of the ring which are comparable to bulk water. Altogether these studies indicate that metabolism of the all-*cis* tetrafluorocyclohexyl motif is slow, and that the ring system is significantly hydrophilic for an aliphatic motif. These factors add to the unique facially polarised aspect of this motif and make it an attractive option for inclusion in medicinal chemistry or crop protection studies.

## Conflicts of interest

There are no conflicts of interest.

## Supplementary Material

Supplementary information

Crystal structure data
