# Supplementary material for: Metabolism and hydrophilicity of the polarised ‘Janus face’ all-cis tetrafluorocyclohexyl ring, a candidate motif for drug discovery
Source: Chem Sci. 2018 Feb 19;9(11):3023–8. doi: 10.1039/c8sc00299a (PMC5916015; doi:10.1039/c8sc00299a)
Supplement: Supplementary file 1 [file SC-009-C8SC00299A-s001.pdf]

Electronic Supplementary Material (ESI) for Chemical Science.
This journal is © The Royal Society of Chemistry 2018

Supplementary Information for:

Metabolism and hydrophilicity of the polarised 'Janus face' all-cis tetrafluorocyclohexyl ring, a candidate motif for drug discovery

Andrea Rodil,[a] Stefano Bosisio,[b] Mohammed Salah Ayoup,[a] Laura Quinn,[c] David B. Cordes,[a] Alexandra M. Z. Slawin,[a] Cormac Murphy,[c] Julien Michel[b*] and David O'Hagan[a*]

do1@st-andrews.ac.uk

[a] EaStChem School of Chemistry, University of St Andrews, North Haugh, St Andrews, KY16 9ST, UK

[b] EaStChem School of Chemistry, University of Edinburgh, Joseph Black Building, David Brewster Road, Edinburgh, EH9 3FJ

[c] UCD School for Biomolecular and Biomedical Sciences, University College Dublin, Belfield, Dublin, Ireland

Contents

1. General procedures and methods 3

2. Experimental procedures for metabolism studies 4

2.1 Preparation of liquid cultures of *C. elegans* 4

2.2 Inoculation of the cultures with the corresponding xenobiotics 4

2.3 Extraction and purification of the metabolites 4

3. Estimation of lipophilicities (LogP) by reverse-phase HPLC 5

4. Preparation of new compounds to act as xenobiotics 7

4.1 Preparation of all-*cis* ((1*R*, 2*S*, 3*R*)-2,3-difluorocyclohexyl)benzene (5) 7

5. Enantiomeric excess analysis, syntheses and HPLC traces 10

5.1 Enantiomeric excess analysis for all-*cis* (2,3,6-trifluorocyclohexyl)benzene 4 . 11

5.2 Synthesis of racemic 2,3,6-trifluoro-1-phenylcyclohexan-1-ol 9 12

5.3 Enantiomeric excess analysis for 2,3,6-trifluoro-1-phenylcyclohexan-1-ol 9 17

5.4 Esterification of racemic and enantiomerically enriched all-*cis* 4-(2,3,6-trifluorocyclohexyl) benzoic acid 7 for derivatisation 18

5.5 Enantiomeric excess analysis 20

5.6 for all-*cis* methyl 4-(2,3,6-trifluorocyclohexyl) benzoate 29 20

6. Log P calculations by molecular dynamics simulations 21

7. Characterisation of fluorometabolites 23

7.1 (1*s*, 2*R*, 3*S*, 5*R*, 6*S*)-2,3,5,6-tetrafluoro-1-phenylcyclohexan-1-ol 8 23

7.2 (1*R*, 2*S*, 3*R*, 6*S*)-2,3,6-trifluoro-1-phenylcyclohexan-1-ol 9 23

7.3 (1*S*, 2*S*, 3)-2,3-difluoro-1-phenylcyclohexan-1-ol 10 23

7.4 (1*S*, 2*R*, 3*S*, 4*R*)-2,3-difluoro-4-phenylcyclohexan-1-ol 11 23

7.5 (1*S*, 3*R*, 4*S*, 5*R*)-3,4-difluoro-5-phenylcyclohexan-1-ol 12b 24

7.6 (1*R*, 3*R*, 4*S*, 5*R*)-3,4-difluoro-5-phenylcyclohexan-1-ol 12a 24

7.7 (1*S*, 3*R*, 4*R*)-4-fluoro-3-phenylcyclohexan-1-ol 13 25

7.8 4-((1*R*, 2*S*, 3*R*, 6*S*)-2,3,6-trifluoro-1-hydroxycyclohexyl)benzoic acid 15 .25

8. NMR spectra of the fluorometabolites 26

8.1 2,3,5,6-Tetrafluoro-1-phenylcyclohexan-1-ol (8) ... 26
8.2 2,3,6-Trifluoro-1-phenylcyclohexan-1-ol (9) ... 29
8.3 2,3-Difluoro-1-phenylcyclohexan-1-ol (10) ... 32
8.4 2,3-Difluoro-4-phenylcyclohexan-1-ol (11) ... 35
8.5 3,4-Difluoro-5-phenylcyclohexan-1-ol (12b) ... 38
8.6 3,4-Difluoro-5-phenylcyclohexan-1-ol (12a) ... 41
8.7 4-fluoro-3-phenylcyclohexan-1-ol (13) ... 44
8.8 4-(2,3,6-Trifluoro-1-hydroxycyclohexyl)benzoic acid (15) ... 47

9. Single crystal X-Ray structure analysis ... 50

9.1 2,3,5,6-Tetrafluoro-1-phenylcyclohexan-1-ol (8) ... 50
9.2 2,3-Difluoro-4-phenylcyclohexan-1-ol (11) ... 51
9.3 3,4-Difluoro-5-phenylcyclohexan-1-ol (12a) ... 52
9.4 3,4-Difluoro-5-phenylcyclohexan-1-ol (12b) ... 53
9.5 4-fluoro-3-phenylcyclohexan-1-ol (13) ... 54

10. References ... 55

1. General procedures and methods

All the xenobiotics used in this project were synthesised as described in the corresponding references[1] and in the following sections. All chemicals were purchased from Sigma-Aldrich or Alfa Aesar. The biological assays with the fungus *Cunninghamella elegans* were carried out in the laboratory of Dr Cormac Murphy (University College Dublin, Ireland), as reported.

Cunninghamella elegans DSM1908 was stored as a homogenate of matured sabouraud dextrose agar gels in NaCl solution (0.8% w/v) at 4 °C, and grown in commercial Sabouraud Dextrose Media (Sigma-Aldrich).

All the glassware, materials and media used for the microbial homogenate and liquid cultures were sterilised by autoclaving prior to their use. The aseptic conditions were maintained during the preparation, growth and incubation of the fungal cultures.

All the glassware used for chemical synthesis was oven-dried, cooled and used under nitrogen atmosphere, unless stated otherwise.

The progress of reactions was followed by thin-layer chromatography (TLC) using aluminium plates coated with silica gel ($60F_{245}$ Merck). TLC plates were examined under UV light at 254 and 266 nm, before being visualised with anisaldehyde-sulfuric acid or alkaline potassium permanganate. Column chromatography was performed on Merck Geduran silica gel (250-400 mesh) under a positive pressure of compressed air eluting with solvents as supplied.

Crude extracts were analysed by GCMS in an Agilent 6890 gas chromatograph coupled to a 5973 mass-selective detector. Proton (^{1}H) and proton-decoupled nuclear magnetic resonance spectra ($^{19}F\{^{1}H\}$, $^{13}C\{^{1}H\}$) were recorded on Bruker Avance III 500 or Bruker Avance III HD 500 (500 MHz ^{1}H, 476 MHz ^{19}F, 126 MHz ^{13}C) spectrometers. 2D correlation spectra (COSY, HSQC and HMBC) were also analysed for assignments of each signal. Chemical shifts (∂) are expressed in ppm, and are quoted relative to the residual solvent signal. Proton coupling constants (*J*) are given in Hz, and quoted to the nearest 0.1 Hz. Identical coupling constants are averaged.

Metabolites were isolated using a Shimadzu Prominence (SIL-20A HT autosampler, CL-20AT ternary pump, DGU-20A3R solvent degasser, SPD 20A UV detector and CVM-20A

controller module), equipped with a Phenomenex semi-preparative Kingsorb C_{18} (250 mm × 10.00 mm) 5μ column. Measurements of the lipophilicity values were conducted using a Phenomenex Luna C_{18} 100A (250 mm × 4.60 mm) 5μ column. The AcCN and water eluents used for HPLC were filtered and supplemented with 0.05% of TFA.

Enantiomeric excess was measured on a Shimadzu HPLC, consisting of a DGU-20A5 degasser, LC-20AT liquid chromatography, SIL-20AHT autosampler, CMB-20A communication bus module, SPD-M20A diode array detector and a CTO-20A column oven that allows the temperature to be set from 25-40 °C.

High-resolution mass spectrometry was acquired using electrospray ionisation (ESI), on a ThermoFisher Excalibur Orbitrap Spectrometer, operating in positive and negative mode, from solutions of the analyate in methanol or acetonitrile. Mass analyses were done at the University of St Andrews by Mrs. Caroline Horsburgh. Mass units are reported in Daltons (Da). Additional data was obtained at the EPSRC UK National Mass Spectrometry Facility at Swansea University using a MAT 95 XP spectrometer operating in chemical ionisation mode (Air-sensitive, MAT95).

X-ray crystal structures were obtained on a Rigaku XtaLAB P200 diffractometer, using multi-layer mirror monochromated Mo-Kα radiation, at the University of St Andrews by Prof. Alexandra Slawin and Dr David Cordes. Data was analysed using CrystalMaker.

2. Experimental procedures for metabolism studies

2.1 Preparation of liquid cultures of *C. elegans*

Sterile Saboraud Dextrose medium (45 mL) was inoculated with fungal homogenate (5 mL) at room temperature. The cultures were left to grow for 72 h, at 28 °C with rotary agitation (150 rpm).

2.2 Inoculation of the cultures with the corresponding xenobiotics

Xenobiotics were added dissolved in DMF solution (5 mg in 50 μL) to grown cultures (50 mL), and left to incubate for extra 72 h at 28 °C and 150 rpm.

2.3 Extraction and purification of the metabolites

After the incubation period, the supplemented liquid cultures were centrifuged using a Sorvall apparatus at 4 °C. The supernatant was separated from the fungal debris and

extracted with EtOAc (3×50 mL). The fungal cells were stored at −20 °C. The organic phase was concentrated under reduced pressure, and the solid residues redissolved in EtOAc (1mL).

An aliquot of the concentrate (100 µL) was added to MSTFA (50 µL) for trimethylsilyl derivatisation of the hydroxyl groups (1 h, 100 ºC) prior to analysis by GCMS. Further analysis of all the crude extracts was done by ^{1}H and ^{19}F NMR.

Purification of the samples was done by reverse phase HPLC. For compounds **2**, **4** and **5**, 70:30 of AcCN:water was used, while for **6** and **7** a 50:50 of AcCN:water was used. Both water and acetonitrile were supplemented with 0.05% TFA.

Analysis of the resulting metabolites and remaining starting materials was carried out by full NMR characterisation (^{1}H, ^{19}F, ^{13}C, COSY, HSQC and HMBC), and accurate mass spectrometry. X-ray structures were obtained when possible.

3. Estimation of lipophilicities (LogP) by reverse-phase HPLC

The estimation of lipophilicity values was conducted using a Phenomenex Luna C_{18} 100A (250 mm x 4.60 mm) 5µ column in a Shimadzu Prominence HPLC as previously described by the O'Hagan group.[2] A series of reference compounds from literature[3] (**Fig. S1**) were injected (5-10 µL of 0.5 mg/mL solution in AcCN).

Their retention times (R_t) were used to calculate their capacity factor (k) by the use of the following equation:

$$\text{Capacity factor (k)} = \frac{\text{Retention Time} - \text{Dead Time of the column}}{\text{Dead Time of the column}}$$

Where the dead time of the column is the time that takes for an unretained molecule (such as the solvent) to pass through it. For these particular experimental conditions, the dead time of the column is of 1.97 min.

Table S1 shows the experimental values obtained and the calculations made to develop this Log P estimation method. Literature Log P values of the reference compounds are displayed in red, along with the experimental retention times observed for each molecule (measured in triplicates to avoid and detect possible measuring errors). Average retention times were calculated using each of the three values obtained, and this average

value was used to calculate the capacity factor for each reference (using the equation described above). Finally, the logarithm of k was calculated and is displayed in green.

Table S1. Literature LogP, retention time (R_t) and capacity factors (k) for the references

Reference	Log P	Rt 1 (min)	Rt 2 (min)	Rt 3 (min)	Average Rt (min)	Capacity factor (k)	Log k
Phenol	**1.50**	3.99	3.99	3.99	3.99 ± 0.00	1.03	**0.013**
2-Fluorophenol	**1.71**	4.18	4.18	4.18	4.18 ± 0.00	1.13	**0.051**
Benzofuran	**2.67**	8.42	8.42	8.43	8.42 ± 0.01	3.29	**0.52**
Toluene	**2.73**	10.27	10.27	10.27	10.27 ± 0.00	4.23	**0.63**
o-Xylene	**3.12**	13.36	13.34	13.37	13.36 ± 0.02	5.80	**0.76**
Naphthalene	**3.35**	12.75	12.75	12.78	12.76 ± 0.02	5.50	**0.74**
Cumene	**3.66**	18.87	18.88	18.91	18.89 ± 0.02	8.61	**0.94**
t-Butylbenzene	**4.11**	23.93	23.86	23.91	23.90 ± 0.04	11.16	**1.05**
Butylbenzene	**4.26**	31.10	31.15	31.14	31.13 ± 0.03	14.84	**1.17**
Anthracene	**4.45**	25.08	25.07	25.02	25.06 ± 0.04	11.75	**1.07**
Pyrene	**4.88**	33.24	33.33	33.17	33.25 ± 0.08	15.92	**1.20**

The calculated log k (Y-axis) values were plotted against their reported LogP values (X-axis) to obtain a linear regression equation, as represented in **Plot S1**.

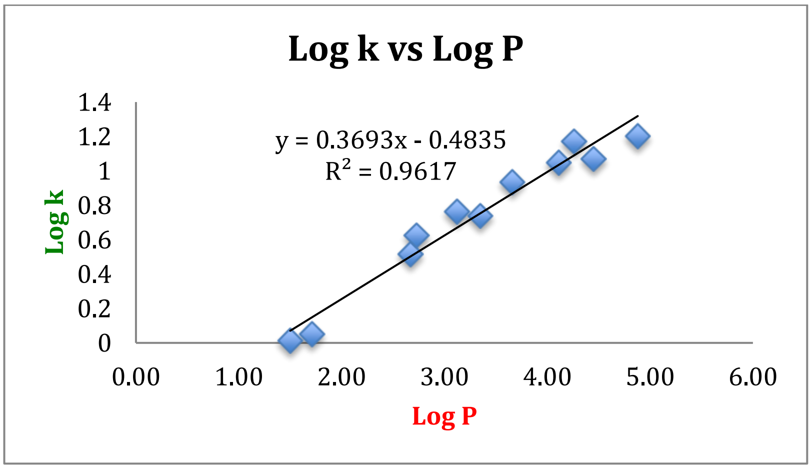

Plot S1. Regression line obtained for the reference compounds

The equation obtained in **Plot S1** was afterwards used to calculate the LogP values from a series of compounds by substitution of the logarithm of their capacity factor values in the equation above. All the retention times obtained experimentally, the calculated capacity factors and their logarithms are shown in **Table S2**, along with the LogP values (blue), estimated by substitution of the log k values in the regression line equation in **Plot S1**.

Table S2. Estimation of LogP (blue) by using the logarithm of the measured capacity factors (green)

Compound	LogP	Rt 1	Rt 2	Rt 3	Average Rt	k	Logk
**2**	**2.58**	7.78	7.78	7.72	7.76 ± 0.04	2.95	0.47
**4**	**2.64**	8.08	8.08	8.03	8.06 ± 0.03	3.10	0.49
**6**	**1.50**	4.28	4.27	4.28	4.28 ± 0.01	1.18	0.07
**7**	**1.51**	4.29	4.30	4.30	4.30 ± 0.01	1.19	0.07
**5**	**3.30**	12.68	12.68	12.61	12.66 ± 0.05	5.44	0.74
**18**	**2.79**	8.87	8.88	8.87	8.87 ± 0.01	3.52	0.55
**16**	**3.76**	17.82	17.81	17.71	17.78 ± 0.07	8.05	0.91
**17**	**4.99**	46.88	46.53	46.89	46.77 ± 0.24	22.80	1.36
**19**	**2.28**	6.45	6.45	6.45	6.45 ± 0.00	2.28	0.36
**20**	**3.23**	12.00	11.99	12.00	12.00 ± 0.01	5.11	0.71

4. Preparation of new compounds to act as xenobiotics

4.1 Preparation of all-*cis* ((1*R*, 2*S*, 3*R*)-2,3-difluorocyclohexyl)benzene (5)

All-*cis* (2,3-difluorocyclohexyl)benzene was obtained by direct fluorination of 2-fluoro-6-phenylcyclohexyl trifluoromethanesulfonate (**25**), which is a secondary product obtained in the palladium-catalysed hydrogenation of (1*R*, 2*R*, 3*R*, 6*S*)-3,6-difluoro-1,2,3,6-tetrahydro-[1,1'-biphenyl]-2-yl trifluoromethanesulfonate (**26**) described by A. J. Durie and coworkers in 2014.[1b]

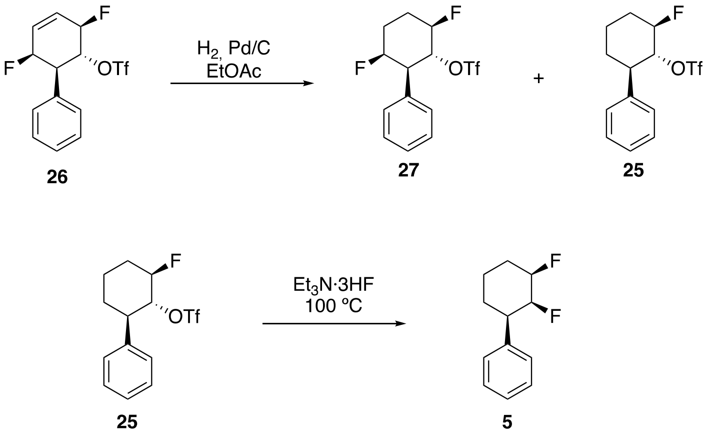

Scheme S1. Reactions leading to the formation of **5**

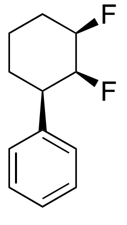

(1*R*, 2*R*, 6*R*)-2-fluoro-6-phenylcyclohexyl trifluoromethanesulfonate (**25**, 0.491 g, 2.76 mmol, 1 equiv.) was dissolved in triethylamine trihydrofluoride (Sigma-Aldrich, 98%, 3 mL, 18.4 mmol, 6.5 equiv.) and the mixture was stirred at 100 °C in a Teflon flask overnight. The reaction mixture was cooled to room temperature and quenched with saturated sodium bicarbonate (150 mL), and extracted with dichloromethane (3 × 150 mL). The combined organic phases were dried over anhydrous $MgSO_4$, filtered, and concentrated under reduced pressure. This procedure gave 0.2 g of **5** as a white crystalline solid in 37% yield. **^{1}H NMR** (500 MHz, $CDCl_3$) ∂_H 7.31 (5H, m, *H*-Ar), 4.98 (1H, ddq, *J* = 53.0, 9.7, 1.1 Hz, *H*-β), 4.58 (1H, m, *H*-γ), 2.66 (1H, ddd, *J* = 35.4, 13.1, 4.1 Hz, *H*-α), 1.96 (4H, m, *H*-δ, *H*-ζ_{ax}, *H*-ε_{eq}), 1.70 (1H, m, *H*-ε_{ax}), 1.45 (1H, m, *H*-ζ_{eq}) **^{19}F{^{1}H} NMR** (470 MHz, $CDCl_3$) δ_F -213.8 (F, d, *J* = 16.6 Hz), -181.0 (F, d, *J* = 16.6 Hz) **^{13}C NMR** (126 MHz, $CDCl_3$) ∂_C 141.3 (*C*-Ar), 128.5 (*C*-Ar), 127.9 (*C*-Ar), 127.0 (*C*-Ar), 92.8-90.7 (*C*-β, *C*-γ), 46.0 (*C*-α, dd, *J* = 19.3, 5.6 Hz), 25.7 (*C*-δ, dd, *J* = 18.6, 3.3 Hz), 25.2 (*C*-ε, d, *J* = 3.3 Hz), 22.6 (*C*-ζ, d, *J* = 11.9 Hz) **HRMS** (ESI$^+$) *m/z* calc. for $C_{12}H_{14}F_2Na$ $[M+Na]^+$ 219.0961, found 219.0957.

^{1}H NMR

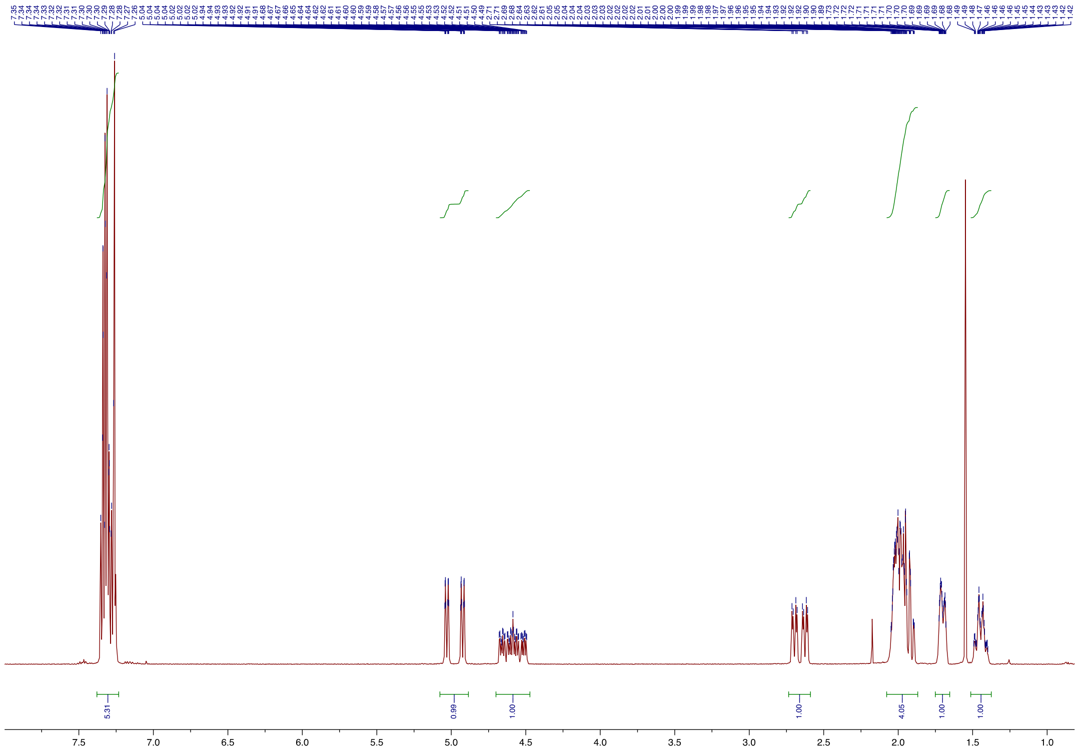

^{13}C NMR

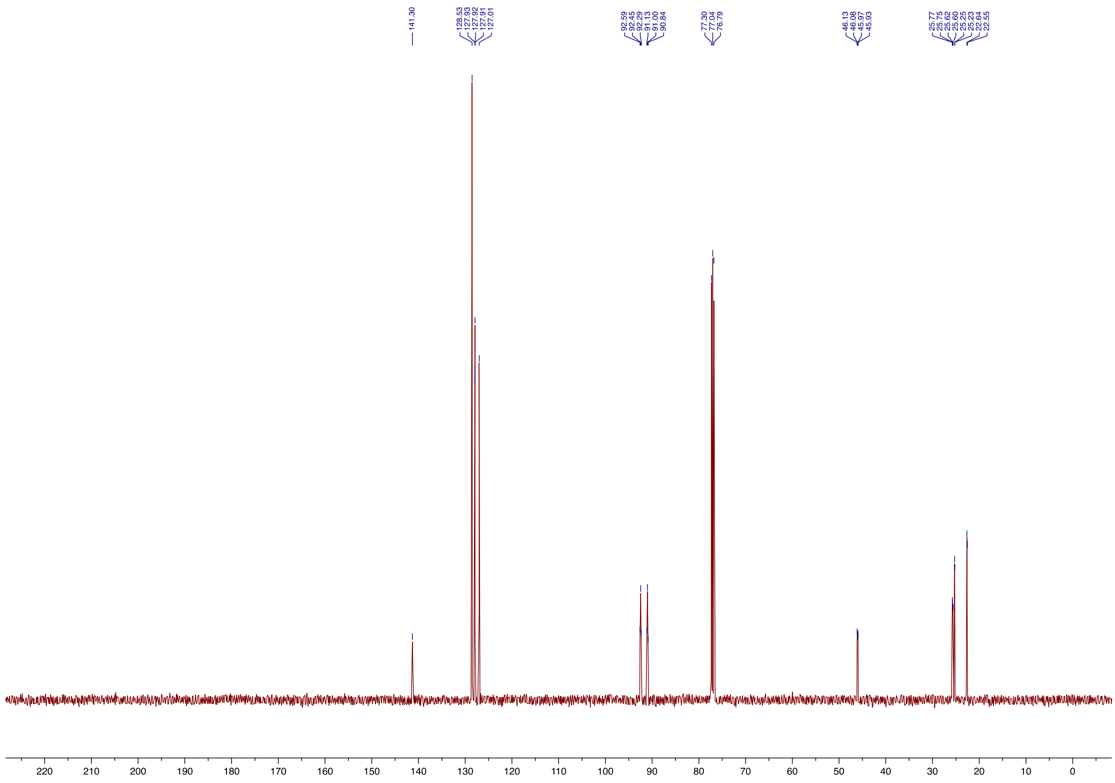

^{19}F NMR

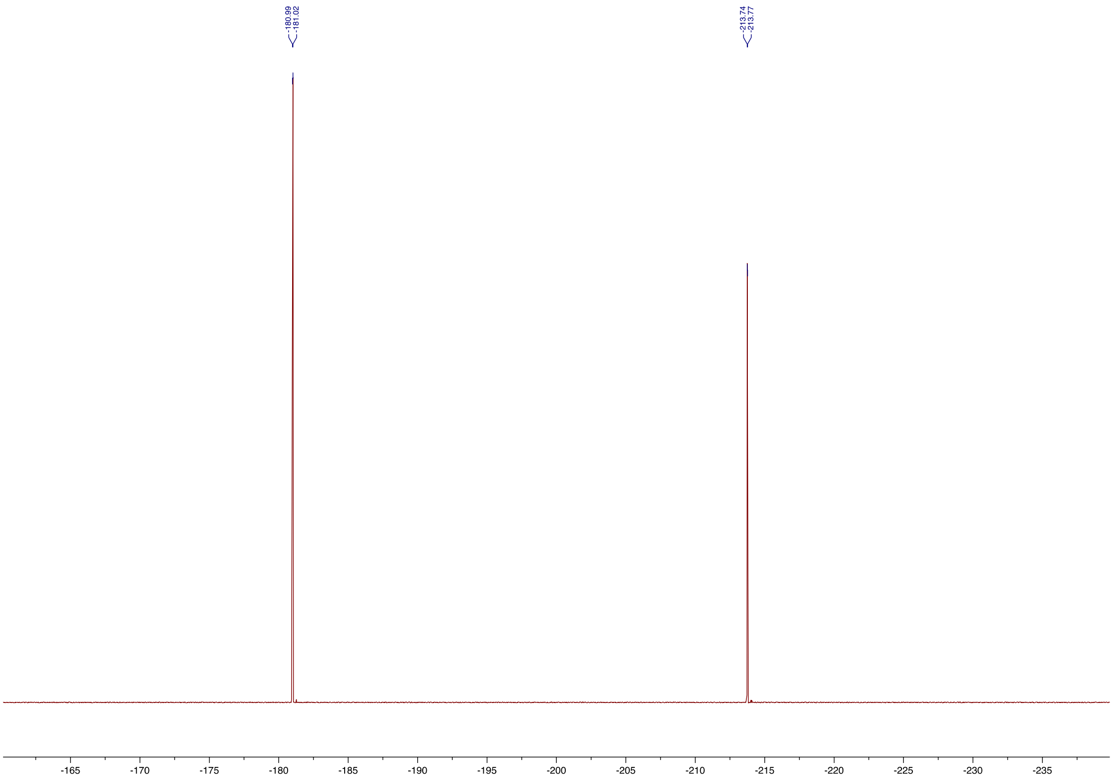

5. Enantiomeric excess analysis, syntheses and HPLC traces

Enantiomeric excess was calculated for two of the chiral substrates, in order to check if *Cunninghamella elegans* works stereospecifically. Given the complexity of difluoro compound's **5** metabolism, it was decided to start the investigations on the trifluorinated derivatives **4** and **6**.

The chiral HPLC analysis requires the injection of the racemic products prior to the hypothetically enantiomerically enriched ones. Derivatisation of the carboxylic acids into esters was also necessary prior to the injection in the HPLC.

For practical reasons, it was decided to start injecting the starting materials. This was due to their immediate availability, and as an exploration for any enantiomerical richness after the incubation.

5.1 Enantiomeric excess analysis for all-*cis* (2,3,6-trifluorocyclohexyl)benzene 4

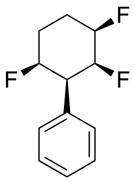

HPLC data for compound **4**: Chiralcel IC (95:5 hexane:IPA, flow rate 1 mLmin^{-1}, 211 nm, 30 °C), t_R (A): 6.2 min, t_R (B): 7.0 min. 57:43 ee.

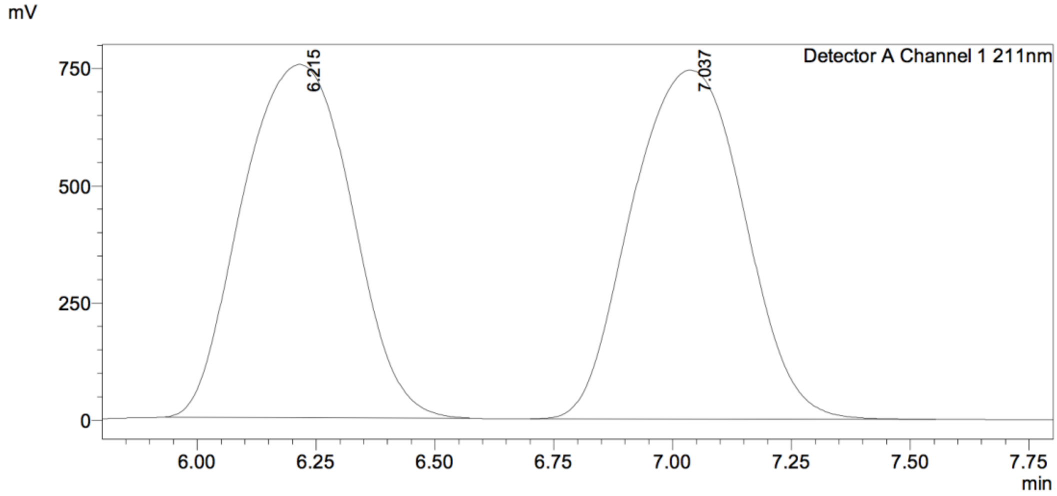

<Peak Table>

Detector A Channel 1 211nm

Peak#	Ret. Time	Area%
1	6.215	49.549
2	7.037	50.451
Total		100.000

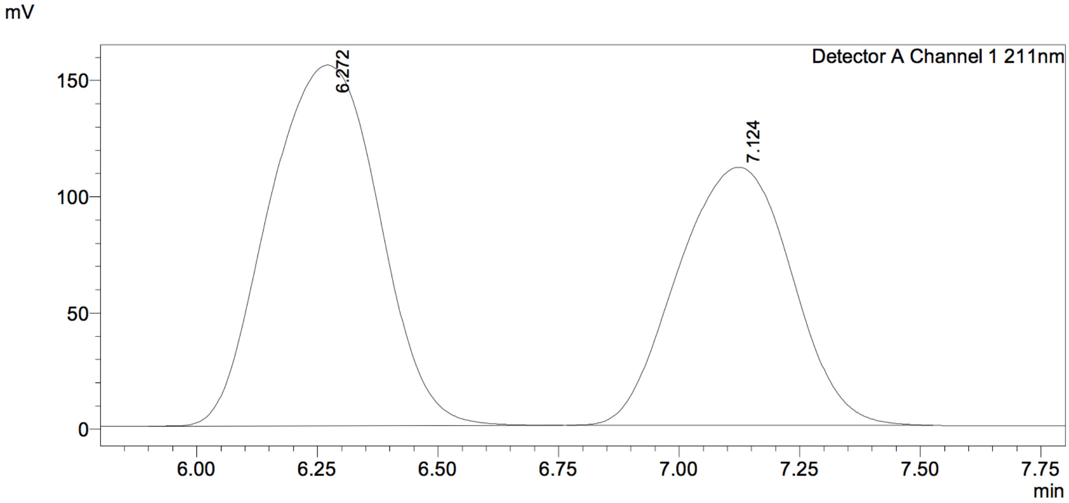

<Peak Table>

Detector A Channel 1 211nm

Peak#	Ret. Time	Area%
1	6.272	57.487
2	7.124	42.513
Total		100.000

5.2 Synthesis of racemic 2,3,6-trifluoro-1-phenylcyclohexan-1-ol 9

Synthesis of racemic 2,3,6-trifluoro-1-phenylcyclohexan-1-ol **9** was necessary to compare with the enantiomerically enriched product obtained after the transformation. **Scheme SX** shows the followed procedure.

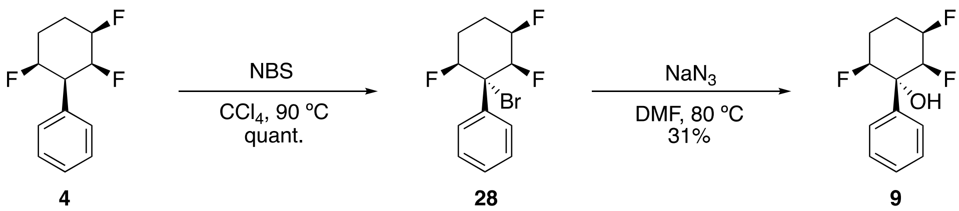

Scheme S2. Synthetic route to racemic 2,3,6-trifluoro-1-phenylcyclohexan-1-ol **9**

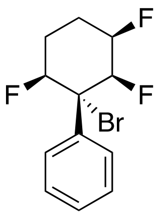

Racemic all-*cis* (2,3,6-trifluorocyclohexyl)benzene (**4**, 0.200 g, 0.93 mmol, 1 equiv.) was dissolved in CCl_4 (2 mL). NBS (210 mg, 1.12 mmol, 1.5 equiv.) was added to the solution. CH_3CN (0.1 mL) was added for better solubilisation of **4**. The reaction mixture was heated at 90 °C for 48 h. The mixture was concentrated under pressure and redissolved in water (10 mL). The aqueous phase was extracted with Et_2O (3 x 10 mL). The combined organic phases were dried over Na_2SO_4 anhydrous, filtered and concentrated under reduced pressure. This procedure gave product **28** in quantitative yield and no further purification was needed. **^{1}H NMR** (500 MHz, $CDCl_3$) ∂_H 7.68 (2H, dq, J = 8.3, 1.2 Hz), 7.44 (2H, m), 7.37 (1H, m), 5.74 (1H, dddt, J = 49.3, 8.3, 2.6, 1.2 Hz), 5.5 (2H, m), 2.46 (3H, m), 2.01 (1H, dq, J = 8.3, 5.9, 4.2 Hz) **^{19}F{^{1}H} NMR** (470 MHz, $CDCl_3$) δ_F -189.75 (q, J = 14.7, 18.5 Hz), -188.84 (d, J = 14.7 Hz), -171.75 (d, J = 18.5 Hz) **^{13}C NMR** (126 MHz, $CDCl_3$) ∂_C 138.01, 129.03 (d, J = 15.5 Hz), 126.34 , 90.37 – 86.46 (m), 24.98 (dd, J = 22.4, 12.1 Hz), 20.10 (dd, J = 20.4, 4.0 Hz) **HRMS** (ESI$^+$) *m/z* calc. for $C_{12}H_{12}BrF_3Na$ $[M+Na]^+$ 314.9972, found 314.9956.

^{1}H NMR

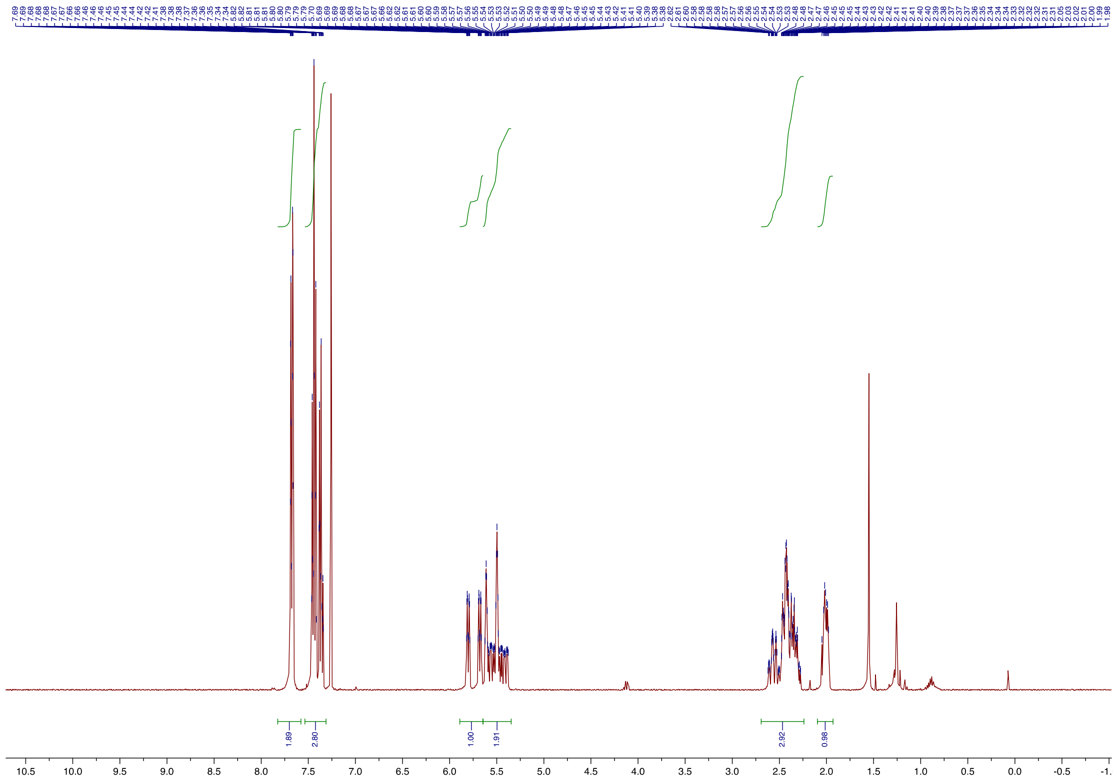

$^{19}F\{^{1}H\}$ NMR

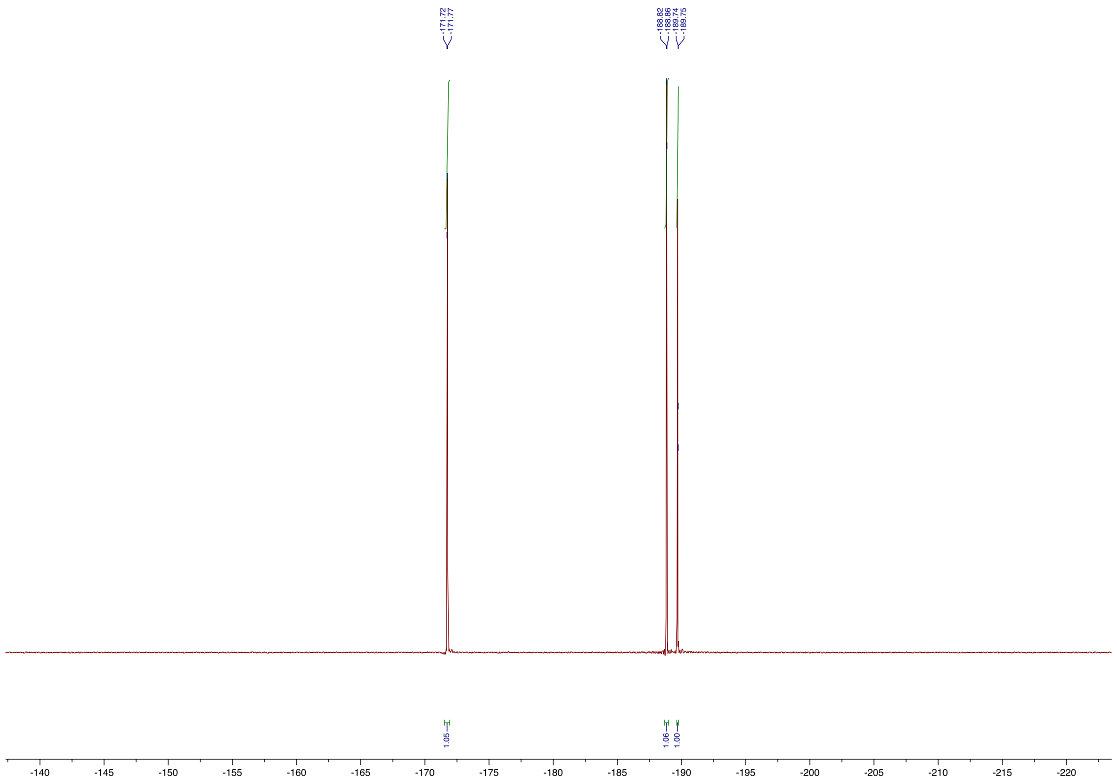

^{13}C NMR

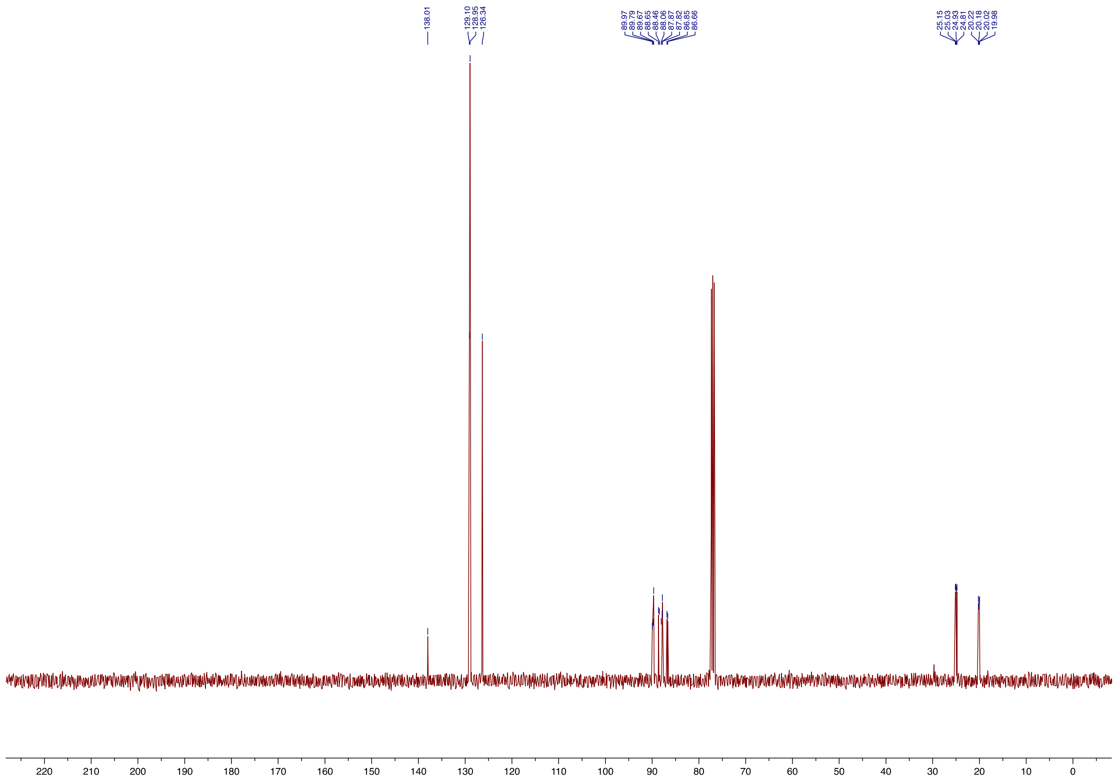
138.01
129.10
128.95
126.34
89.97
89.79
89.67
88.65
88.46
88.06
87.87
87.82
86.85
86.66
25.15
25.03
24.93
24.81
20.22
20.18
20.02
19.98
220 210 200 190 180 170 160 150 140 130 120 110 100 90 80 70 60 50 40 30 20 10 0

HMBC

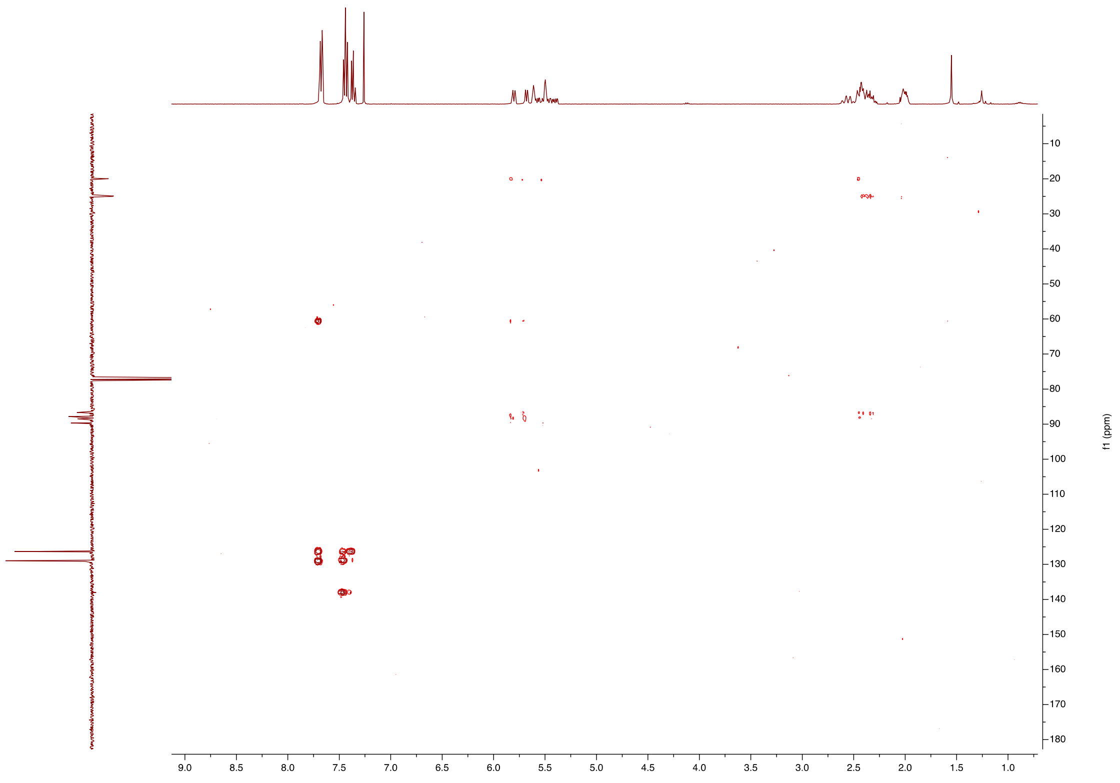
9.0 8.5 8.0 7.5 7.0 6.5 6.0 5.5 5.0 4.5 4.0 3.5 3.0 2.5 2.0 1.5 1.0
10 20 30 40 50 60 70 80 90 100 110 120 130 140 150 160 170 180
f1 (ppm)

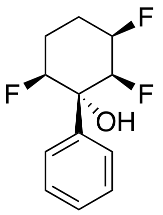

(1-Bromo-2,3,6-trifluorocyclohexyl)benzene (**28**, 40 mg, 0.14 mmol, 1 equiv.) was dissolved in DMF (2 mL). NaN_3 (25 mg, 0.30 mmol, 2 equiv.) was added and the mixture was heated to 80 °C for 18 h. The reaction was cooled to r. t. and quenched with water (50 mL). The aqueous phase was extracted with Et_2O (3 x 50 mL). The combined organic phases were washed with brine (3 x 50 mL), dried over Na_2SO_4 anhydrous, filtered and concentrated under reduced pressure. Further purification was carried out by column chromatography (100% petroleum ether), yielding hydroxylated **9** as a yellow oily product in 31% yield. **^{1}H NMR** (500 MHz, $CDCl_3$) ∂_H 7.72 (2H, dd, *J* = 7.6, 1.7 Hz), 7.46 (2H, dd, *J* = 8.4, 6.6 Hz), 7.40 (1H, m), 5.17 (2H, m), 4.90 (dd, *J* = 45.8, 3.2 Hz, 1H), 2.22 (3H, m), 1.95 (1H, m) **^{19}F{^{1}H} NMR** (470 MHz, $CDCl_3$) δ_F -189.10 (F, d, *J* = 23.0 Hz), -191.58 (F, m), -207.7 (F, m) **^{13}C NMR** (126 MHz, $CDCl_3$) ∂_C 140.82 , 127.3 (m), 90.02 (m), 73.77, 24.73 (dd, *J* = 21.8, 12.3 Hz), 19.96 (dd, *J* = 20.3, 4.4 Hz) **HMRS** (ESI$^+$) *m/z* calc. for $C_{12}H_{13}OF_3Na$ [M+Na]$^+$ 253.0816, found 253.0812.

^{1}H NMR

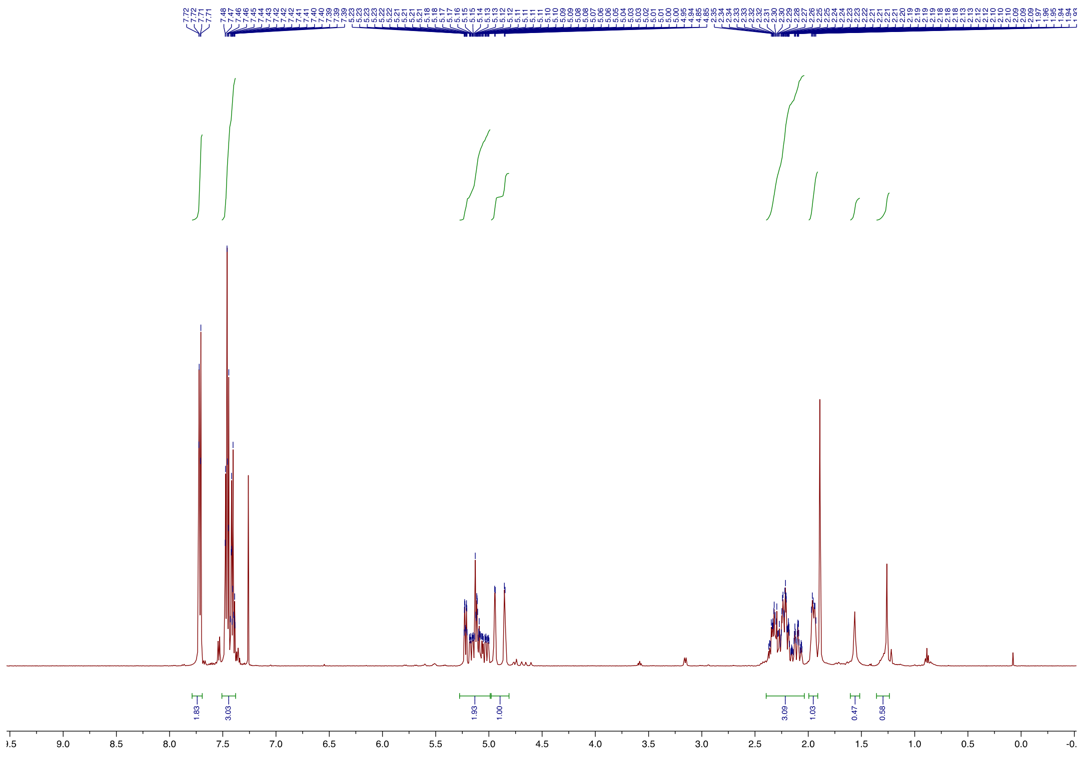

$^{19}F\{^1H\}$ NMR

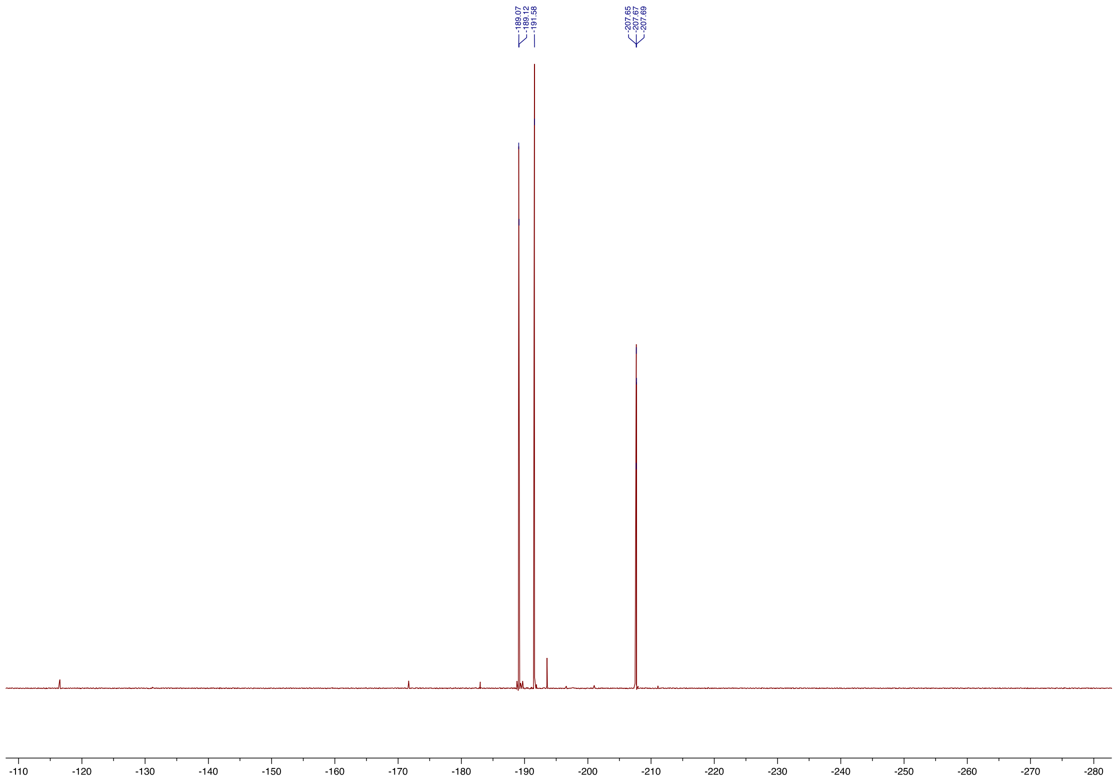

^{13}C NMR

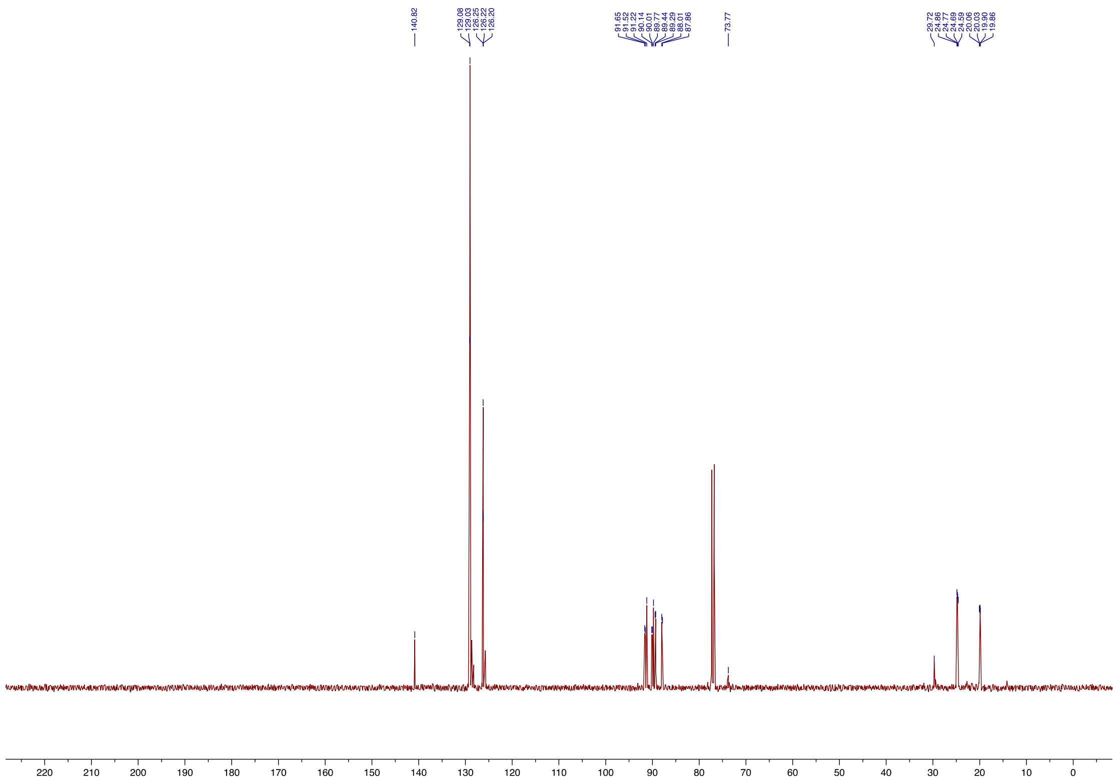

5.3 Enantiomeric excess analysis for 2,3,6-trifluoro-1-phenylcyclohexan-1-ol 9

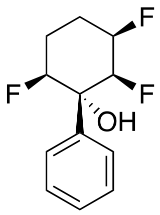

HPLC data for compound **9**: Chiralcel ID (95:5 hexane:IPA, flow rate 1 mLmin^{-1}, 211 nm, 30 °C), t_R (A): 13.5 min, t_R (B): 31.0 min; 38:62 ee.

<Chromatogram>

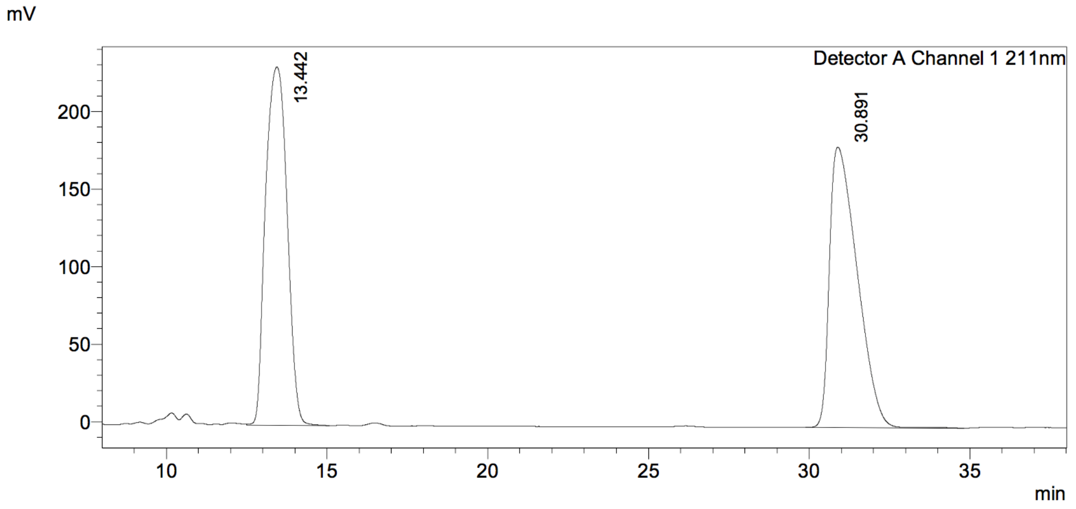

Detector A Channel 1 211nm

Peak#	Ret. Time	Area%
1	13.442	49.911
2	30.891	50.089
Total		100.000

<Chromatogram>

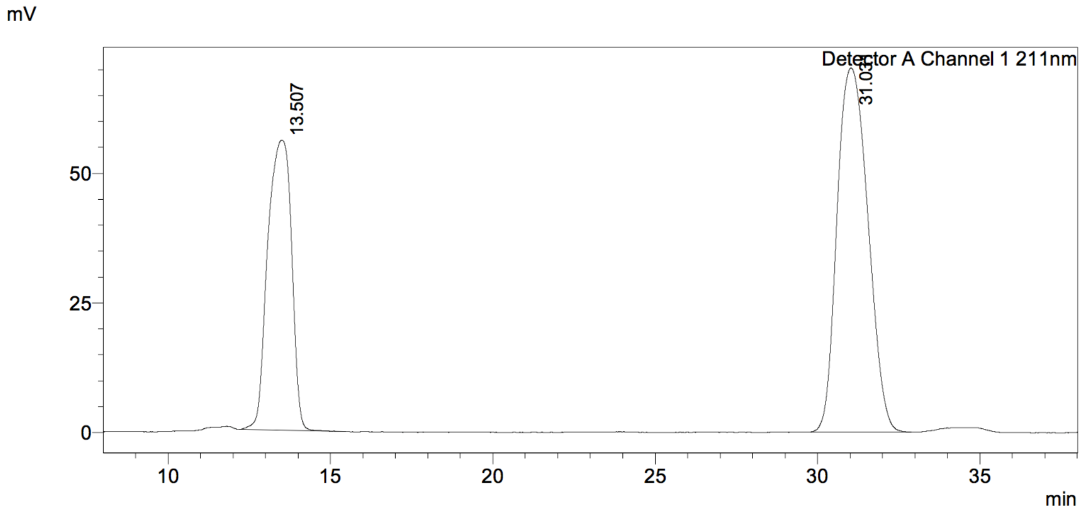

Detector A Channel 1 211nm

Peak#	Ret. Time	Area%
1	13.507	38.116
2	31.031	61.884
Total		100.000

5.4 Esterification of racemic and enantiomerically enriched all-*cis* 4-(2,3,6-trifluorocyclohexyl) benzoic acid 7 for derivatisation[4]

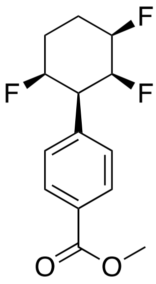

4-(2,3,6-trifluorocyclohexyl)benzoic acid (5 mg, 1 equiv.) was dissolved in a mixture CH_3CN (200 µL) and methanol (150 µL). H_2SO_4 (conc., 50 µL) was added dropwise. The mixture was stirred at 80 °C for 16 h. The reaction mixture was cooled and quenched with Na_2CO_3 (20% sol., added dropwise until pH 7.0). The neutral solution was extracted with DCM (3 x 1 mL). The combined organic phases were washed with water (3 x 3 mL), dried over anhydrous Na_2SO_4, filtered and under reduced pressure, resulting in methyl ester **29** in quantitative yield. No further purification was required. The enantiomerically enriched **7** recovered from the incubation with *C. elegans* was esterified following the same procedure, and the characterisation matches the racemic data. **^{1}H NMR** (500 MHz, $CDCl_3$) ∂_H 8.03 (d, *J* = 8.4 Hz, 1H), 7.60 – 7.52 (m, 1H), 5.10 (dd, *J* = 51.6, 8.6 Hz, 1H), 4.88 (dt, *J* = 47.7, 2.9 Hz, 1H), 4.74 – 4.54 (m, 1H), 3.92 (s, 2H), 2.76 (t, *J* = 37.4 Hz, 1H), 2.51 – 2.29 (m, 1H), 2.00 (dt, *J* = 13.2, 4.0 Hz, 1H), 1.83 – 1.62 (m, 1H) **^{19}F{^{1}H} NMR** (470 MHz, $CDCl_3$) δ_F -183.01, -190.97, -209.50 (dd, *J* = 26.8, 13.2 Hz) **^{13}C NMR** (126 MHz, $CDCl_3$) ∂_C 166.87 , 142.38 , 129.59 (d, *J* = 70.0 Hz), 92.36 – 86.86 (m), 52.19 , 48.35 (d, *J* = 5.4 Hz), 29.72 , 28.50 (dd, *J* = 22.5, 12.1 Hz), 20.36 (dd, *J* = 20.4, 3.6 Hz) **HMRS** (ESI$^+$) *m/z* calc. for $C_{14}H_{16}O_2F_3$ $[M+H]^+$ 273.1099, found 273.1098

^{1}H NMR

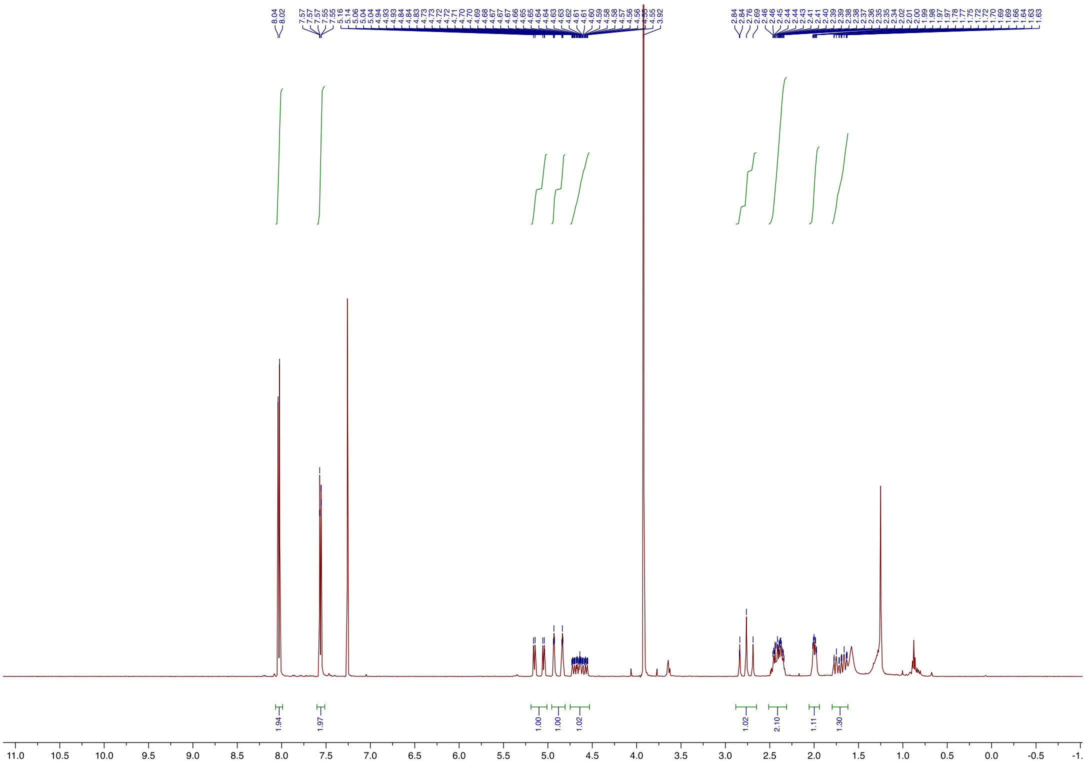

^{19}F{^{1}H} NMR

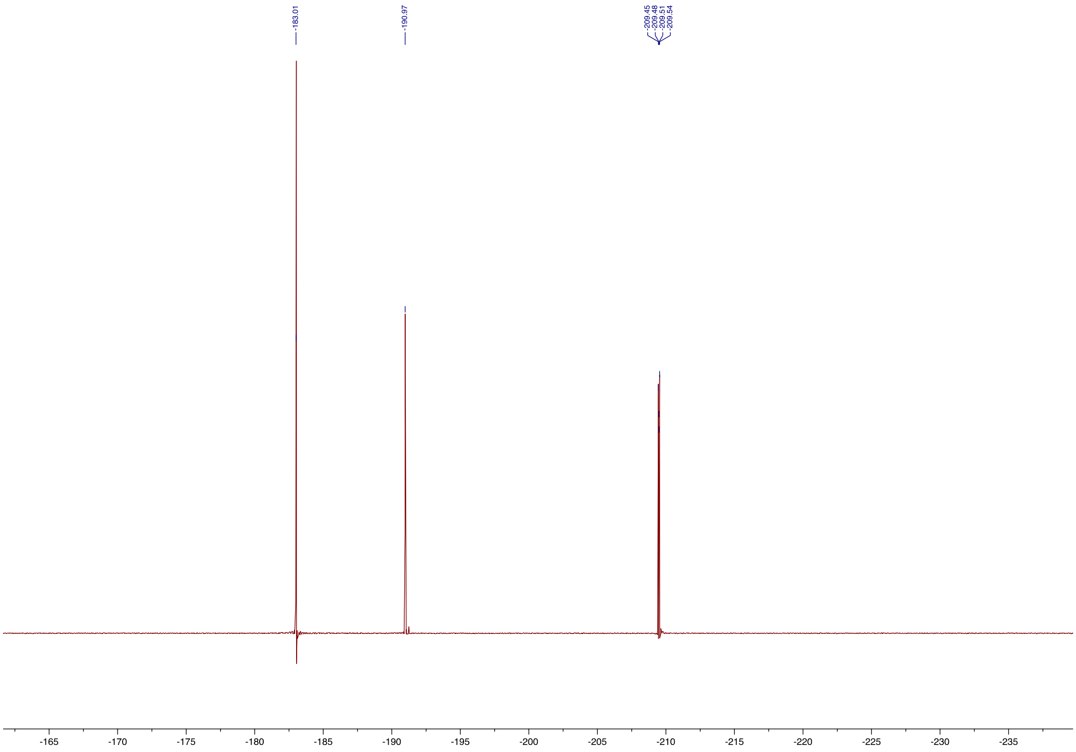

^{13}C NMR

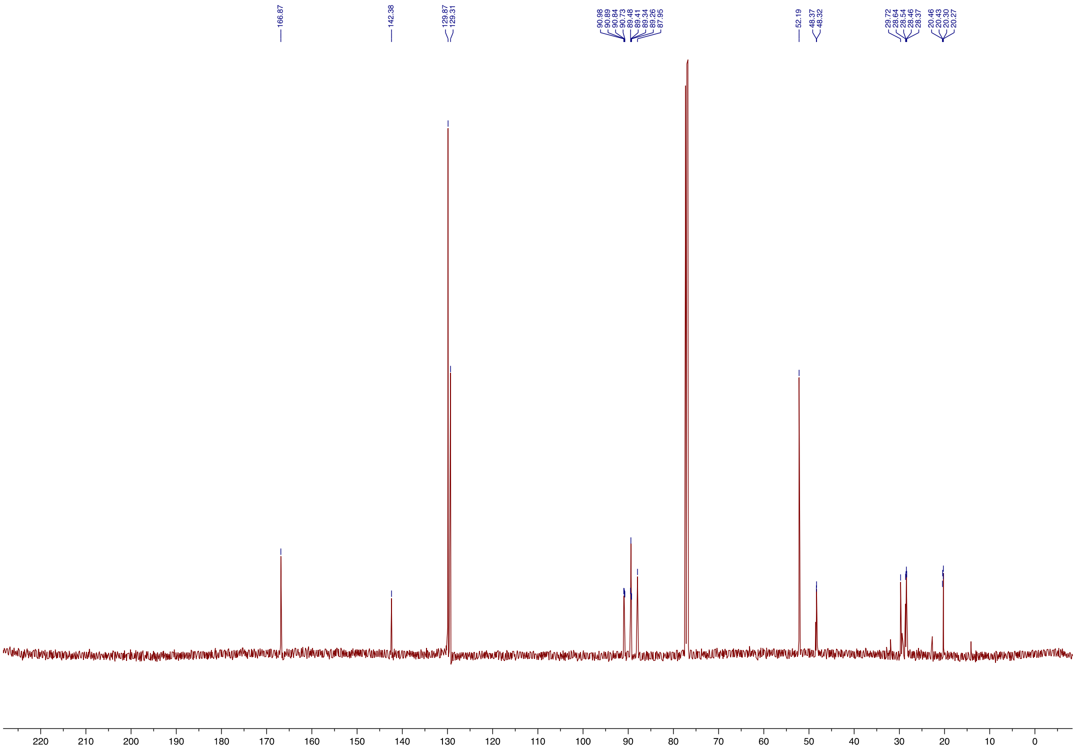

5.5 Enantiomeric excess analysis

5.6 for all-*cis* methyl 4-(2,3,6-trifluorocyclohexyl) benzoate 29

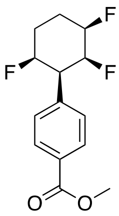

HPLC data for compound **29**: Chiralcel IC (95:5 hexane:IPA, flow rate 1 mLmin^{-1}, 211 nm, 30 °C), t_R (A): 24.1 min, t_R (B): 28.1 min; 48:52 ee.

<Chromatogram>

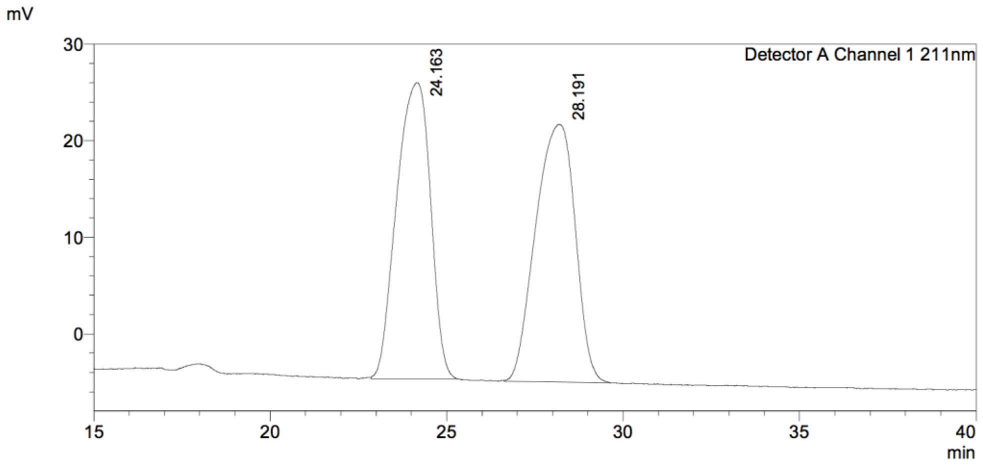

Detector A Channel 1 211nm

Peak#	Ret. Time	Area%
1	24.163	49.593
2	28.191	50.407
Total		100.000

<Chromatogram>

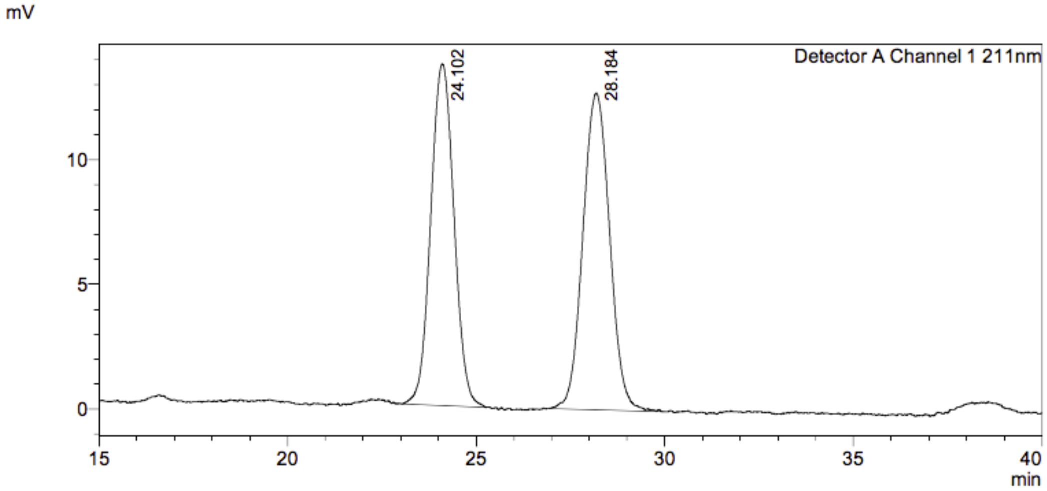

Detector A Channel 1 211nm

Peak#	Ret. Time	Area%
1	24.102	48.397
2	28.184	51.603
Total		100.000

6. Log P calculations by molecular dynamics simulations

Starting from SMILE string, molecules **2**, **4**, **5**, **16** and **17** were converted into pdb files with obabel [5] Substituents were examined and correctly placed, based on the stereochemistry, modifying pdb files with Maestro (v.10.1.012, rel 2015-1, Schrödinger).[6] Thus, molecules were parametrized with GAFF force field and AM1-BCC charges with Antechamber module of AmberTools16.[7]

Sequentially, employing *tleap* package, each compound was solvated in GAFF cyclohexane and TIP3P in a cubic box with a minimum distance between solute and box set to 20.0 Å.[8] Then, systems were minimized with 100 steps of steepest descent algorithm and equilibrated. Firstly, the water molecules were equilibrated in a NVT ensemble for 200 ps at 298 K, followed by an NPT simulation for further 200 ps at 1 atm with AmberTools module Sander. Finally, a molecular dynamics simulation in NPT ensemble was run, to reach the final desired density (0.7 g cm^{-3} for cyclohexane and 1 g cm^{-3} for water).

Thus, the partition coefficient, *log P,* for each molecule was computed as:

$$\log P = -\frac{1}{2.303\, k_B T}(\Delta G_{org} - \Delta G_{aq})$$

where k_B is the Boltzmann constant, T the temperature, □G_{org} the solvation free energy in cyclohexane and □G_{aq} the solvation free energy in water. To estimate the solvation free energy in water and cyclohexane, alchemical free energy calculations were run according to the double annihilation technique.[9]

Initially, molecules' partial charges are turned off both in water, in vacuum and in cyclohexane, discharging step, giving an excess discharging free energy change. Secondly, molecules interactions are fully decoupled by switching off the van der Waals terms, vanishing step, computing a vanishing free energy change. Both discharging and vanishing steps were subdivided in 11 intermediate simulations, by coupling molecules' charges and van der Waals values with a parameter □□∈ [0,1].

Each □ window was run for 2 ns, with a velocity-Verlet integrator algorithm, whose time step was 2 fs, and constraints applied to all molecules' bonds. Simulations were performed in an NPT ensemble and temperature control was achieved with an Andersen thermostat with a coupling constant of 10 ps^{-1}. Pressure control was maintained by a Monte Carlo barostat, with isotropic scaling every 100 fs. Periodic boundary conditions were imposed with a 12 Å cutoff for the non-bonded interactions, using a shifted atom-based Barker Watter reaction field, with dielectric constant of 82.0 for TIP3P water and 1.0 for GAFF cyclohexane.

The final excess free energy changes were estimated with multistate Bennet's acceptance ratio (MBAR) and they were corrected for the long range dispersion interactions.[10]

Simulations were repeated twice, starting from different initial velocities assignments. In this way, the final solvation free energy was computed as an average of both runs and the statistical uncertainties, σ_{err}, were estimated as:

$$\sigma_{err} = \frac{\sigma}{\sqrt{2}}$$

where □ is the standard deviation of both free energy of solvation. The same statistical procedure was followed to estimate the average *log P* value and its statistical uncertainty. The calculated quantities are given in Table S3.

Compound	□$G_{org} \pm \sigma_{err}$ (kcal mol^{-1})	□$G_{aq} \pm \sigma_{err}$ (kcal mol^{-1})	$\log P_{pred} \pm \sigma_{err}$
**17**	-8.59 ± 0.14	-4.22 ± 0.07	3.20 ± 0.16
**16**	-6.79 ± 0.13	-2.49 ± 0.08	3.15 ± 0.04
**5**	-7.83 ± 0.08	-2.69 ± 0.12	3.77 ± 0.05
**4**	-8.20 ± 0.04	-4.27 ± 0.13	2.88 ± 0.12
**2**	-7.90 ± 0.23	-5.15 ± 0.27	2.01 ± 0.03

Table S3: Calculated solvation free energy in organic (□G_{org}) and aqueous (□G_{aq}) solutions, calculated log Ps ($\log P_{pred}$) along with their standard error (σ_{err}) for compounds **17, 16, 5, 4** and **2.**

Grid-cell theory analyses

50 ns MD simulations of 2 and 17 explicitly solvated with TIP3P water molecules were carried out with the software SOMD. A time-step of 2 fs was used, all bonds were constrained, and the simulations were carried out at 25 degrees Celsius and 1 atm. Snapshots were saved every 100 fs for post-processing. Periodic boundary conditions were imposed with a 10 Å cutoff for the non-bonded interactions, using a shifted atom-based Barker Watter reaction field, with dielectric constant of 78.3. Solute atoms were restrained to their initial conformation with Cartesian positional harmonic restraints of 10 kcal mol^{-1}Å^{-2}. The initial solutes conformation was constructed from a representative structure sampled during the preceding alchemical free energy calculations.

The resulting MD trajectories were analysed with the software Nautilus. A cubic grid of 24 Å edges length and 0.5 Å spacing was centred on the solutes. Grid cell theory analyses as described by Michel *et al.*[11] were carried out for water molecules present in the cubic grid regions.

7. Characterisation of fluorometabolites

7.1 (1*s*, 2*R*, 3*S*, 5*R*, 6*S*)-2,3,5,6-tetrafluoro-1-phenylcyclohexan-1-ol 8

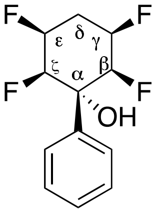

^{1}H NMR (500 MHz, $CDCl_3$) ∂_H 7.71 (2H, dd, *J* = 7.5, 1.8 Hz, *H*-Ar), 7.47 (3H, m, *H*-Ar), 5.10 (4H, m, *H*-β, *H*-γ, *H*-ε, *H*-ζ), 2.87 (1H, m, *H*-δ_{eq}), 2.49 (1H, m, *H*-δ_{ax}), 2.01 (1H, s, -O*H*) **^{19}F{^{1}H} NMR** (470 MHz, $CDCl_3$) δ_F -207.7 (2F, dd, *J* = 7.6, 5.1 Hz), -198.8 (2F, dd, *J* = 7.6, 5.0 Hz) **^{13}C NMR** (126 MHz, $CDCl_3$) ∂_C 138.7 (*C*-Ar, visible in HMBC), 129.7 (*C*-Ar), 129.3 (*C*-Ar), 126.1 (*C*-Ar), 90.5-85.2(*C*-β, *C*-γ, *C*-ε, *C*-ζ), 73.2 (*C*-α, visible in HMBC), 26.8 (*C*-δ) **HRMS** (ESI^-) *m/z* calc. for $C_{12}H_{11}OF_4$ $[M{-}H]^-$ 247.0746, found 247.0750.

7.2 (1*R*, 2*S*, 3*R*, 6*S*)-2,3,6-trifluoro-1-phenylcyclohexan-1-ol 9

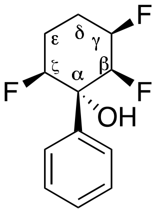

^{1}H NMR (500 MHz, $CDCl_3$) ∂_H 7.72 (2H, dd, *J* = 7.8, 1.6 Hz, *H*-Ar), 7.44 (3H, m, *H*-Ar), 5.15 (2H, m, *H*-γ, *H*-ζ), 4.90 (1H, d, *J* = 45.6 Hz, *H*-β), 2.32 (1H, m, *H*-δ_{ax}), 2.22 (1H, m, *H*-ε_{ax}), 2.11 (1H, m, *H*-ε_{eq}), 1.96 (1H, m, *H*-δ_{eq}), 1.87 (1H, s, -O*H*) **^{19}F{^{1}H} NMR** (470 MHz, $CDCl_3$) δ_F -207.7 (*F*, dd, *J* = 24.3, 13.5 Hz), -191.6 (*F*, d, *J* = 13.5 Hz), -189.1 (*F*, d, *J* = 24.3 Hz) **^{13}C NMR** (126 MHz, $CDCl_3$) ∂_C 140.8(*C*-Ar), 129.1 (*C*-Ar), 126.2 (*C*-Ar), 90.8 (*C*-ζ, dd, *J*= 200.7, 12.2 Hz), 90.5 (*C*-β, d, *J*= 183.3 Hz), 88.6 (*C*-γ, dd, *J*= 200.7, 21.6 Hz), 73.8 (*C*-α, visible in HMBC), 24.7 (*C*-ε, dd, *J*= 21.6, 12.2 Hz), 19.9 (*C*-δ, d, *J*= 20.5 Hz) **HRMS** (ESI^+) *m/z* calc. for $C_{12}H_{13}OF_3Na^+$ $[M{+}Na]^+$ 253.0816, found 253.0807.

7.3 (1*S*, 2*S*, 3)-2,3-difluoro-1-phenylcyclohexan-1-ol 10

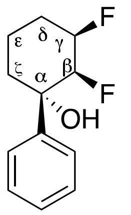

^{1}H NMR (500 MHz, $CDCl_3$) ∂_H 7.56 (2H, m, *H*-Ar), 7.41 (2H, m, *H*-Ar), 7.35 (1H, m, *H*-Ar), 5.05 (1H, m, *H*-γ) 4.78 (1H, dd, *J* = 50.7, 8.9 Hz, *H*-β), 2.26 (1H, m, *H*-ζ), 2.03 (1H, m, *H*-δ_{eq}), 1.89 (1H, m, *H*-δ_{ax}), 1.82 (2H, m, *H*-ε), 1.75 (1H, d, *J* = 15.1 Hz, *H*-ζ) **^{19}F{^{1}H} NMR** (470 MHz, $CDCl_3$) δ_F -205.3 (*F*, d, *J* = 16.4 Hz), -189.8 (*F*, d, *J* = 16.3 Hz) **^{13}C NMR** (126 MHz, $CDCl_3$) ∂_C 144.2 (*C*-Ar, visible in HMBC), 129.0 (*C*-Ar), 128.2 (*C*-Ar), 125.5 (*C*-Ar), 91.7 (*C*-β, dd, *J*= 198.4, 15.9 Hz), 89.7 (*C*-γ, dd, *J*= 196.2, 18.1 Hz), 74.7 (*C*-α, visible in HMBC), 31.2 (*C*-ζ), 25.1 (*C*-δ, dd, *J*= 21.9, 3.1 Hz), 17.9 (*C*-ε, d, *J*= 11.9 Hz) **HRMS** (ESI^+) *m/z* calc. for $C_{12}H_{18}ONF_2$ $[M{+}NH_4]^+$ 230.1351, found 230.1355.

7.4 (1*S*, 2*R*, 3*S*, 4*R*)-2,3-difluoro-4-phenylcyclohexan-1-ol 11

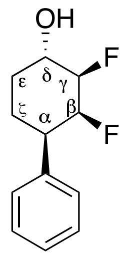

^{1}H NMR (500 MHz, $CDCl_3$) ∂_H 7.35 (5H, m, *H*-Ar), 5.04 (1H, dd, *J* = 60.8, 9.1 Hz, *H*-β) 4.41 (1H, dddd, *J* = 47.7, 26.8, 9.3, 2.3 Hz, *H*-γ), 4.25 (1H, m, *H*-δ), 2.77 (1H, ddd, *J* = 35.7, 13.2, 4.2 Hz, *H*-α), 2.22 (1H, m, *H*-ε_{ax}), 2.10 (1H, m, *H*-ζ_{ax}), 1.82 (1H, dddd, *J* = 14.0, 6.1, 3.0, 1.6 Hz, *H*-ζ_{eq}) 1.54 (1H, qd, *J* = 13.2, 4.2 Hz, *H*-ε_{eq}) **^{19}F{^{1}H} NMR** (470 MHz, $CDCl_3$) δ_F -210.6 (*F*, d, *J* = 17.0 Hz), -195.36 (*F*, d, *J* = 17.4 Hz) **^{13}C NMR** (126 MHz, $CDCl_3$) ∂_C 140.2 (*C*-Ar, visible in HMBC), 128.6 (*C*-Ar), 127.7 (*C*-Ar), 127.3 (*C*-Ar), 95.8 (*C*-γ, dd, *J* = 184.6, 18.2 Hz), 92.4 (*C*-β, dd, *J* = 181.8, 16.3 Hz), 68.7 (*C*-δ, dd, *J* = 18.8, 3.0 Hz), 45.68 (*C*-α, dd, *J* = 19.2, 5.2 Hz), 30.45 (*C*-ε, d, *J* = 7.7 Hz), 24.24 (*C*-ζ, d, *J* = 4.4 Hz) **HRMS** (ESI$^+$) *m/z* calc. for $C_{12}H_{14}OF_2Na$ $[M+Na]^+$ 235.0910, found 235.0903.

7.5 (1*S*, 3*R*, 4*S*, 5*R*)-3,4-difluoro-5-phenylcyclohexan-1-ol 12b

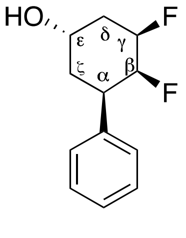

^{1}H NMR (500 MHz, $CDCl_3$) ∂_H 7.32 (5H, m, *H*-Ar), 5.00 (2H, m, *H*-β, *H*-γ) 4.46 (1H, s, *H*-ε), 3.25 (1H, ddd, *J* = 36.2, 13.4, 4.2 Hz, *H*-α), 2.19 (3H, m, *H*-δ, *H*-ζ_{ax}), 1.82 (1H, d, *J* = 14.2 Hz, *H*-ζ_{eq}) **^{19}F{^{1}H} NMR** (470 MHz, $CDCl_3$) δ_F -217.5 (*F*, d, *J* = 16.0 Hz), -193.9 (*F*, d, *J* = 16.0 Hz) **^{13}C NMR** (126 MHz, $CDCl_3$) ∂_C 140.3 (*C*-Ar, visible in HMBC), 128.6 (*C*-Ar), 128.1 (*C*-Ar), 127.2 (*C*-Ar), 92.0 (*C*-β, dd, *J* = 199.6, 17.1 Hz), 88.3 (*C*-γ, dd, *J* = 199.6, 19.4 Hz), 66.2 (*C*-ε, d, *J* = 15.3 Hz), 39.6 (*C*-α, dd, *J* = 24.7, 5.8 Hz), 32.6 (*C*-δ, *C*-ζ, m) **HRMS** (ESI$^+$) *m/z* calc. for $C_{12}H_{18}ONF_2$ $[M+NH_4]^+$ 230.1351, found 230.1356.

7.6 (1*R*, 3*R*, 4*S*, 5*R*)-3,4-difluoro-5-phenylcyclohexan-1-ol 12a

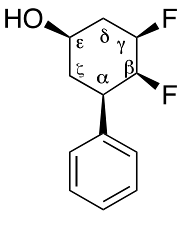

^{1}H NMR (500 MHz, $CDCl_3$) ∂_H 7.36 (2H, m, *H*-Ar), 7.30 (3H, m, *H*-Ar), 4.91 (1H, dd, *J* = 52.6, 8.4 Hz, *H*-β), 4.61 (1H, m, *H*-γ), 3.89 (1H, m, *H*-ε), 2.66 (1H, m, *H*-α), 2.40 (1H, dq, *J* = 10.1, 4.9 Hz, *H*- δ_{eq}), 2.04 (3H, m, *H*-δ_{ax}, *H*-ζ) **^{19}F{^{1}H} NMR** (470 MHz, $CDCl_3$) δ_F -213.6 (*F*, d, *J* = 16.1 Hz), -186.8 (*F*, d, *J* = 16.1 Hz) **^{13}C NMR** (126 MHz, $CDCl_3$) ∂_C 139.8 (*C*-Ar), 128.7 (*C*-Ar), 127.9 (*C*-Ar), 127.3 (*C*-Ar), 90.6 (*C*-β, dd, *J* = 160.8, 16.8 Hz), 89.1 (*C*-γ, dd, *J* = 160.8, 19.2 Hz), 67.1 (*C*-ε, d, *J* = 15.3 Hz), 41.7 (*C*-α, dd, *J* = 25.9, 6.8 Hz), 35.1 (*C*-δ, dd, *J* = 21.6, 2.9 Hz), 34.5 (*C*-ζ, d, *J* = 3.5 Hz) **HRMS** (ESI$^+$) *m/z* calc. for $C_{12}H_{18}ONF_2$ $[M+NH_4]^+$ 230.1351, found 230.1356.

7.7 (1*S*, 3*R*, 4*R*)-4-fluoro-3-phenylcyclohexan-1-ol 13

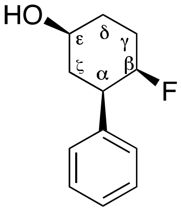

^{1}H NMR (500 MHz, $CDCl_3$) ∂_H 7.33 (5H, m, *H*-Ar), 4.79 (1H, ddd, *J* = 49.0, 3.8, 1.9 Hz, *H*-β), 3.85 (1H, m, *H*-ε), 2.78 (1H, m, *H*-α), 2.24 (1H, m, *H*-γ_{ax}), 2.07 (2H, m, *H*-ζ), 1.94 (1H, m, *H*-δ_{eq}), 1.70 (2H, m, *H*-δ_{ax}, *H*-γ_{eq}) **^{19}F{^{1}H} NMR** (470 MHz, $CDCl_3$) δ_F -197.5 (*F*, s) **^{13}C NMR** (126 MHz, $CDCl_3$) ∂_C 141.5 (*C*-Ar, visible in HMBC), 128.5 (*C*-Ar), 128.0 (*C*-Ar), 126.8 (*C*-Ar), 90.4 (*C*-β, d, *J* = 175.5 Hz), 70.1 (*C*-ε, s), 45.9 (*C*-α, d, *J* = 19.9 Hz), 35.5 (*C*-ζ, d, *J* = 2.9 Hz), 29.7 (*C*-γ, d, *J* = 21.4 Hz), 19.1 (*C*-δ, s) **HRMS** (ESI^+) *m/z* calc. for $C_{12}H_{19}ONF$ $[M+NH_4]^+$ 212.1445, found 212.1445.

7.8 4-((1*R*, 2*S*, 3*R*, 6*S*)-2,3,6-trifluoro-1-hydroxycyclohexyl)benzoic acid 15

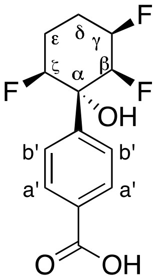

^{1}H NMR (500 MHz, MeOD) ∂_H 8.06 (2H, m, *H*-Ar-*a'*), 7.85 (2H, d, *J* = 8.5 Hz, *H*-Ar-*b'*), 5.11 (2H, m, *H*-γ, *H*-ζ), 4.79 (1H, d, *J* = 46.9 Hz, *H*-β), 2.21 (3H, m, *H*-ε, *H*-δ_{eq}), 1.91 (1H, m, δ_{ax}) **^{19}F{^{1}H} NMR** (470 MHz, MeOD) δ_F -209.9 (*F*, dd, *J* = 23.0, 12.8 Hz), -192.9 (*F*, d, *J* = 12.8 Hz), -191.2 (*F*, d, *J* = 23.0 Hz) **^{13}C NMR** (126 MHz, MeOD) ∂_C 169.4 (*C*OOH, visible in HMBC), 149.1 (*C*-Ar, visible in HMBC), 132.0 (*C*-Ar, visible in HMBC), 129.0 (*C*-Ar), 126.6 (*C*-Ar), 91.2 (*C*-ζ, dd, *J*= 203.6, 15.5 Hz), 90.8 (*C*-β, d, *J*= 178.3 Hz), 88.5 (*C*-γ, dd, *J*= 198.2, 19.9 Hz), 73.0 (*C*-α, visible in HMBC), 24.5 (*C*-ε, dd, *J*= 34.4, 12.6 Hz), 19.7 (*C*-δ, dd, *J*= 24.1, 4.1 Hz) **HRMS** (ESI^-) *m/z* calc. for $C_{13}H_{12}O_3F_3$ [M]-H 273.0739, found 273.0743.

4-((1*s*, 2*R*, 3*S*, 5*R*, 6*S*)-2,3,5,6-tetrafluoro-1-hydroxycyclohexyl)benzoic acid 14

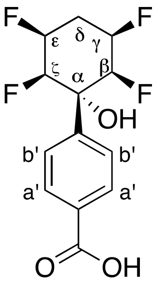

^{19}F NMR (470 MHz, MeOD) δ_F -210.0 (2F, m), -200.0 (2F, dd, *J* = 44.9 Hz) **HRMS** (ESI^-) *m/z* calc. for $C_{13}H_{11}O_3F_4$ [M]-H 291.0644, found 291.0651.

8. NMR spectra of the fluorometabolites

8.1 2,3,5,6-Tetrafluoro-1-phenylcyclohexan-1-ol (8)

^{1}H NMR (500 MHz, $CDCl_3$)

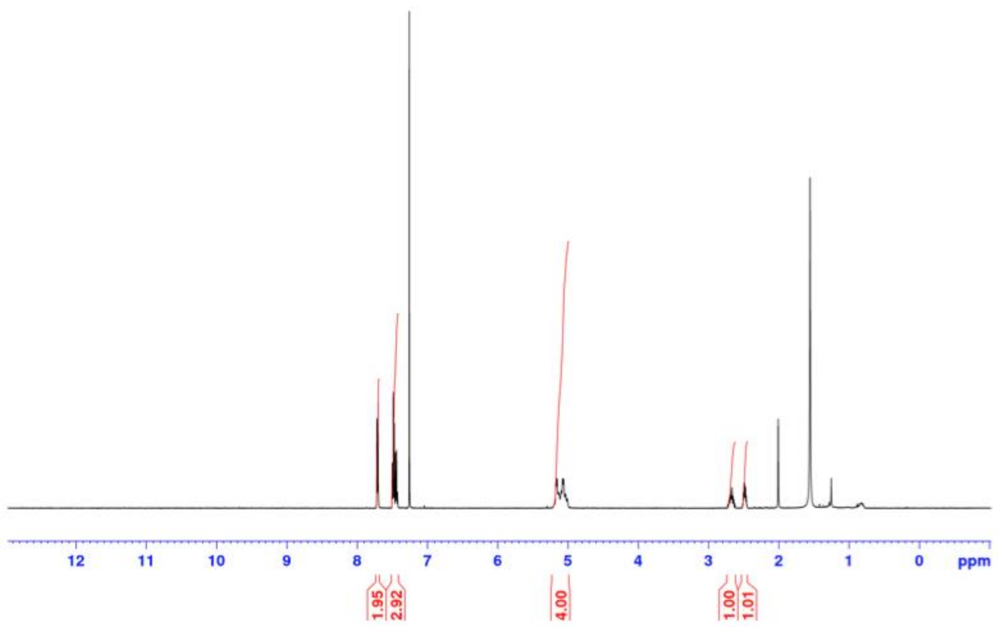

^{19}F{^{1}H} NMR (470 MHz, $CDCl_3$)

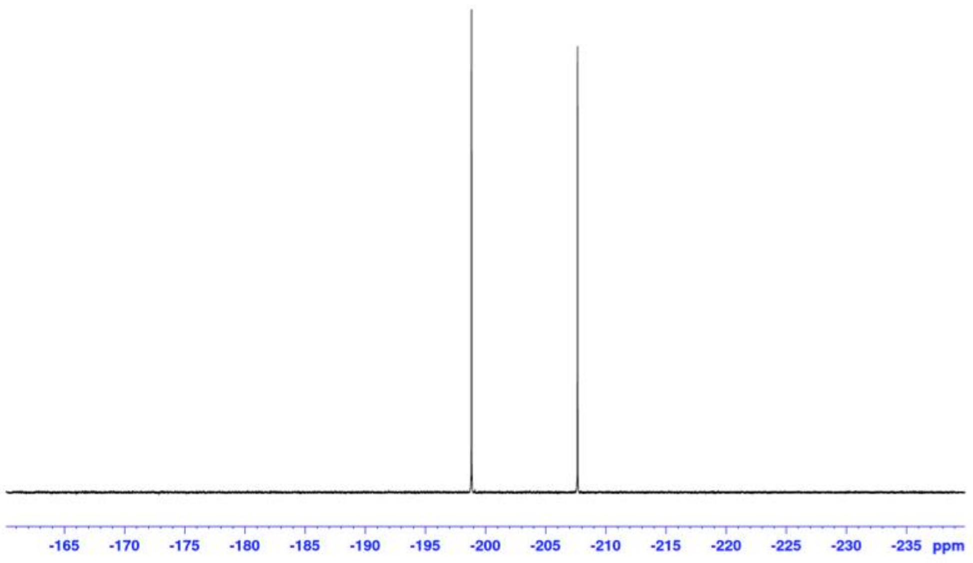

^{13}**C NMR** (126 MHz, $CDCl_3$)

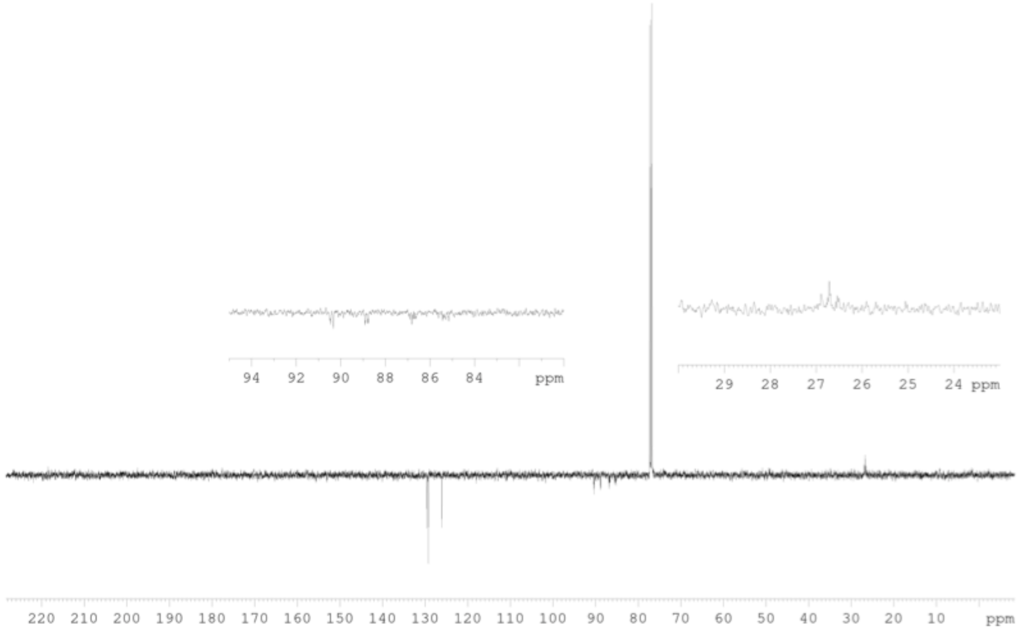

COSY

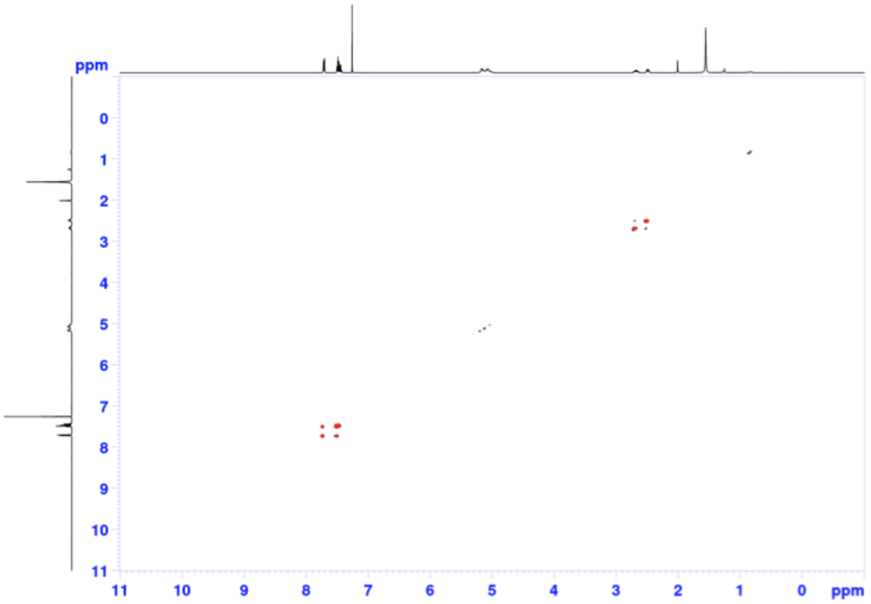

HSQC

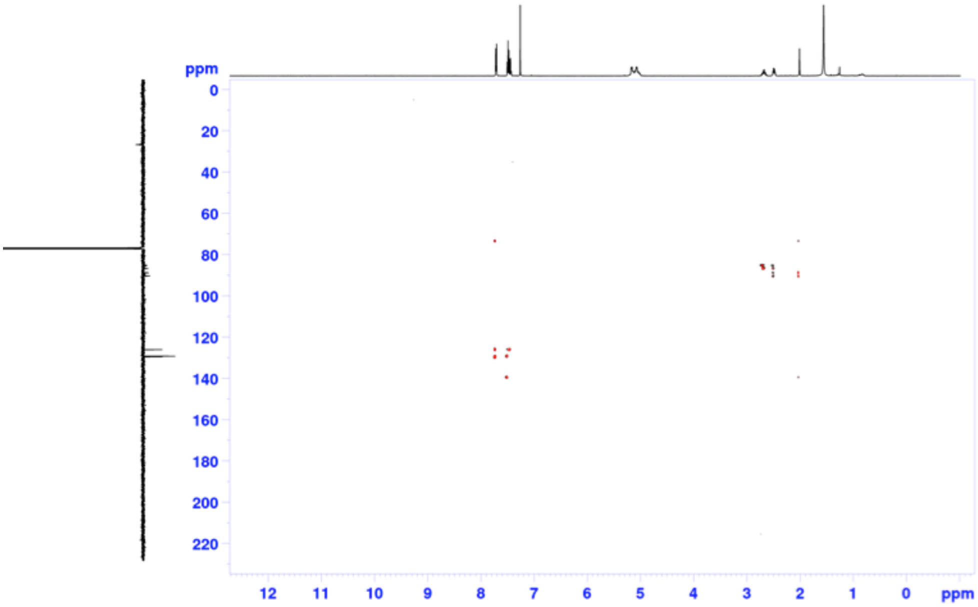
ppm
0
20
40
60
80
100
120
140
160
180
200
220
12
11
10
9
8
7
6
5
4
3
2
1
0
ppm

HMBC

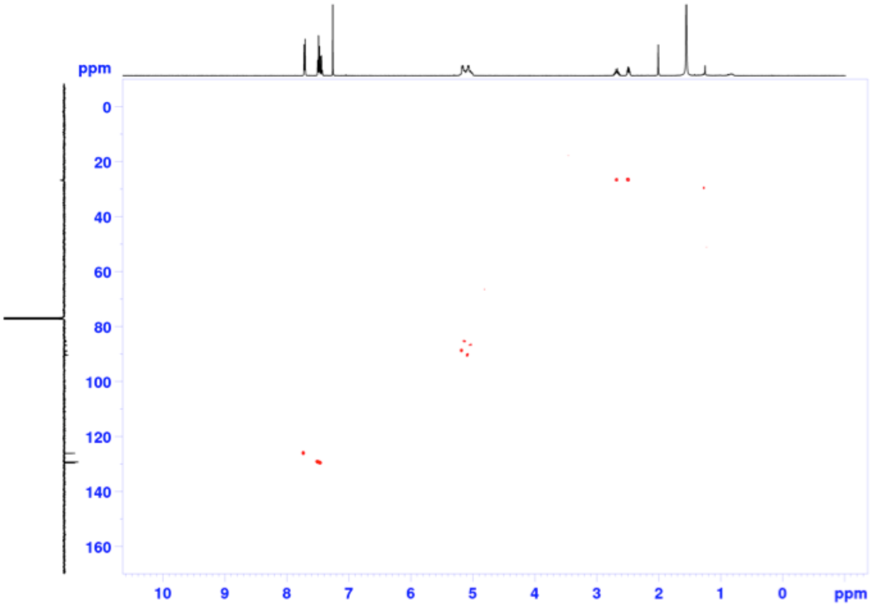
ppm
0
20
40
60
80
100
120
140
160
10
9
8
7
6
5
4
3
2
1
0
ppm

8.2 2,3,6-Trifluoro-1-phenylcyclohexan-1-ol (9)

^{1}H NMR (500 MHz, $CDCl_3$)

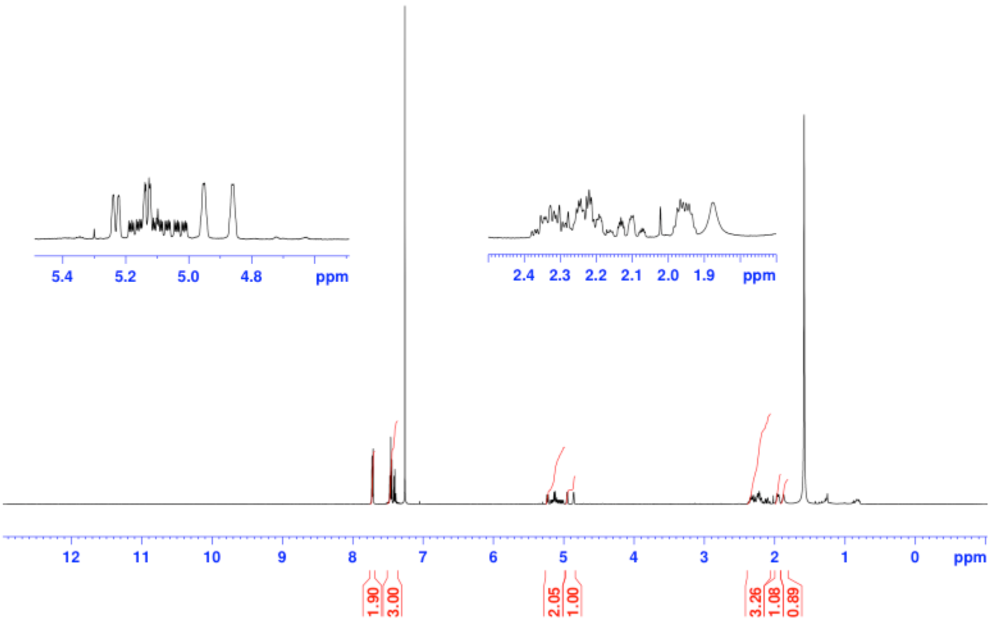

^{19}F{^{1}H} NMR (470 MHz, $CDCl_3$)

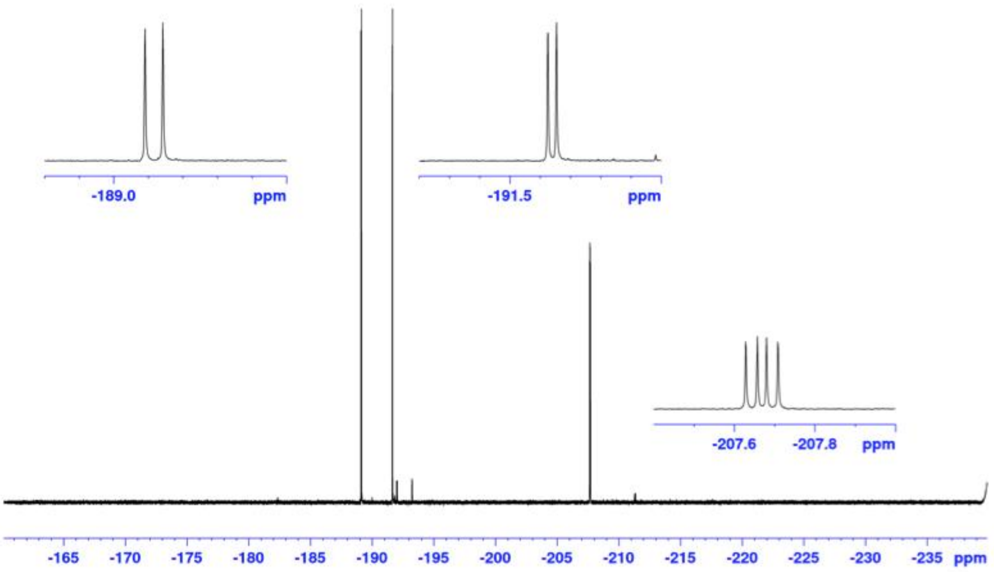

^{13}C NMR (126 MHz, $CDCl_3$)

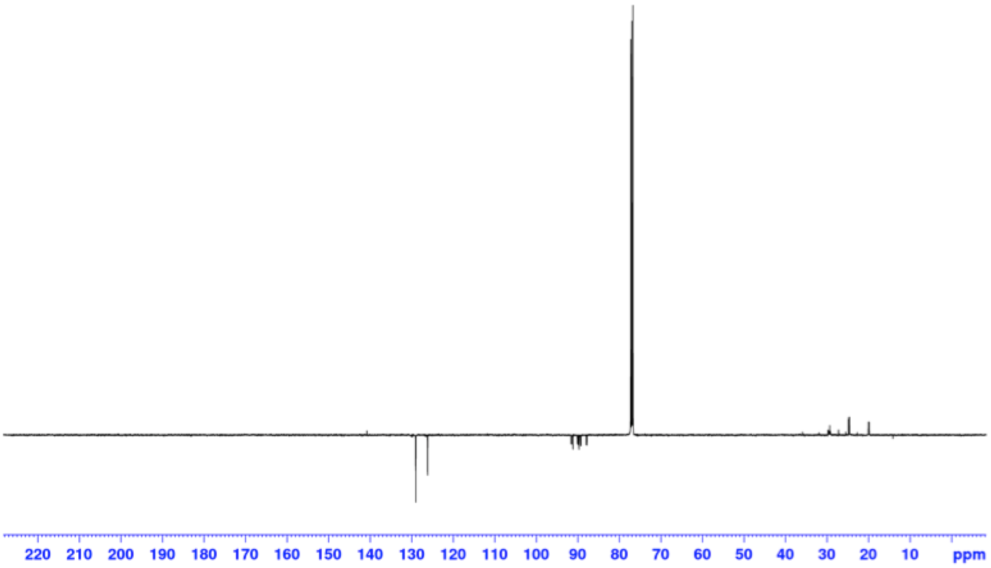

COSY

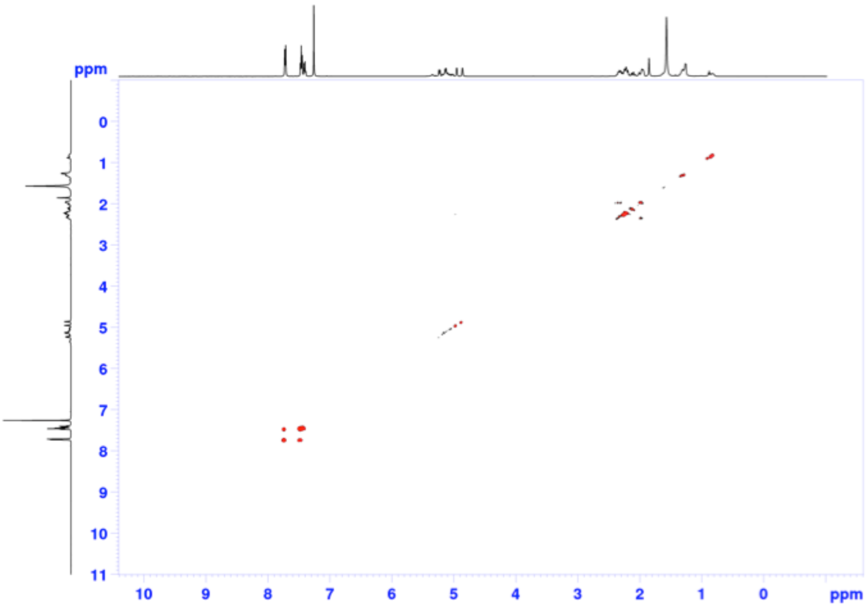

HSQC

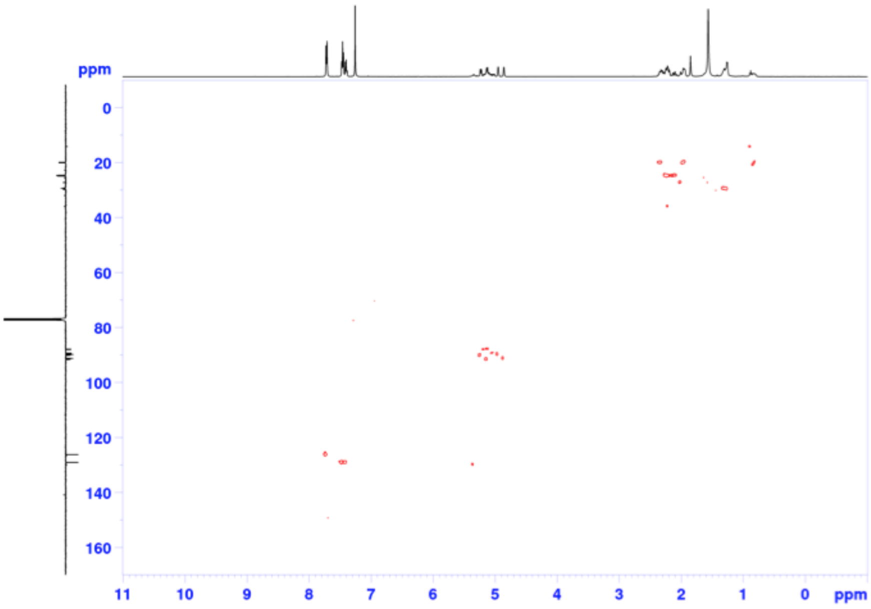
ppm
0
20
40
60
80
100
120
140
160
11 10 9 8 7 6 5 4 3 2 1 0 ppm

HMBC

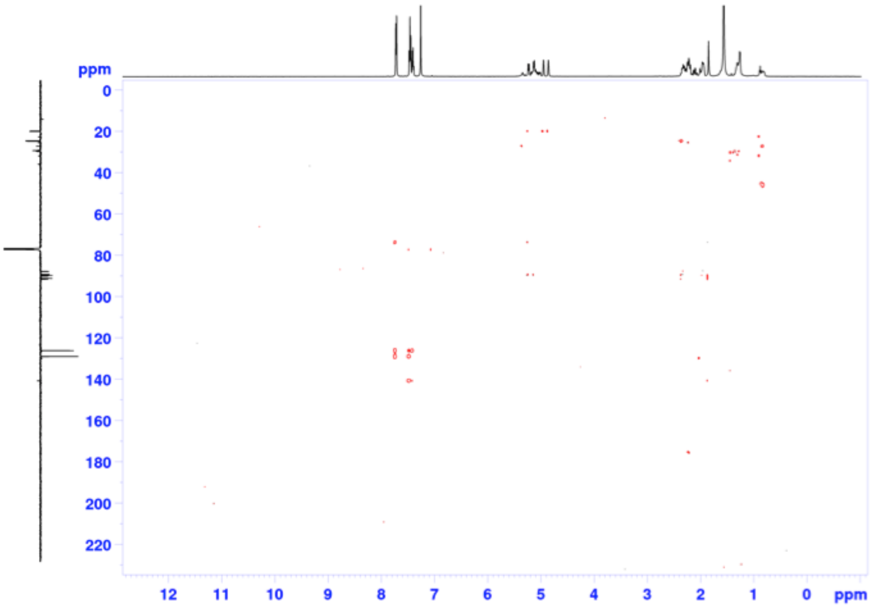
ppm
0
20
40
60
80
100
120
140
160
180
200
220
12 11 10 9 8 7 6 5 4 3 2 1 0 ppm

8.3 2,3-Difluoro-1-phenylcyclohexan-1-ol (10)

^{1}H NMR (500 MHz, $CDCl_3$)

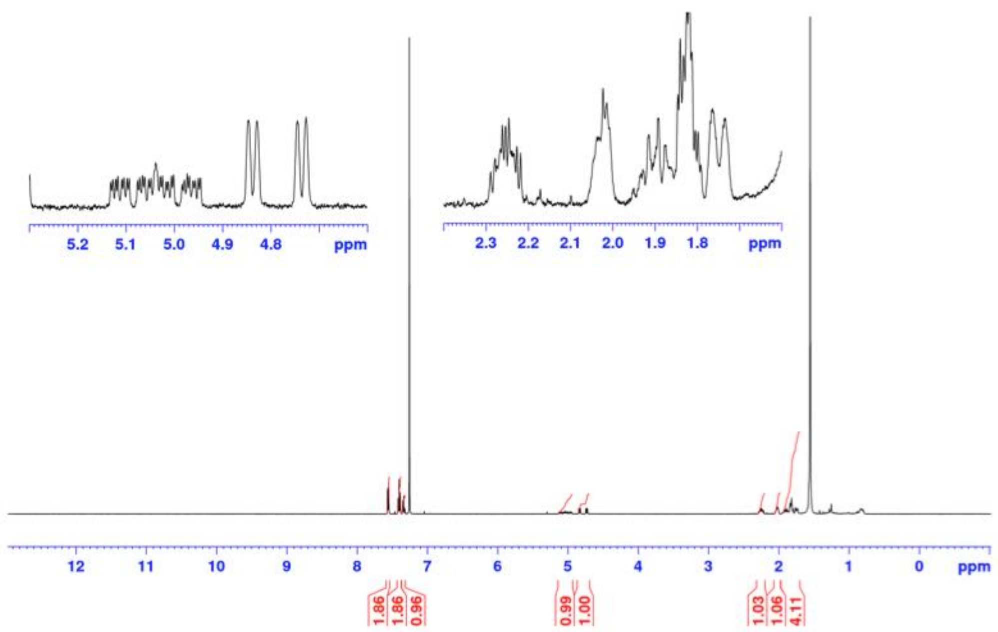

^{19}F{^{1}H} NMR (470 MHz, $CDCl_3$)

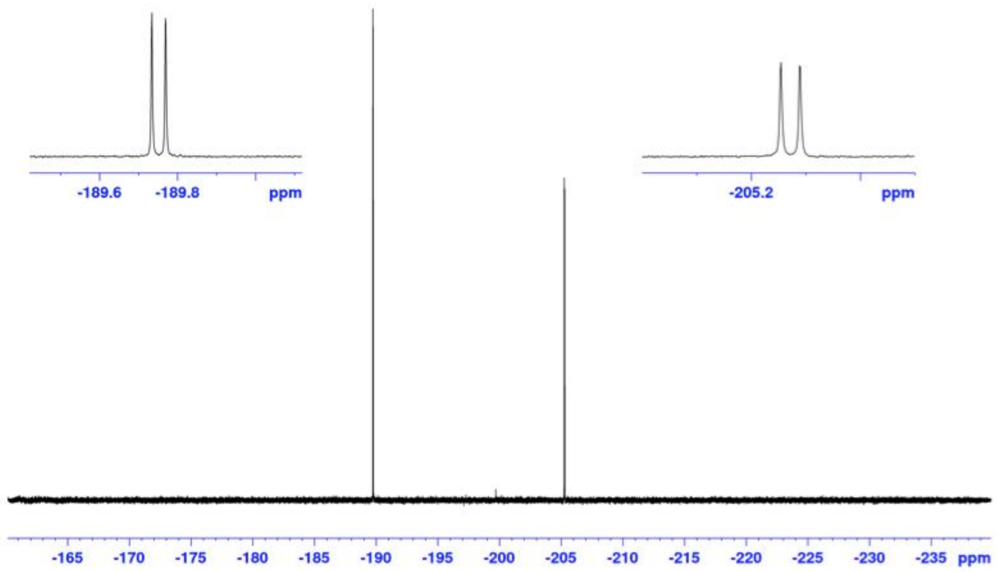

^{13}C NMR (126 MHz, $CDCl_3$)

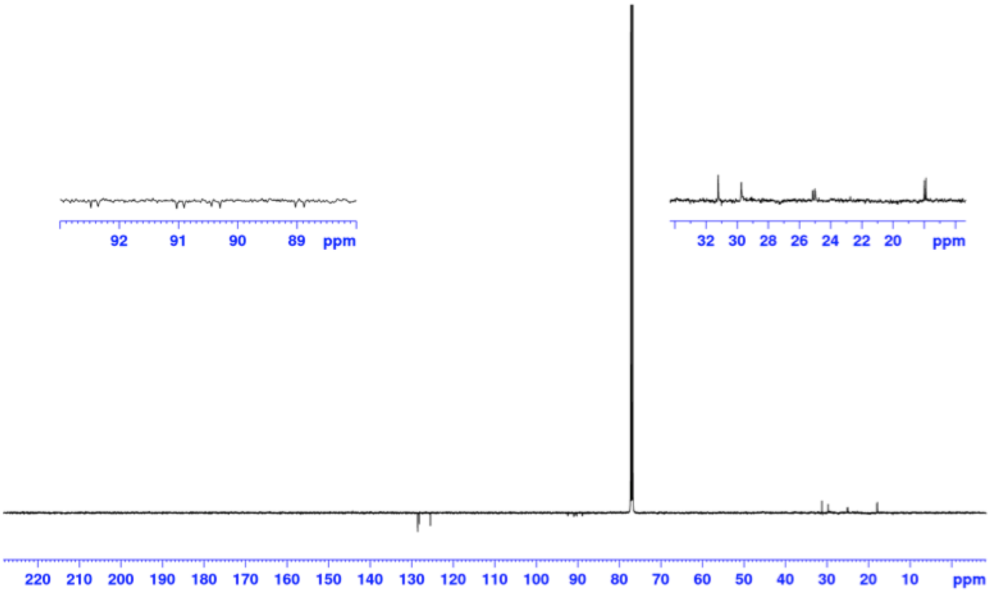

COSY

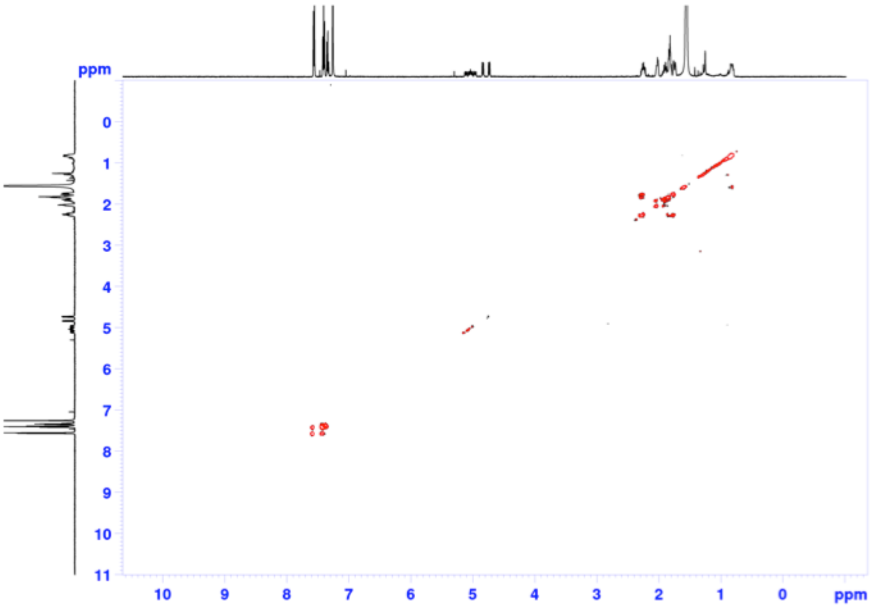

HSQC

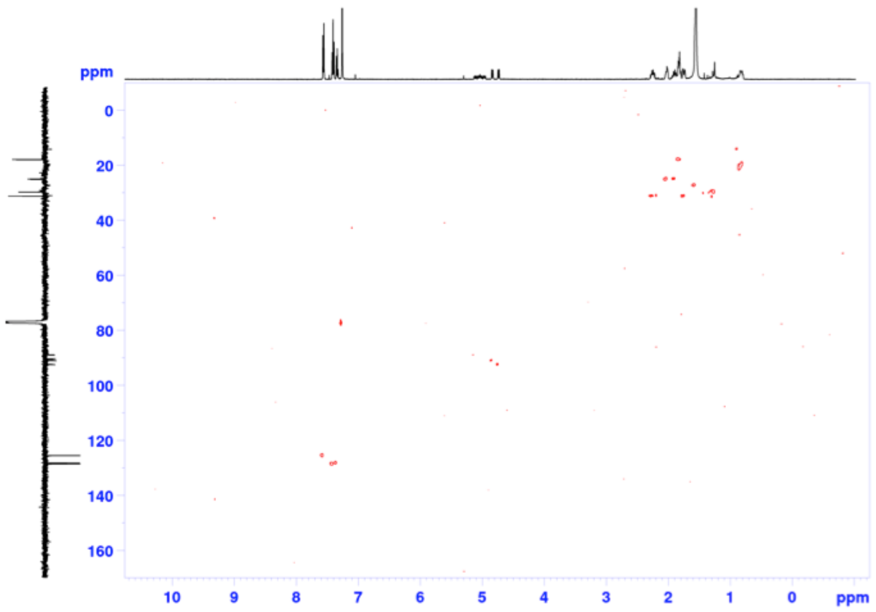
ppm
0
20
40
60
80
100
120
140
160
10
9
8
7
6
5
4
3
2
1
0
ppm

HMBC

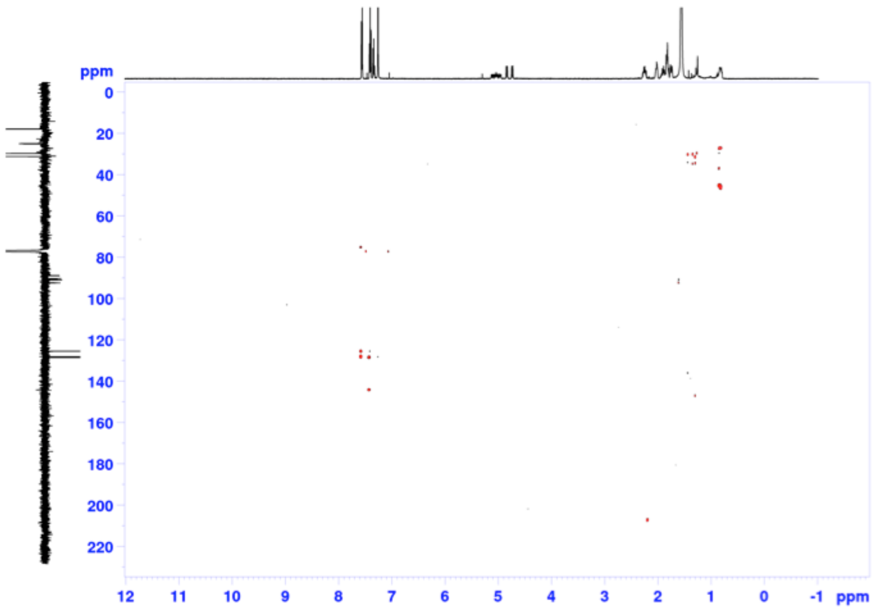
ppm
0
20
40
60
80
100
120
140
160
180
200
220
12
11
10
9
8
7
6
5
4
3
2
1
0
-1
ppm

8.4 2,3-Difluoro-4-phenylcyclohexan-1-ol (11)

^{1}H NMR (500 MHz, $CDCl_3$)

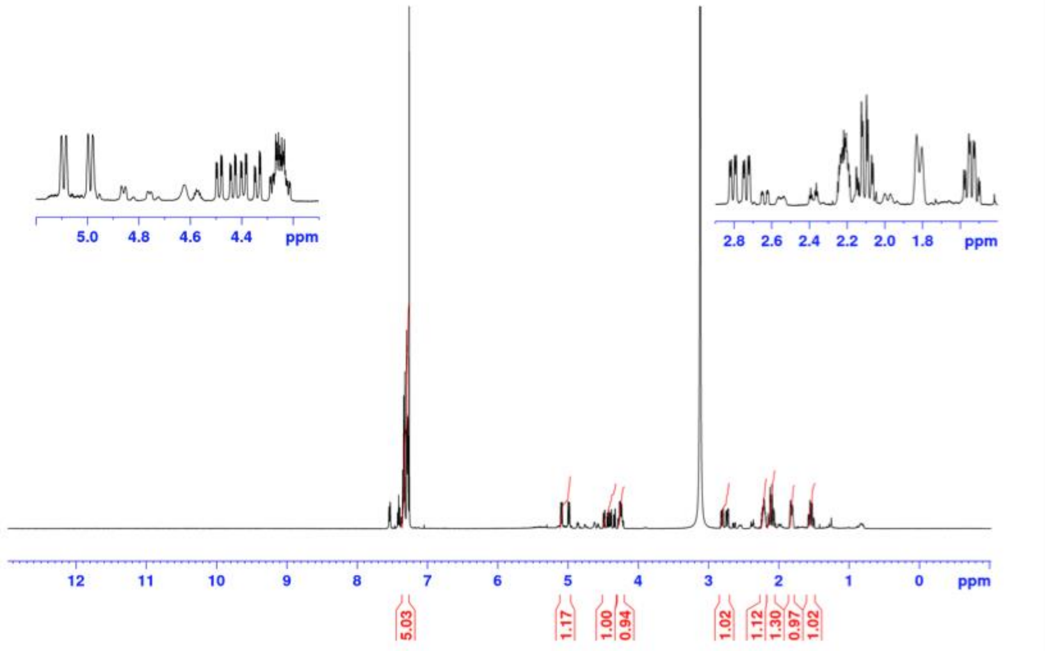

^{19}F{^{1}H} NMR (470 MHz, $CDCl_3$)

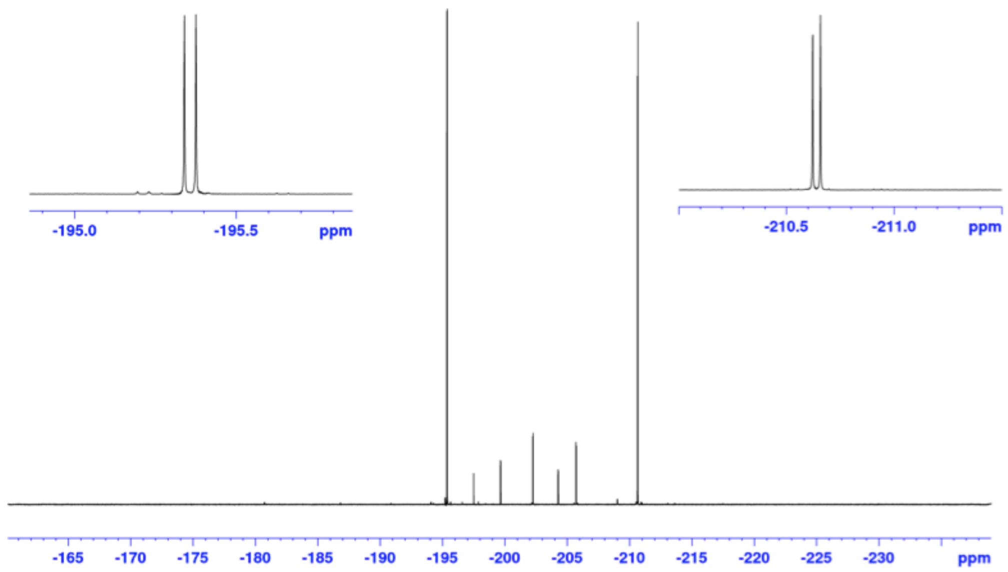

^{13}C NMR (126 MHz, $CDCl_3$)

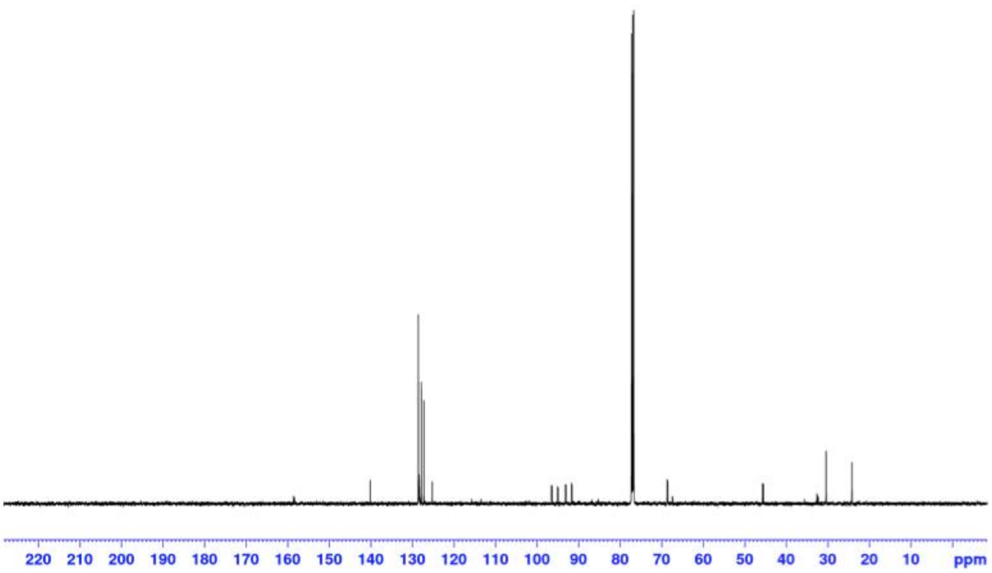

COSY

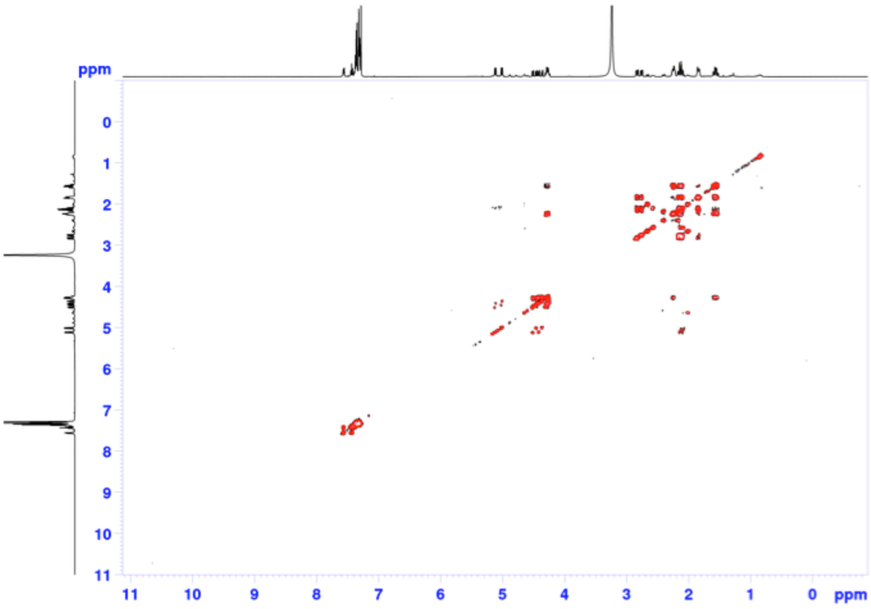

HSQC

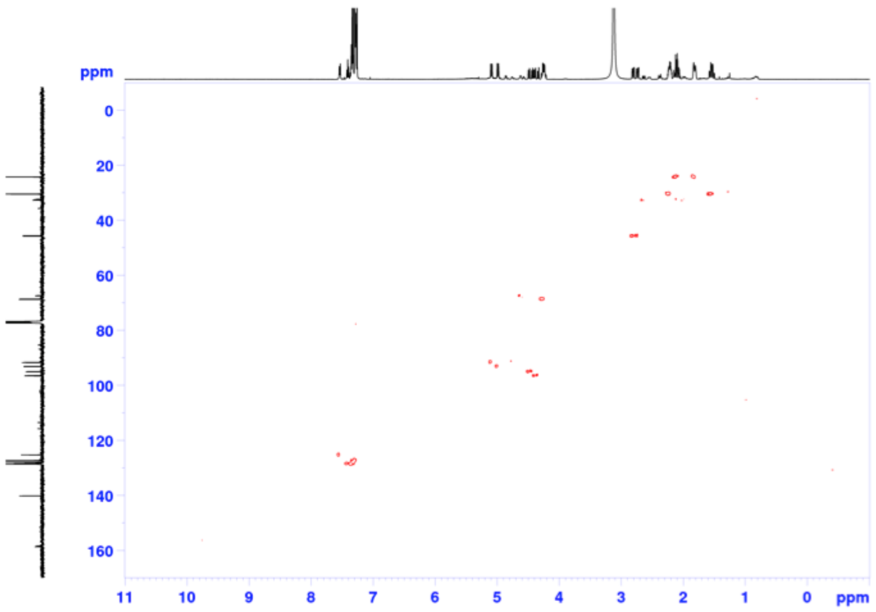
ppm
0
20
40
60
80
100
120
140
160
11
10
9
8
7
6
5
4
3
2
1
0
ppm

HMBC

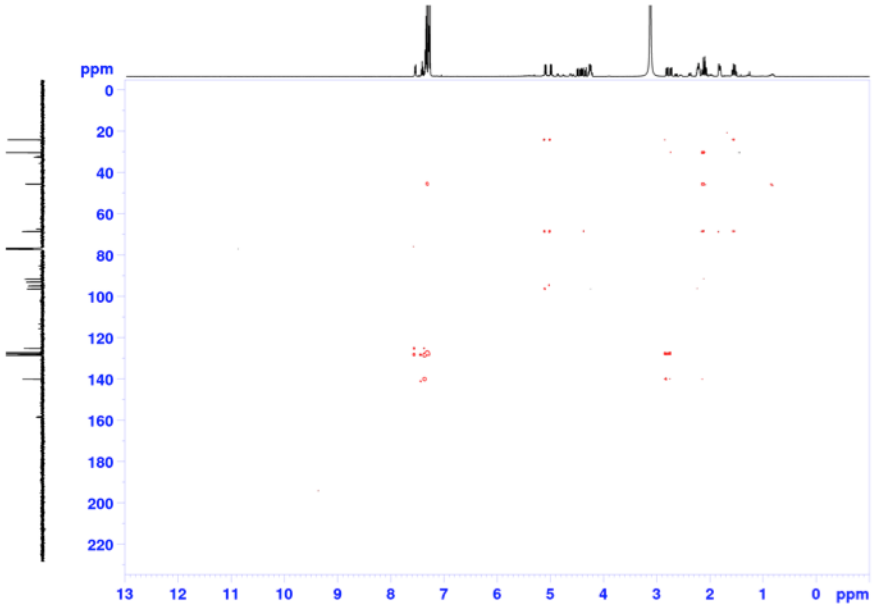
ppm
0
20
40
60
80
100
120
140
160
180
200
220
13
12
11
10
9
8
7
6
5
4
3
2
1
0
ppm

8.5 3,4-Difluoro-5-phenylcyclohexan-1-ol (12b)

^{1}H NMR (500 MHz, $CDCl_3$)

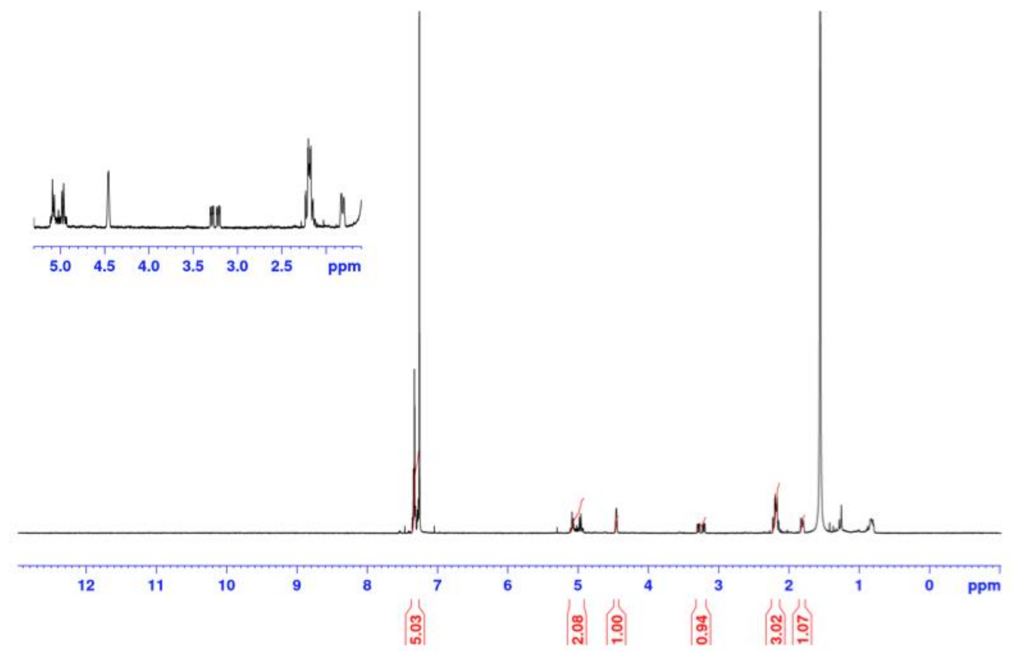

$^{19}F\{^{1}H\}$ NMR (470 MHz, $CDCl_3$)

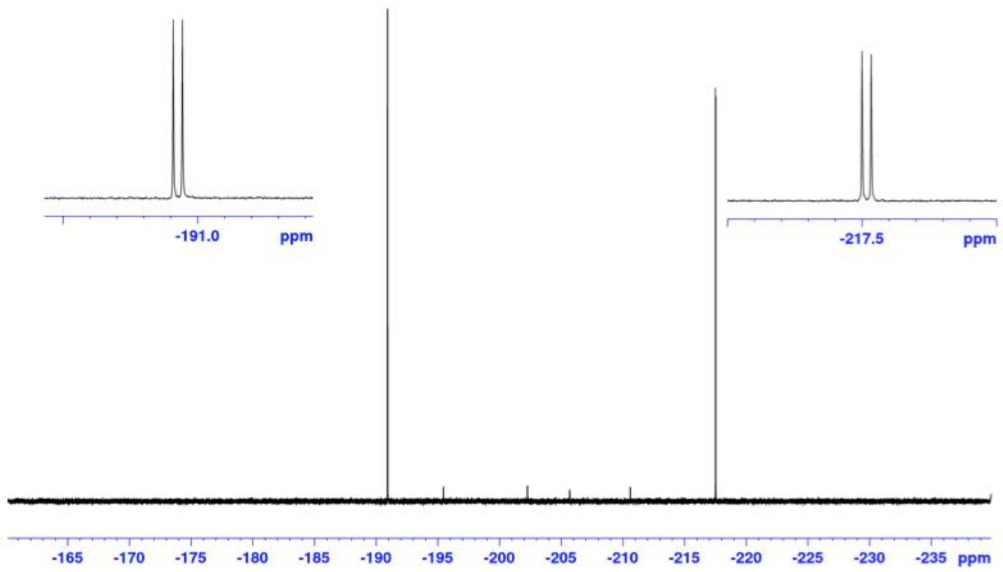

^{13}C NMR (126 MHz, $CDCl_3$)

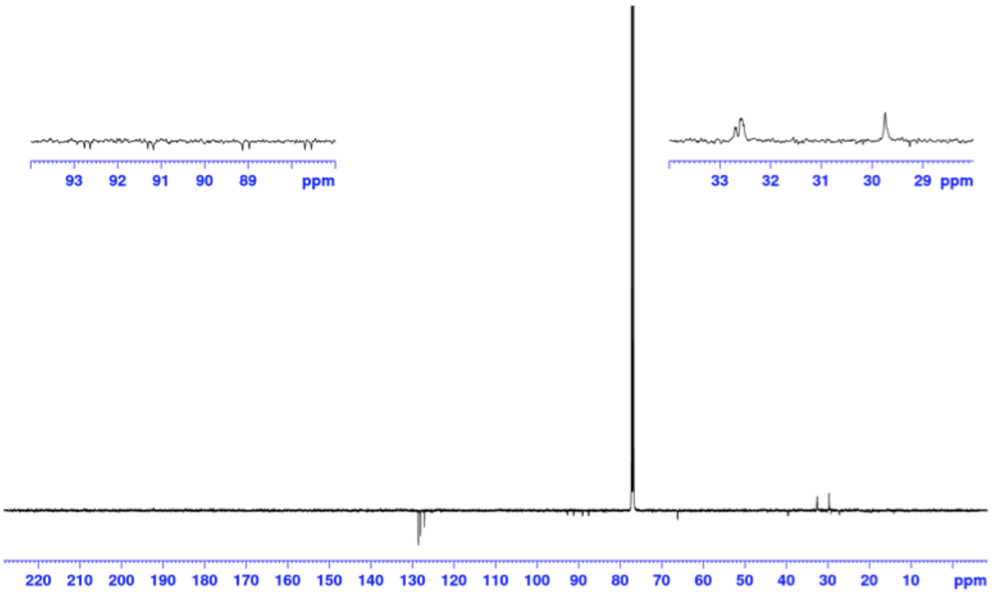

COSY

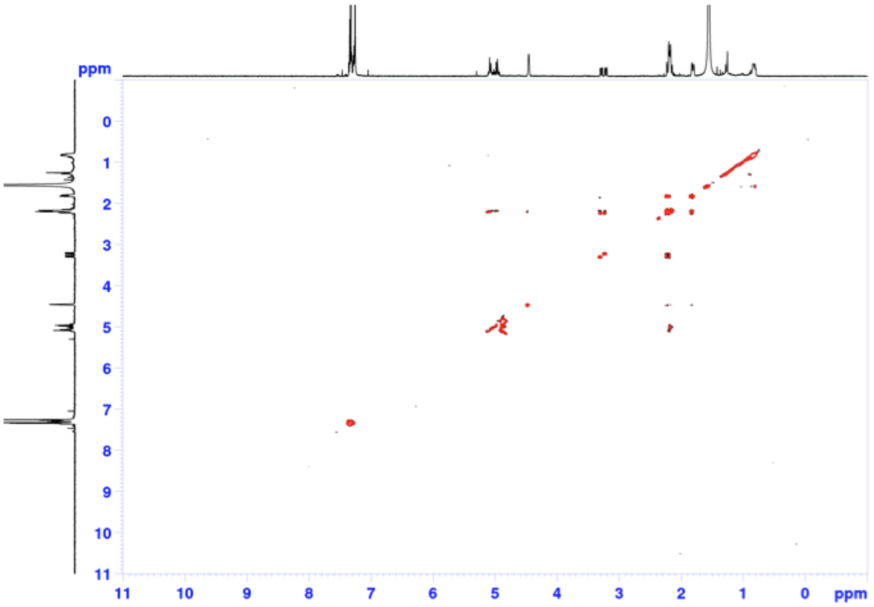

HSQC

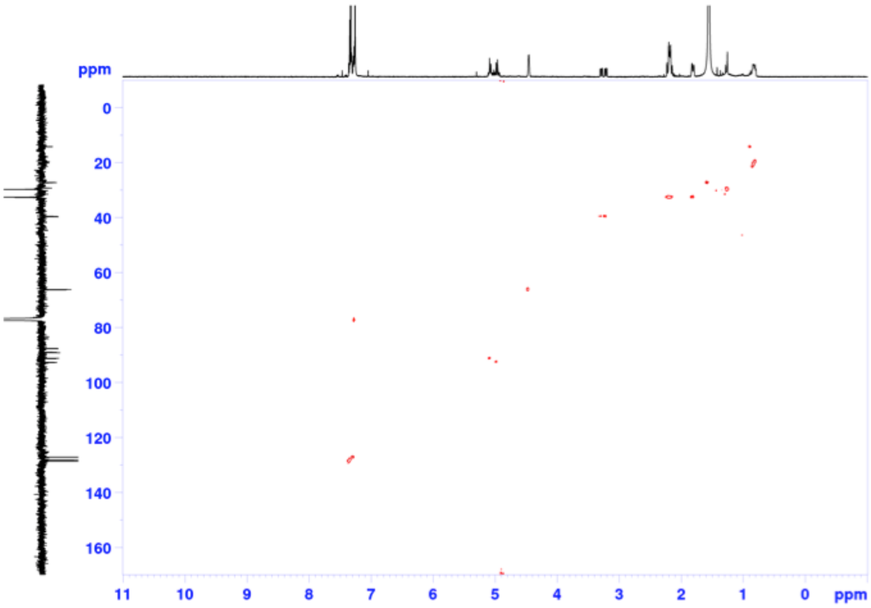
ppm
0
20
40
60
80
100
120
140
160
11
10
9
8
7
6
5
4
3
2
1
0
ppm

HMBC

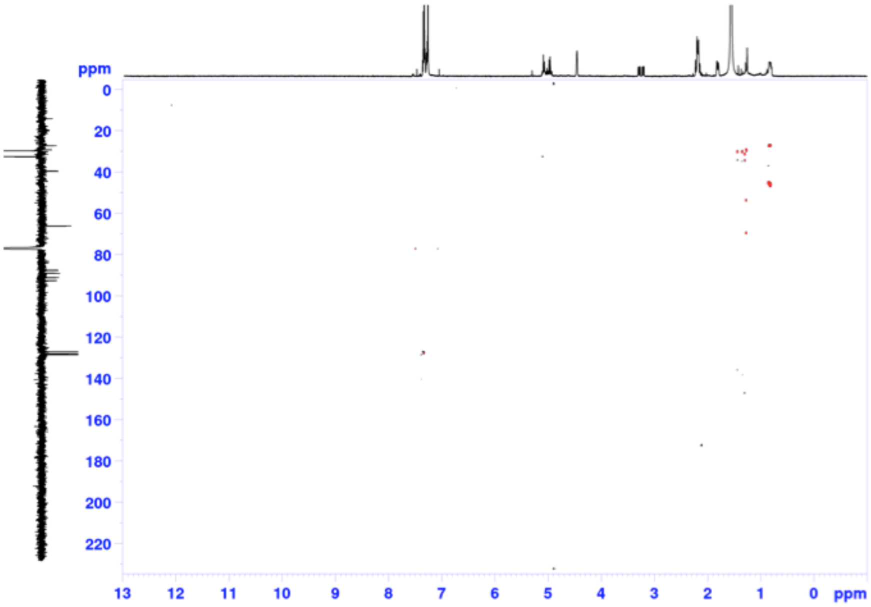
ppm
0
20
40
60
80
100
120
140
160
180
200
220
13
12
11
10
9
8
7
6
5
4
3
2
1
0
ppm

8.6 3,4-Difluoro-5-phenylcyclohexan-1-ol (12a)

^{1}H NMR (500 MHz, $CDCl_3$)

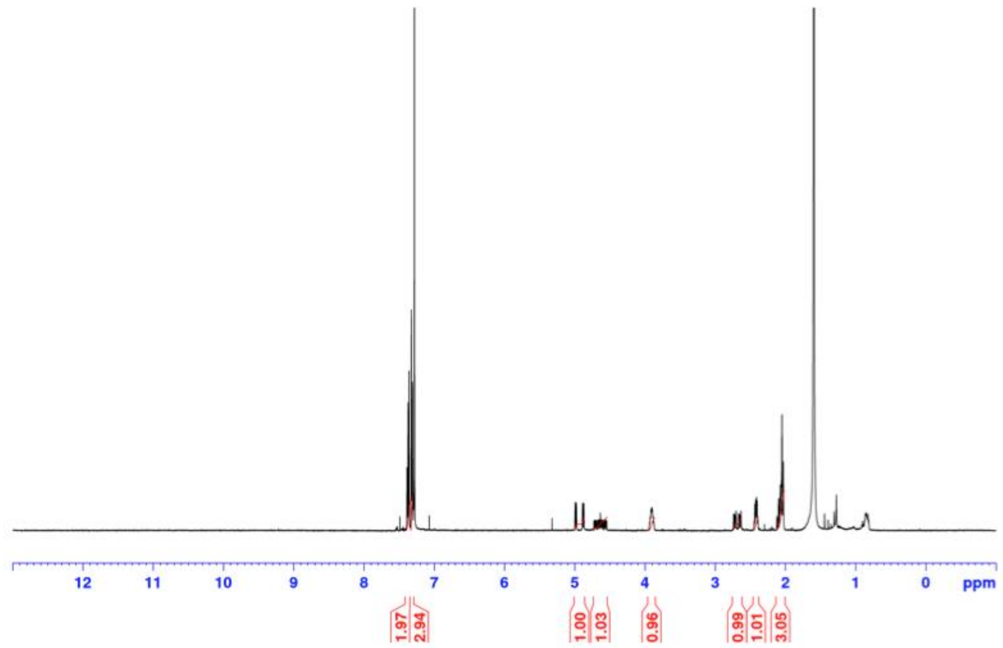

^{19}F{^{1}H} NMR (470 MHz, $CDCl_3$)

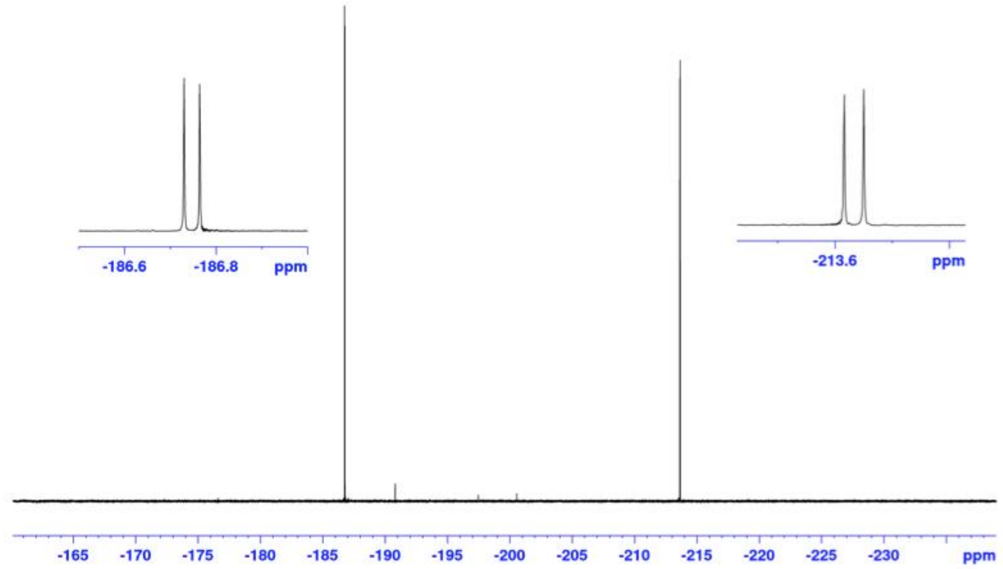

^{13}C NMR (126 MHz, $CDCl_3$)

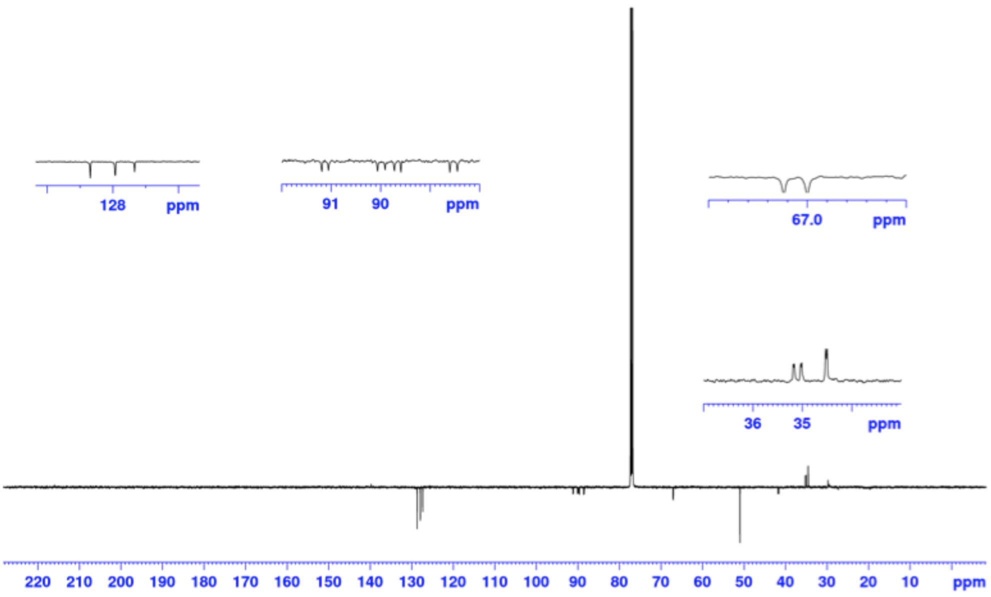

COSY

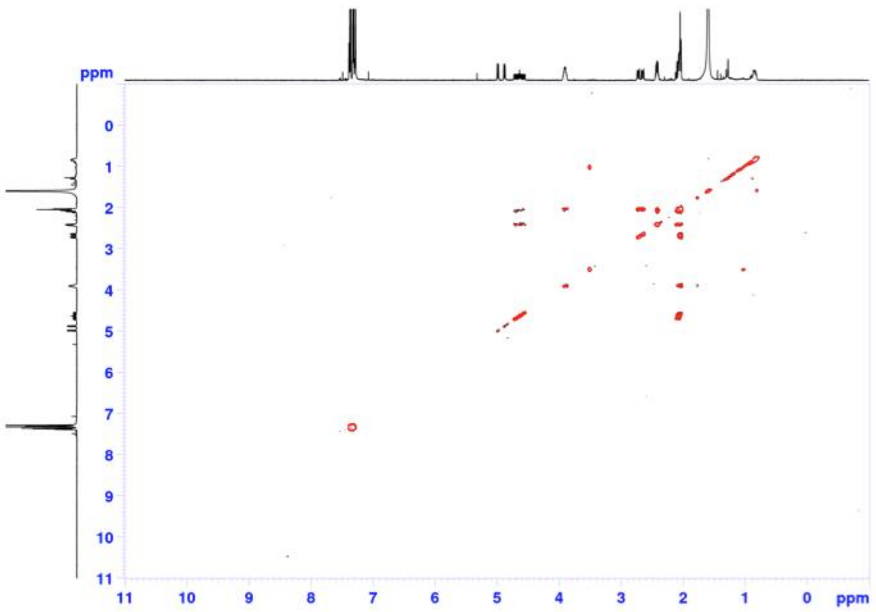

HSQC

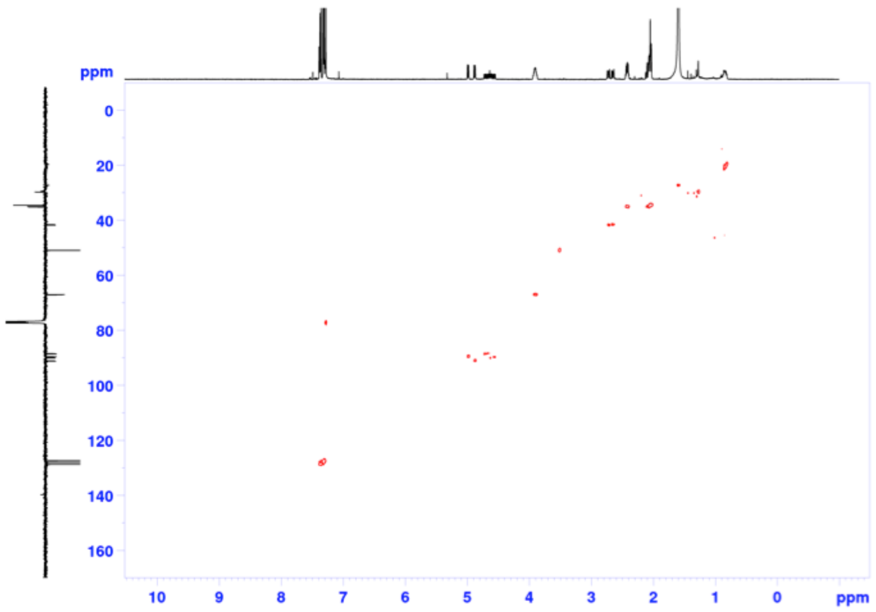

ppm
0
20
40
60
80
100
120
140
160
10
9
8
7
6
5
4
3
2
1
0
ppm

HMBC

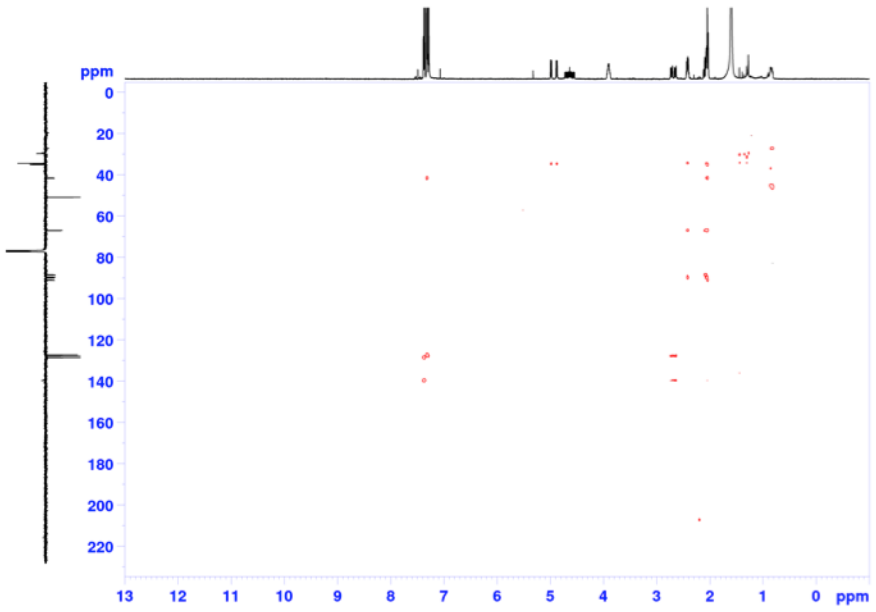

ppm
0
20
40
60
80
100
120
140
160
180
200
220
13
12
11
10
9
8
7
6
5
4
3
2
1
0
ppm

8.7 4-fluoro-3-phenylcyclohexan-1-ol (13)

^{1}H NMR (500 MHz, $CDCl_3$)

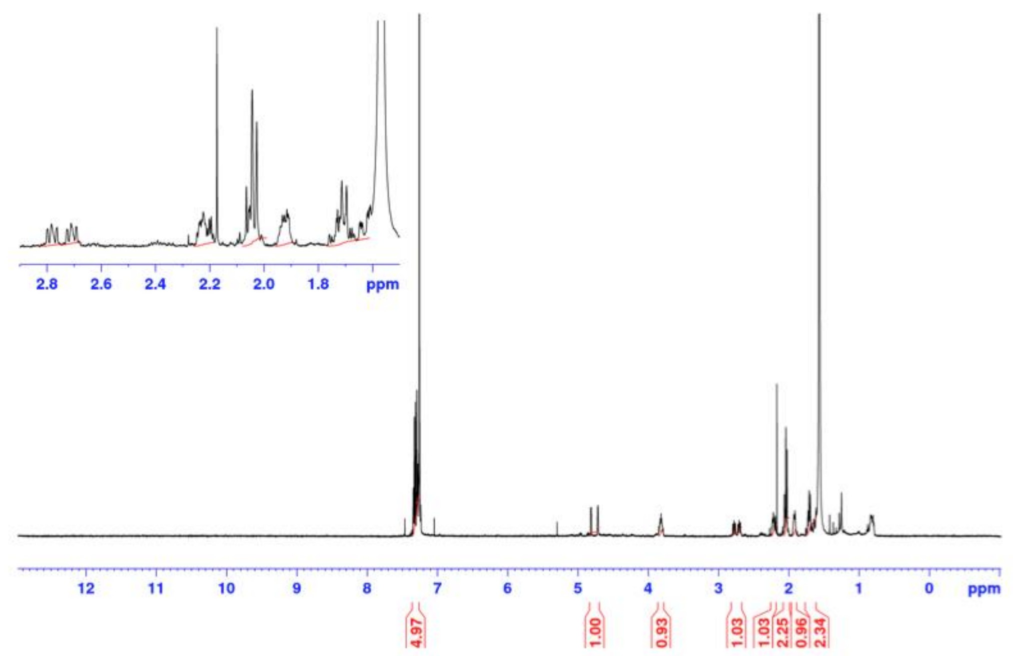

$^{19}F\{^1H\}$ NMR (470 MHz, $CDCl_3$)

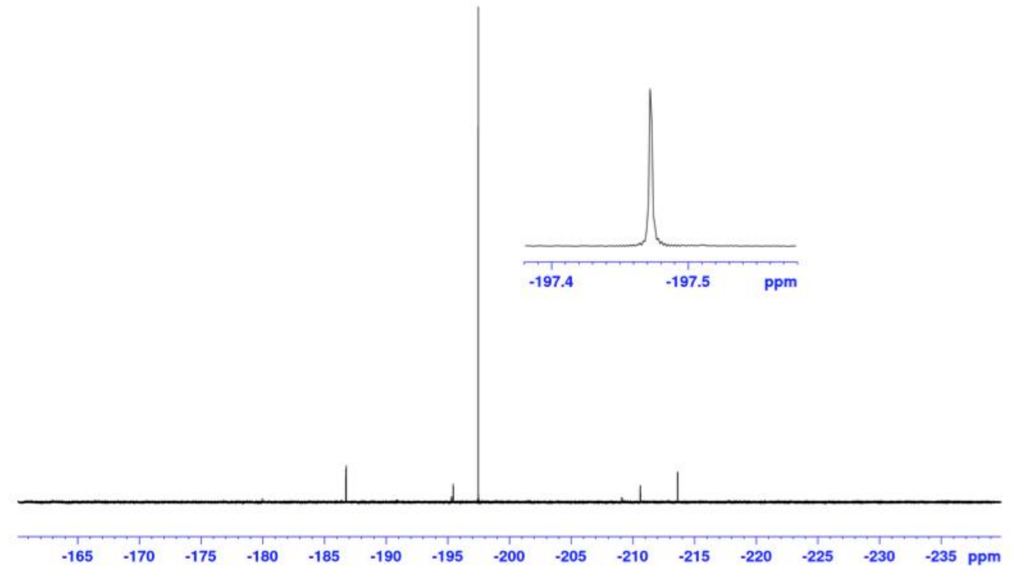

^{13}C NMR (126 MHz, $CDCl_3$)

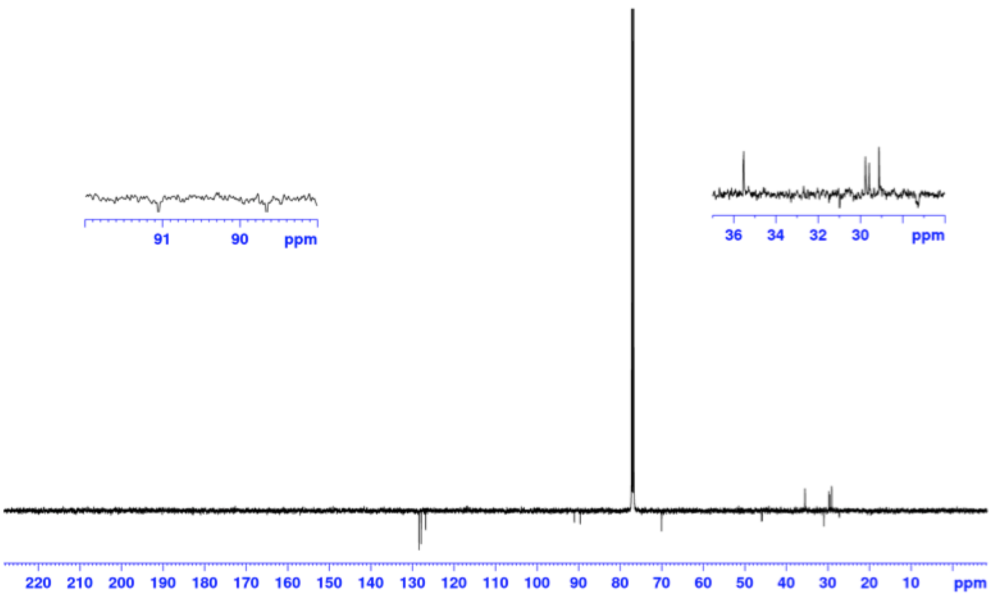

COSY

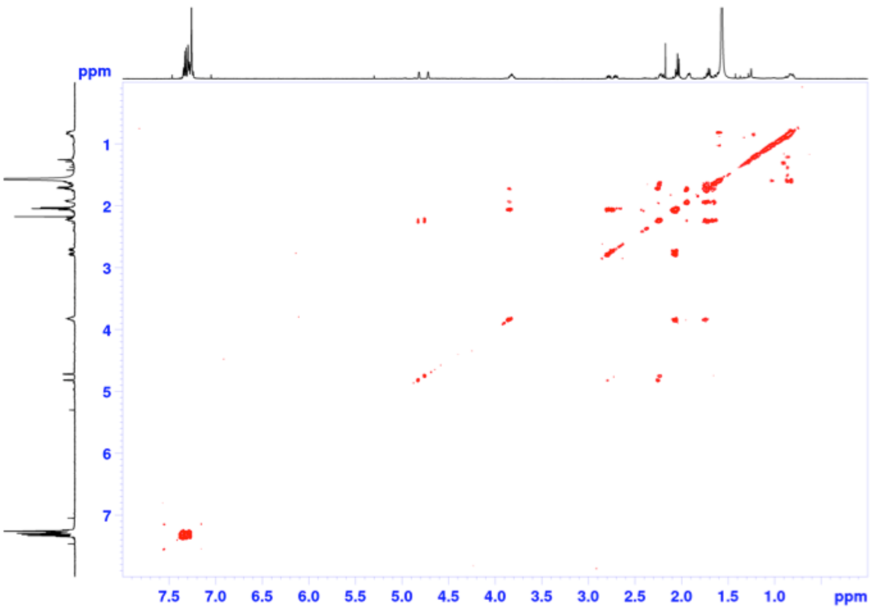

HSQC

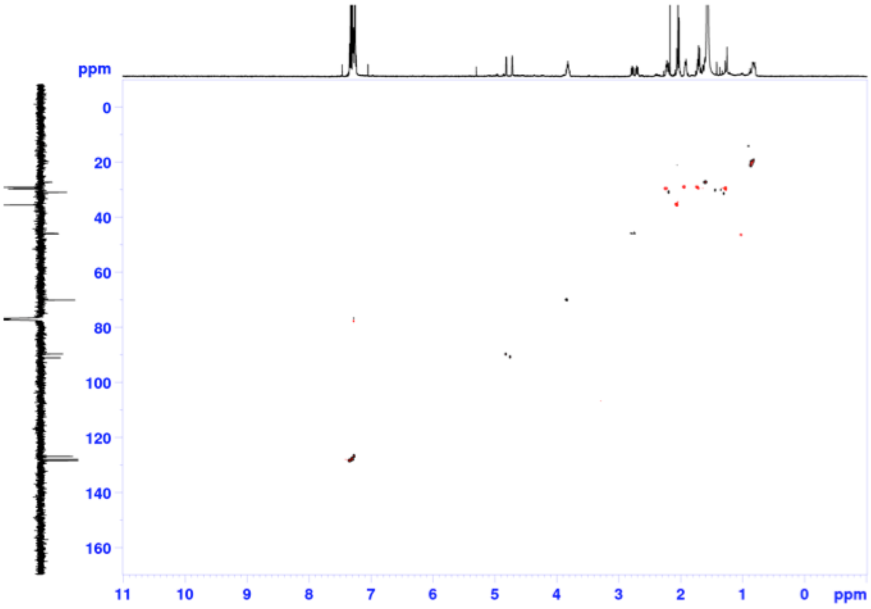
ppm
0
20
40
60
80
100
120
140
160
11
10
9
8
7
6
5
4
3
2
1
0
ppm

HMBC

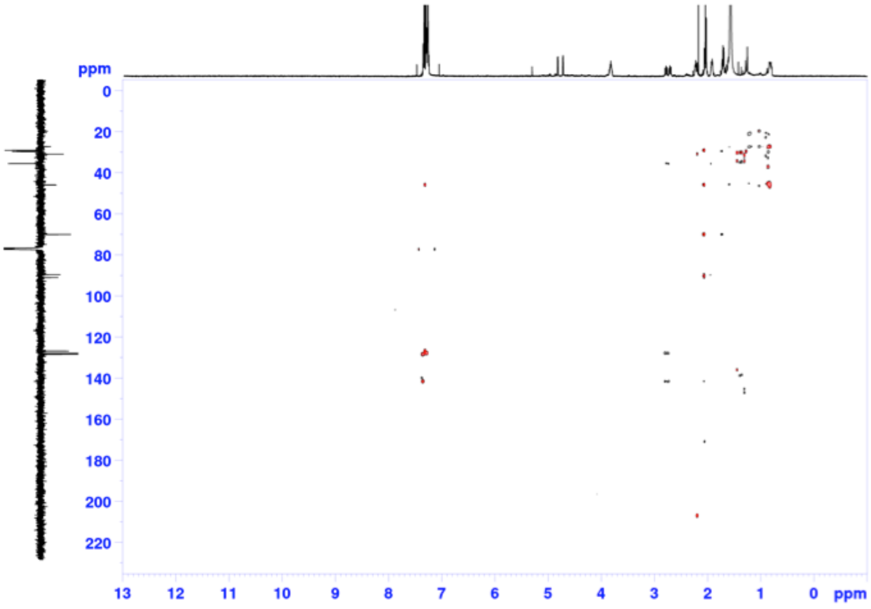
ppm
0
20
40
60
80
100
120
140
160
180
200
220
13
12
11
10
9
8
7
6
5
4
3
2
1
0
ppm

8.8 4-(2,3,6-Trifluoro-1-hydroxycyclohexyl)benzoic acid (15)

^{1}H NMR (500 MHz, MeOD)

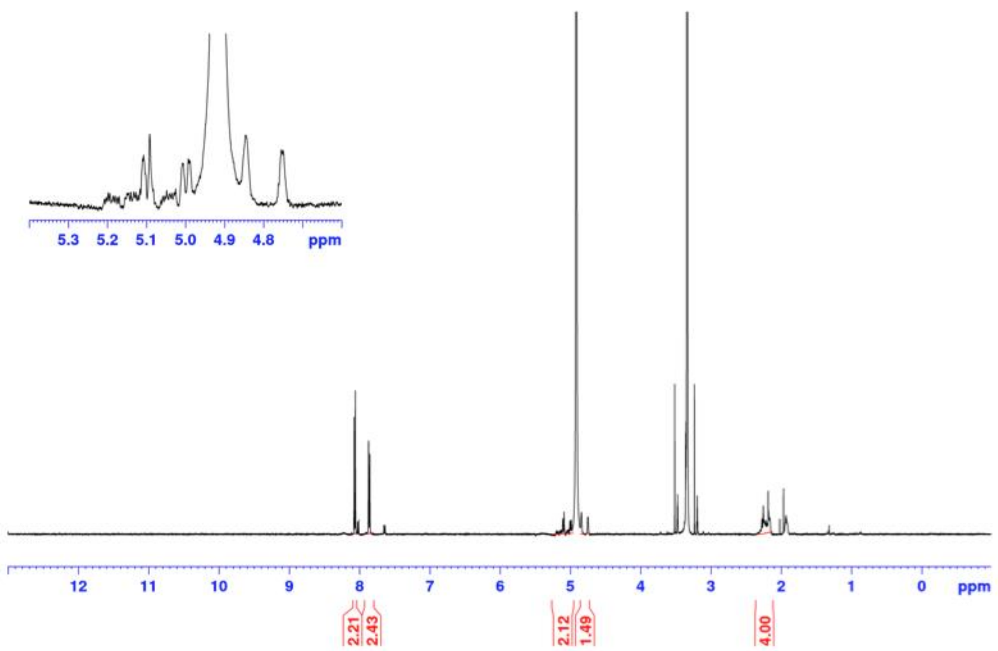

^{19}F{^{1}H} NMR (470 MHz, MeOD)

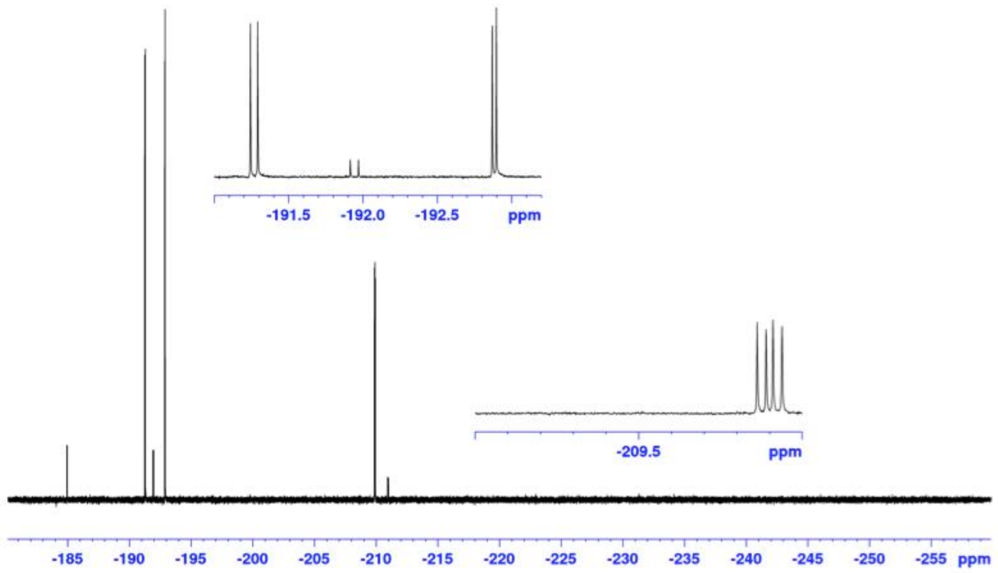

^{13}C NMR (126 MHz, MeOD)

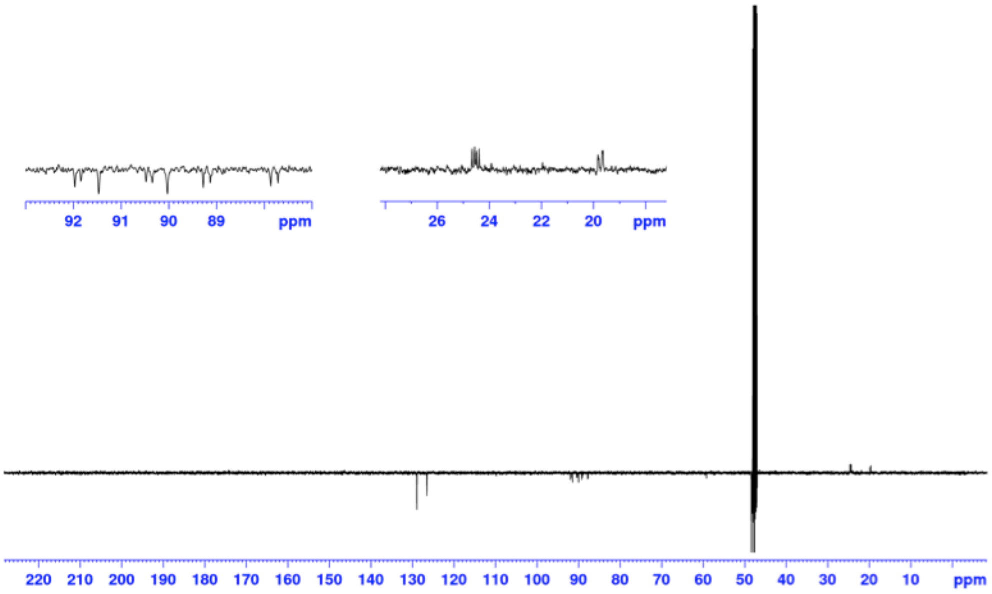

COSY

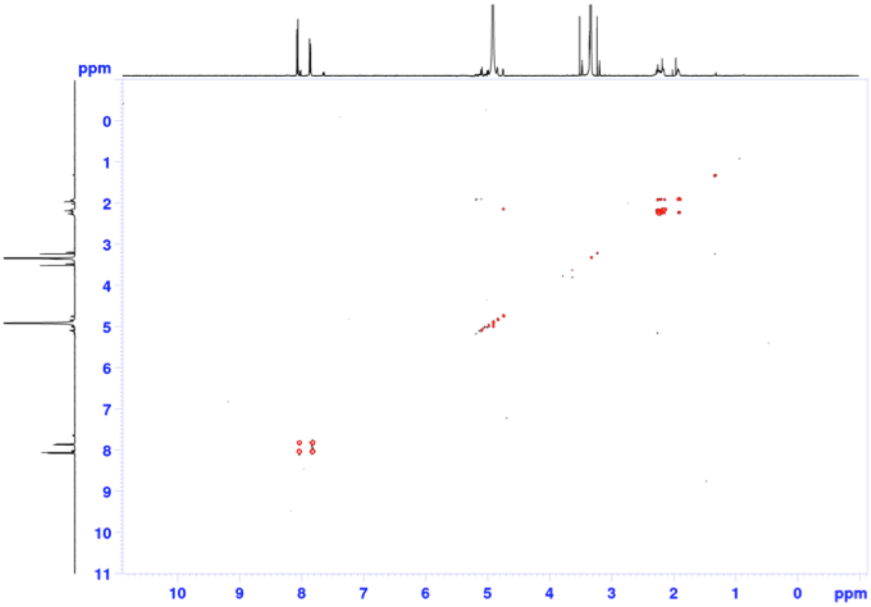

HSQC

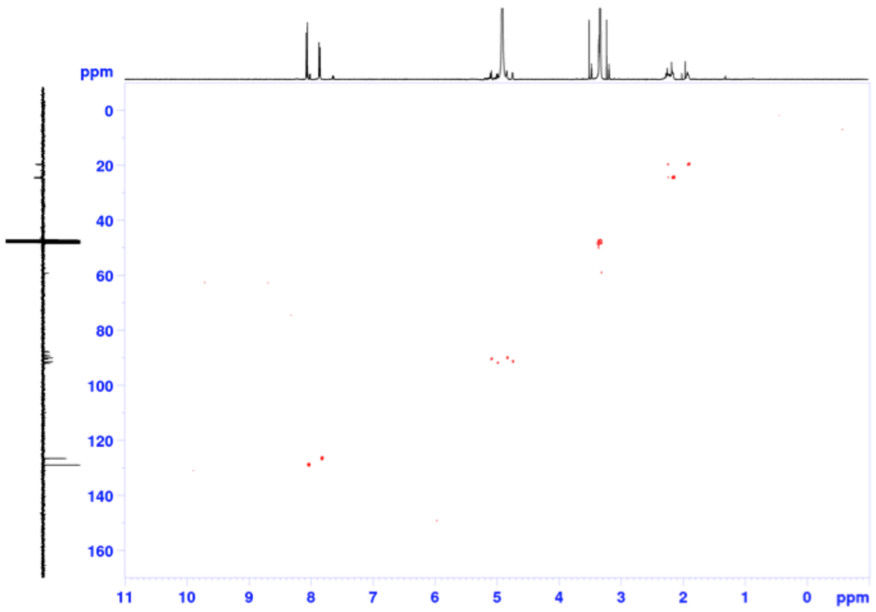
ppm
0
20
40
60
80
100
120
140
160
11
10
9
8
7
6
5
4
3
2
1
0
ppm

HMBC

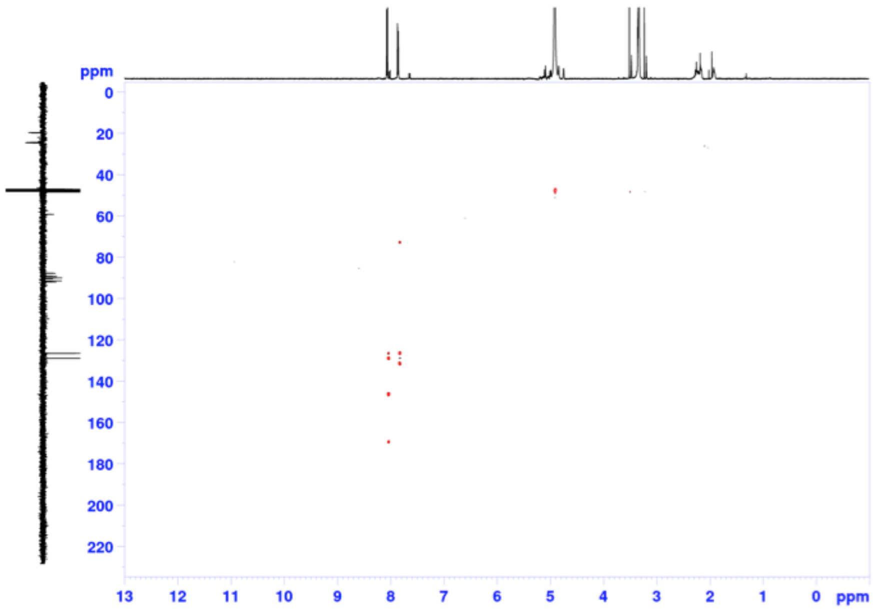
ppm
0
20
40
60
80
100
120
140
160
180
200
220
13
12
11
10
9
8
7
6
5
4
3
2
1
0
ppm

9. Single crystal X-Ray structure analysis

Single crystal X-Ray structure analysis was conducted by Prof. Alexandra M. Z. Slawin and Dr. David B. Cordes at the University of St Andrews.

9.1 2,3,5,6-Tetrafluoro-1-phenylcyclohexan-1-ol (8)

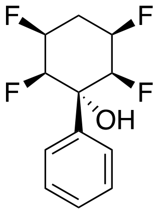

8

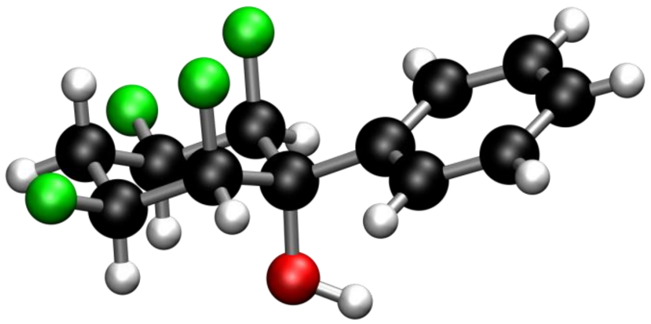

Empirical formula: **$C_{12}H_{12}F_4O$**

Unit cell parameters:

a = 6.0422(12) Å
b = 11.778(3) Å
c = 15.448(3) Å
V = 1099.4(4) Å^3

9.2 2,3-Difluoro-4-phenylcyclohexan-1-ol (11)

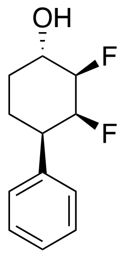

11

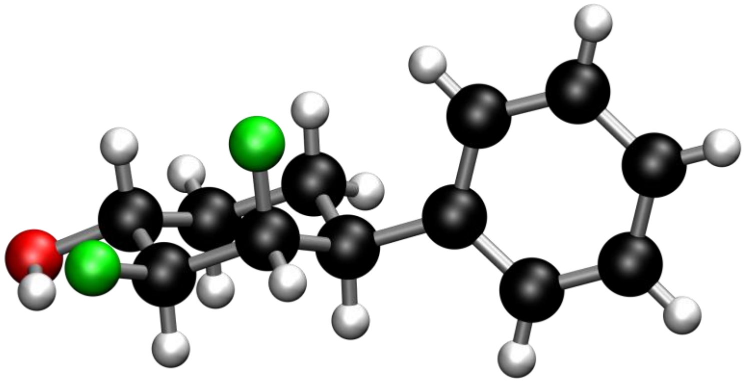

Empirical formula: $\mathbf{C_{12}H_{14}F_2O}$

Unit cell parameters:

a = 13.5173(3) Å

b = 5.57303(15) Å β = 90.469(2)^{o}

c = 27.4909(9) Å

V = 2070.88(10) Å^3

9.3 3,4-Difluoro-5-phenylcyclohexan-1-ol (12a)

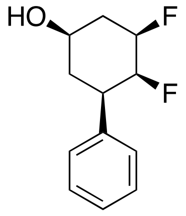

12a

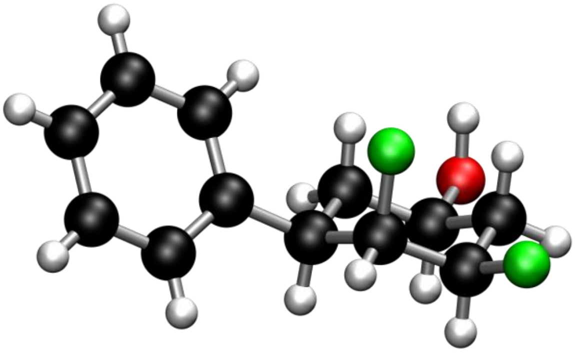

Empirical formula: $\mathbf{C_{12}H_{14}F_2O}$

Unit cell parameters:

a = 5.25889(6) Å
b = 9.75241(11) Å
c = 20.4698(2) Å
V = 1049.83(2) Å^3

9.4 3,4-Difluoro-5-phenylcyclohexan-1-ol (12b)

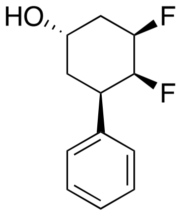

12b

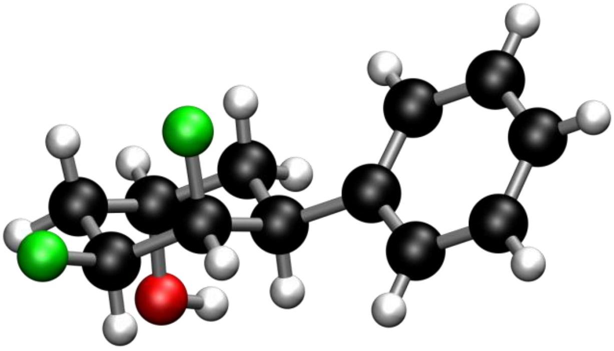

Empirical formula: $\mathbf{C_{12}H_{14}F_2O}$

Unit cell parameters:

a = 10.536(3) Å

b = 19.983(6) Å β = 107.972(7)°

c = 10.272(3) Å

V = 2057.2(10) Å^3

9.5 4-fluoro-3-phenylcyclohexan-1-ol (13)

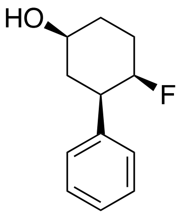

13

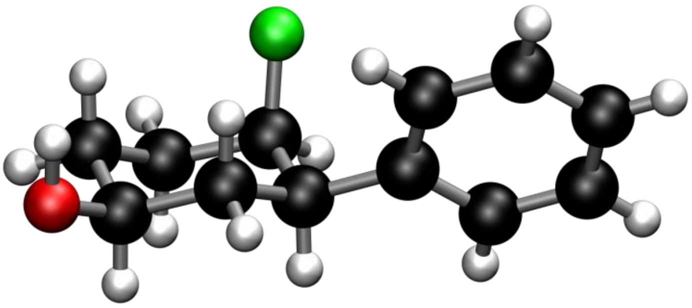

Empirical formula: $\mathbf{C_{12}H_{15}FO}$

Unit cell parameters:

a = 5.33024(13) Å
b = 9.6528(3) Å
c = 20.0332(7) Å
V = 1030.74(5) Å^3

10. References

1 a) A. J. Durie, T. Fujiwara, R. Cormanich, M. Bühl, A. M. Z. Slawin and D. O'Hagan, *Chem. Eur. J.* 2014, **20**, 6259; b) A. J. Durie, T. Fujiwara, N. Al-Maharik, A. M. Z. Slawin and D. O'Hagan, *J. Org. Chem.* 2014, **79**, 8228; c) M. S. Ayoup, D. B. Cordes, A. M. Z. Slawin and D. O'Hagan, *Org. Biomol. Chem.* 2015, **13**, 5621.

2 a) R. Tomita, N. Al-Maharik, A. Rodil, M. Bühl, D. O'Hagan, *Org. Biomol. Chem.* 2018, DOI: 10.1039/c7ob02987j. b) C. Giaginis and A. Tsantili-Kakoulidou, *J Liq Chromatogr Relat Technol.*, 2008, **31**, 79; c) C. My Du, K. Valko, C. Bevan, D. Reynolds and M. H. Abraham, *Anal. Chem.,* 1998, **70**, 4228.

3 a) J. Sangster, *J. Phys. Chem. Ref. Data*, 1989, **18**, 1111. b) C. Hansch, A. Leo, D. Hoekman, *Exploring QSAR – Hydrophobic, Electronic, and Steric Constants*, Washington, DC: American Chemical Society, 1995 c) P. C. von der Ohe, R. Kühne, R.-U. Ebert, R. Altenburger, M. Liess, G. Schüürmann, *Chem. Res.Toxicol.* 2005, **18**, 536.

4 P. Dawar, M. B. Raju, R. A. Ramakrishna, *Synthetic Communications*, 2014, **44**, 836.

5 a) D. Weininger, *J. Chem. Inf. Model*, 1988, **28**, 31 b) N. M. O'Boyle, M. Banck, C. A. James, C. Morley, T. Vandermeersch, G. R. Hutchison, *J. Cheminform.* , 2011, **3**, 33.

6 Schrödinger release 2015-2: Maestro, version 10.2, Schrödinger, llc, New York, NY, 2015.

7 a) D.A. Case, V. Babin, J.T. Berryman, R.M. Betz, Q. Cai, D.S. Cerutti, T.E. Cheatham, III, T.A. Darden, R.E. Duke, H. Gohlke, A.W. Goetz, S. Gusarov, N. Homeyer, P. Janowski, J. Kaus, I. Kolossvary, A. Kovalenko, T.S. Lee, S. LeGrand, T. Luchko, R. Luo, B. Madej, K.M. Merz, F. Paesani, D.R. Roe, A. Roitberg, C. Sagui, R. Salomon-Ferrer, G. Seabra, C.L. Simmerling, W. Smith, J. Swails, R.C. Walker, J. Wang, R.M. Wolf, X. Wu and P.A. Kollman AMBER 14, 2014. University of California, San Francisco b) A. Jakalian, B. L. Bush, D. B. Jack, C. I. Bayly, *J. Comput. Chem.,* 2000, **21**, 132.

8 W. L. Jorgensen, J. Chandrasekhar, J. D. Madura, R. W. Impey, M. L. Klein., *J. Chem. Phys.,* 1983, **79 (2),** 926.

9 a) W. L. Jorgensen, J. K. Buckner, S. Boudon, J. Tirado-Rives., *J. Chem. Phys.*, 1988, **89 (6),**3742. b) M. K. Gilson, J. A. Given, B. L. Bush, J. A. McCammon, *Biophys. J.,* 1997,**72 (3),**1047 c) S. Bosisio, A. S. J. S. Mey, J. Michel, *J. Comput. Aided Mol. Des.*, 2016, **30 (11),** 1101.

10 a) M. R. Shirts, J. D. Chodera, *J. Chem. Phys*, 2008, **129(12),** 124105 b) M. R. Shirts, D. L. Mobley, J. D. Chodera, V. S. Pande, *J. Phys. Chem. B*, 2007**, 111(45),** 13052.

11 G. Gerogiokas, G. Calabro, R.H. Henchman, M. W. Y. Southey, R. J. Law, J. Michel, *J. Chem. Theory Comput.,* 2013, **10(1),** 35.
